# Clinical features of aseptic meningitis with varicella zoster virus infection diagnosed by next-generation sequencing: case reports

**DOI:** 10.1186/s12879-020-05155-8

**Published:** 2020-06-22

**Authors:** Lanlan Chen, Yao Xu, Chunfeng Liu, Hong Huang, Xingxing Zhong, Cancan Ma, Haina Zhao, Yingzhu Chen

**Affiliations:** 1 Department of Neurology, Northern Jiangsu People’s Hospital, Medical College of Yangzhou University, Yangzhou, 225001 China; 2 Department of Neurology, the Second Affiliated Hospital of Soochow University, Suzhou, 215004 China; 3 Vision Medical Co., Ltd, Guangzhou, 510670 China

**Keywords:** Next-generation sequencing, Varicella zoster virus, Cerebrospinal fluid, Aseptic meningitis, Case report

## Abstract

**Background:**

The aseptic meningitis caused by varicella zoster virus (VZV) reactivation was less described in the literature, most of which were detected by means of polymerase chain reaction. The authors presented 4 adult immunocompetent patients with acute aseptic meningitis with VZV infection diagnosed by next-generation sequencing (NGS).

**Case presentation:**

Four patients were admitted to the hospital with headache and fever between March 2018 and August 2019. The median ages were 37 years (range 22–52 years). The median symptoms onset to clinic time was 3.5 days (range 3–6 days). Two patients had signs of meningeal irritation. Rash occurred after the meningitis symptoms in 1 patient (time from meningitis symptoms to rash, 2 days). No other sign or symptom was reported. The brain Magnetic resonance imaging and electroencephalography were normal in all patients. Cerebrospinal fluid (CSF) samples were obtained at a median of 4 days (range 3–7 days) from the meningitis symptoms onset. Opening pressure of lumbar puncture after admission were high in these cases (median 256 mm H_2_O; range 165–400 mm H_2_O). White blood cell counts and protein levels were significantly elevated in CSF samples (median 317 × 10^6/L, range 147–478 × 10^6/L; median 1.41 g/L, range 0.57–1.79 g/L). The cytology of CSF demonstrated a lymphocytic pleocytosis, and most multinuclear cells. The culture of CSF was negative for all 4 cases, while T-cell spot test was positive for 2 cases, who were administrated with anti-tuberculosis treatment for suspicious tuberculous meningitis. NGS of CSF (the Vision Medical Research Institute) detected specific sequences of VZV in the 4 cases within 72 h after admission. The inappropriate treatment were stopped while acyclovir were continued intravenously for 10–14 days. All patients recovered completely.

**Conclusions:**

VZV is an infectious agent that causes aseptic meningitis in immunocompetent adults and could not be accompanied by skin manifestations. The NGS of CSF is a rapid detection for the identification and differentiation of meningitis in patients, which is of great importance for providing the rapid and accurate diagnosis and the targeted antimicrobial therapy for central nervous system infection.

## Background

Aseptic meningitis is an inflammation of the meninges associated with acute onset of headache, fever and neck stiffness, with pleocytosis of the cerebrospinal fluid, and no growth on routine bacterial culture [1]. The leading recognizable causes of aseptic meningitis include non-polio human enteroviruses, mumps virus, lymphocytic choriomeningitis virus and herpesviruses [2]. Varicella zoster virus (VZV) reactivation is recognized as one of the most common neurological infectious diseases and VZV the second most frequent virus causing encephalitis [3]. VZV meningitis were less described in the literature, most of which involved adolescent or elderly patients and meanwhile the viruses were detected by means of polymerase chain reaction (PCR) [4].

We here described cases of 4 immunocompetent adults with aseptic meningitis due to VZV reactivation diagnosed by next-generation sequencing (NGS).

## Case presentation

### Case no. 1

On March 2018, a 52-year-old man was admitted to Northern Jiangsu People’s Hospital because of headache for 4 days and fever (37.6 °C) for 2 days. He had no other symptoms. The constant and progressive headache did not relieved after the routine treatment such as oral nonsteroidal analgesics. He had a history of chicken pox when he was 7 years old. He had symptomatic epilepsy since 8 years ago after a brain trauma, which occurred every 6 to 12 months. He had no sign of meningeal irritation, and vital signs were normal. No rash was seen at any stage (Table 1).

**Table 1:** Demographic characteristics and clinical findings for cases

Case No.	Gender	Age (y)	Duration of symptome before admittion (d)	Duration of hospitalization (d)	Headache	Fever	Cutaneous zoster	Neck stiffness	Kernig signs	EEG	MRI	History of chicken pox
1	male	52	4	10	+	+	–	–	–	–	–	+
2	male	22	3	10	+	+	+	+	+	–	–	+
3	male	45	6	14	+	+	–	–	–	–	–	uncertain
4	male	29	3	10	+	+	–	+	–	–	–	+

**Fig. 1:**
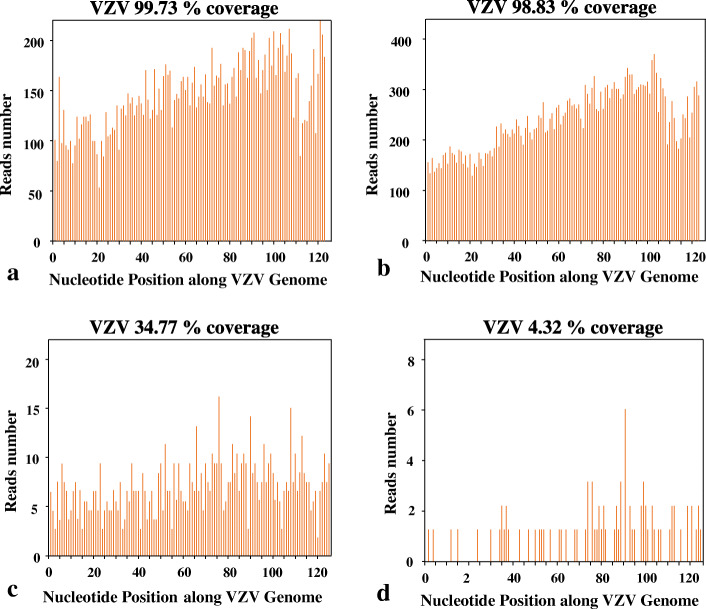
Reads mapping to VZV genome, generated with NGS from CSF. **a** In case No. 1, the viral reads (17,137 reads) corresponded to varicella zoster virus, with a genome coverage of 99.73%. **b** In case No. 2, the viral reads (13,190 reads) corresponded to varicella zoster virus, with a genome coverage of 99.83%. **c** In case No. 3, the viral reads (1330 reads) corresponded to varicella zoster virus, with a genome coverage of 34.77%. **d** In case No. 4, the viral reads (102 reads) corresponded to varicella zoster virus with a genome coverage of 4.32%

The white blood cell count was 11.23 × 10^9/L with 79% neutrophils. The serum electrolytes, hemoglobin, erythrocyte sedimentation rate, procalcitonin, and C-reactive protein were normal. The T-cell spot test was positive. The cerebrospinal fluid (CSF) contained 147 × 10^6/L white blood cells (WBCs) (reference range, <8 × 10^6/L); CSF protein was 1.72 g/L (reference range, 0.2–0.4 g/L) (Table 2). Brain Magnetic resonance imaging (MRI) revealed pre-existing brain trauma lesions. The patient was started on intravenous acyclovir for possible herpes simplex virus infection, ceftriaxone for possible bacterial disease, as well as rifampin, isoniazid, pyrazinamide, and ethambutol for the possible tuberculosis infection. His CSF sample was immediately sent for pathogen detection by NGS at Vision Medical Research Institute, and it was sequenced on Illumina NextSeq500 platform using a 75-cycle single-end (see Additional file 1 for detailed detection process) [5]. The identified number of unique reads mapped on the VZV genome sequence was 17,137, with genome coverage of 99.73%, and read depth of 17.9 X (Table 3, Fig. 1). After filtering out low-complexity and shorter reads, the NGS analyses of other viruses, bacterial, mycoplasma, and tubercle bacillus were negative (Additional file 2).

**Table 2:** Routine laboratory evalutions of CSF of the four cases

Case No.	Days from symptom onset to CSF collection (d)	Days from CSF collection to diagnosis (d)	Total time to diagnosis (d)	Pressure (mm H_2_O)	WBC (×10^6^/L)	Multinuclear (%)	Protein (g/L)	Glucose (mmol/L)	Serum glucose (mmol/L)
1	4	2	2	165	147	1.4	1.72	3.82	6.80
1	13	/	/	120	39	1.0	0.88	4.97	6.95
2	3	2	2	235	200	1.0	0.57	3.88	4.74
2	11	/	/	70	130	0.8	0.40	4.97	5.93
3	7	2	3	400	478	0.2	1.79	7.03	8.37
3	16	/	/	240	91	0.4	0.65	4.06	5.79
4	4	2	3	275	434	0.7	1.10	3.72	4.77
4	12	/	/	175	175	0.9	0.37	3.42	4.64

Note: Total time to diagnosis = days to CSF collection+ days from CSF collection to NGS result

**Table 3:** Number, percentage, genome coverage, and read depth of unique reads for the sequences of varicella zoster virus in the CSF samples

Case No.	Pathogen	Unique reads	Percentage, %	Genome Coverage, %	Read Depth (X)
1	varicella zoster virus	17,137	100	99.73	17.90
2	varicella zoster virus	13,190	100	98.83	11.87
3	varicella zoster virus	1330	100	34.77	1.25
4	varicella zoster virus	102	96.23	4.32	1.03

Note: In case 1, 2, and 3, only VZV has been detected by NGS. So, the percentage is 100% for each of them. In case 4, there were 102 reads of VZV and 4 reads of Human parvovirus B19 detected by NGS (Additional file 5). So, the percentage is 96.23% (102/106) for case 4

After the diagnosis with VZV meningitis within 48 h after admission, the antibiotic and anti-tuberculosis treatment were stopped immediately. The acyclovir (10 mg/kg t.i.d) were continued intravenously for 10 days. The patient’s condition improved quickly. A repeat lumbar puncture on the ninth day after admission revealed improved WBCs (39 × 10^6/L) and protein (0.88 g/L) (Table 2). Thus he was discharged, and a month later on follow-up in the outpatient clinic, he had recovered completely.

### Case no. 2

On November 2018, a 22-year-old man was admitted to Northern Jiangsu People’s Hospital because of fever (37.7 °C), severe headache, and nausea for 3 days. One day before admission, he noticed vescicles in a very limited region of the left dorsal skin. No other sign or symptom was reported (Table 1). He was diagnosed of varicella when he was 4 years old. Upon admission, his vital signs were unremarkable, and the skin lesions located in a small part of the area supplied with level of 10 thoracic were considered consistent with a diagnosis with herpes zoster. Neurological examination was significant for neck stiffness and positive for Kernig signs.

The white blood cell count was 12.18 × 10^9/L, while 81% neutrophils. The inflammatory biomarkers, same with those tested in case 1, were within normal ranges. The opening pressure of lumbar puncture was 235 mm H_2_O (reference range, 90–180 mm H_2_O). The cytology of CSF demonstrated WBCs with 200 × 10^6/L, and an increased protein concentration (0.57 g/L) (Table 2). The CSF was positive for VZV by NGS (Vision Medical Research Institute), with unique reads of sequences of 13,190, the genome coverage of 98.83%, and read depth of 11.87 X (Table 3, Fig. 1, Additional file 3).

The patient was treated with acyclovir and ceftriaxone after admission. After VZV was detected, ceftriaxone was stopped and acyclovir (10 mg/kg t.i.d) was continued intravenously for 10 days. The therapy was highly effective and the patient’s clinical condition rapidly improved. The CSF on the eighth day after the admission revealed WBCs with 130 × 10^6/L, and protein (0.40 g/L) (Table 2). The patient was discharged after 10-day treatment. An examination carried out about 4 weeks later did not find any sign or symptom of disease.

### Case no. 3

On August 2019, a 45-year-old man with headache for 6 days was admitted to our hospital. He felt fatigued and headache, never measured his temperature. No other sign or symptom was reported. No positive sign of neurological examination was detected (Table 1). A low fever of less than 38.0 °C (37.8 °C on the first day, 37.6 °C on the second day, 37.7 °C on the third day) was found every afternoon since the second day after admission. The white blood cell count was 13.45 × 10^9/L, while 79% neutrophils. The inflammatory biomarkers, same with those tested in case 1 and case 2, were within normal ranges. The lumbar puncture on the second day after admission revealed significantly increased opening pressure of CSF (400 mm H_2_O). The CSF contained 478 × 10^6/L WBCs; CSF protein was 1.79 g/L (Table 2). The T-cell spot test was positive. The patient was treated with intravenous mannitol, acyclovir, ceftriaxone, as well as rifampin, isoniazid, pyrazinamide, and ethambutol for the possible tuberculosis infection. His CSF sample was immediately tested by NGS at Vision Medical Research Institute. The sequencing detection identified 1330 sequence reads uniquely corresponding to the VZV, with the genome coverage of 34.77% (Table 3, Fig. 1, Additional file 4).

After etiological diagnosis was identified, antibiotic and anti-tuberculosis treatment were stopped immediately. The intravenously acyclovir was continued for total 14 days, while mannitol was for the total 11 days with a gradually decreased dose. The disease made rapid progress. A repeat lumbar puncture on the eleventh day after admission revealed improved WBCs (91 × 10^6/L) and protein (0.65 g/L) (Table 2). Thus he was discharged from hospital. A month later on follow-up in the outpatient clinic, he had recovered completely.

### Case no. 4

On August 2019, a 29-year-old man with a fever (38.0 °C) and a progressive headache was admitted to Northern Jiangsu People’s Hospital. His disease began with headache and nausea 3 days earlier. No other symptom was developed. Neurological examination was moderate for neck stiffness and negative for Kernig signs (Table 1). The white blood cell count was 13.73 × 10^9/L, while 82% neutrophils. The results of routine inflammatory biomarkers, same with those tested in other cases, were normal. The second day after the admission, the opening pressure of lumbar puncture showed 275 mm H_2_O, and the CSF contained 434 × 10^6/L WBCs (Table 2). The patient was started on intravenous acyclovir of 10 mg/kg t.i.d and mannitol of 250 ml b.i.d. DNA of VZV was identified in the CSF using NGS (Vision Medical Research Institute). The sequencing detection identified 102 sequence reads uniquely corresponding to the VZV, with genome coverage of 4.32% (Table 3, Fig. 1, Additional file 5). The intravenous acyclovir were administered for 10 days, and the patient’s condition improved quickly. A repeat lumbar puncture on the eighth day after the admission revealed normal pressure of 175 mm H_2_O, improved WBCs (129 × 10^6/L), and normal protein (0.37 g/L) (Table 2). Thus he was discharged from hospital. A month later on follow-up in the outpatient clinic, he had recovered completely.

**Fig. 2:**
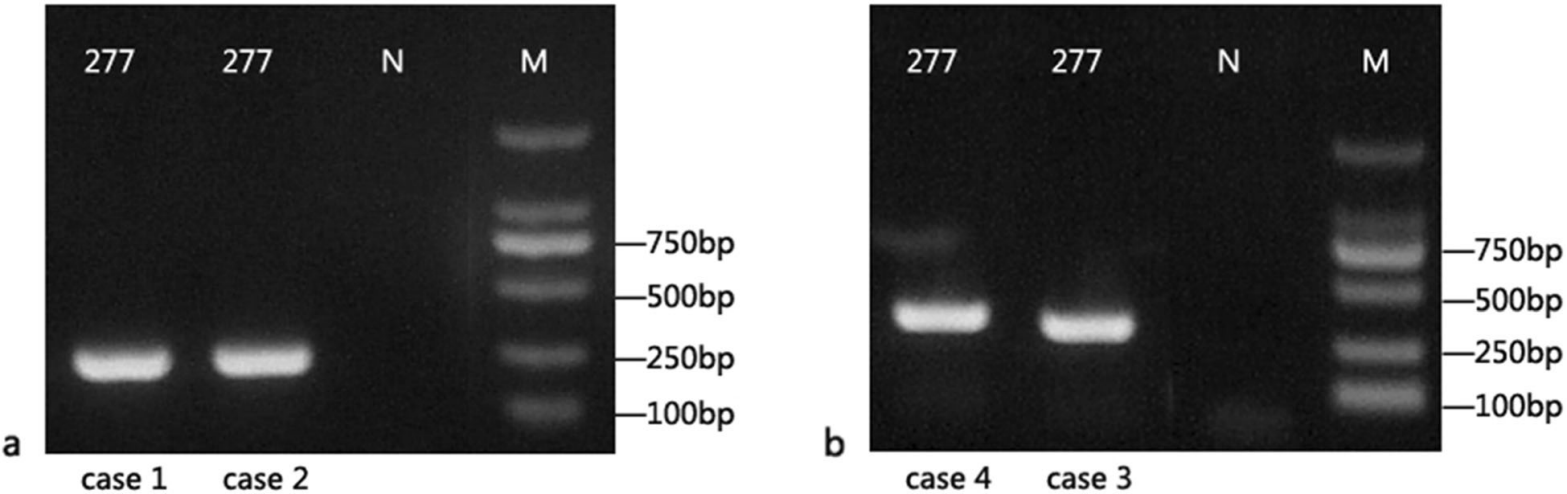
Sanger sequencing detection of VZV from CSF specimen. M: DNA markers of DL2000 or Trans 2 K Plus. N: negative control. The number 277 bp represent the sample code. Original images were in Additional file 6

The sequence-specific PCR identification of VZV was carried out to validate the NGS results for the 4 cases. The specific primers used for the gene amplification were VZV-F2 (GACAATATCATATACATGGAATGTG) and VZV-R2 (GCGGTAGTAACAGAGAATTTCTT). The results showed that the read from Sanger sequencing was consistent with VZV genome (see Additional file 1 for PCR results and Additional file 6 for original images) (Fig. 2).

## Discussion and conclusions

VZV, belonging to the group of alpha-herpes viruses, causes varicella (chickenpox) and herpes zoster. Varicella usually results in mild to moderate illness in mainly childhood or immunocompetent patients with disseminated vesicular rash. After primary infection, VZV remains latent in sensory cranial nerve ganglia or dorsal root ganglia, when reactivated, replicates along the course of the nerve and appears as a localized vesicular skin rash. Viral reactivation can cause a wide range of neurologic disease, most frequently manifesting as herpes zoster and post-herpetic neuralgia [3]. Older age, immunocompromised state, bone marrow transplant recipients and possibly pregnancy are risk factors associated with higher severity of VZV. Aseptic meningitis is usually regarded as an uncommon complication of the cutaneous primary infection in patients with impaired cellular immunity. Recently, many studies reported that VZV was an important cause of aseptic infection in central nervous system (CNS), with the frequencies ranging from 5 to 27% [5, 6].

The etiology of acute meningoencephalitis remains undiagnosed in approximately 60% of cases despite extensive clinical laboratory testing for infectious pathogens [7]. Till now, many diagnostic assays are based on polymerase chain reaction (PCR), which relies on sequence-specific primers. NGS has been applied as a diagnostic method to detect the pathogens for CNS infectious diseases in recent years. Many successful applications of NGS to the diagnosis with CNS infections have been reported [8, 9]. The case series included 4 male adult patients who were diagnosis with VZV meningitis by NGS. Interestingly, all 4 patients presenting in this study were less than 50 years of age, although VZV reactivation were reported more commonly occurred in older adults [10]. This finding was similar to that in another study in which 5 of the 8 patients with VZV meningitis were presented before the fifth decade of life. None of them had traditional risk factors for VZV infection. The only one who had epilepsy history took antiepileptic drugs, rather than immunosuppressant drugs, which indicated that reactivation of VZV may be a more frequent cause of aseptic meningitis than previously anticipated in immunocompetent individuals. Koskiniemi et al. reported that 27 and 65% of patients with encephalitis and meningitis, respectively, had no skin manifestations, suggesting VZV could reactivate independently of vesicular eruptions, and spread directly to the leptomeninges [11]. In the present study, 3 patients (75%) had no cutaneous zosteriform lesions, which was consistence with the previous study [11, 12].

Lumbar puncture revealed increased opening pressure of CSF (235–400 mm H_2_O) in three patients, and it was also on the high side (165 mm H_2_O) for the other one. All cases showed elevation in CSF WBC, ranging from 147 to 478 × 10^6^/L, and the CSF cytology indicated lymphocytic inflammation. It was regarded that mild elevation in CSF protein levels would been observed in aseptic meningitis. However, the relatively high CSF protein levels were reported seen in patients with VZV infection which were significantly higher than those seen in patients with enteroviral infection [12, 13]. Protein levels (median 1.41 g/L) in this study were higher than previously reported. All patients received acyclovir intravenously for 1–2 weeks resulting in full recovery, suggesting that VZV meningitis tends to be mild symptom, good response to treatment and benign prognosis.

The difficulty in meningitis diagnosis is to distinguish whether it’s a viral or bacterial etiology, because this is crucial for treatment decisions. The treatment threshold is usually set low in clinical work, so that antibiotics, even anti-tuberculosis treatment, are often prescribed in cases of doubt, as were patients in this study. The patient of case No. 2 had vesicular rash before admission which made the diagnosis with aseptic meningitis relatively easy. However, the other 3 patients never showed cutaneous zosteriform lesions. Additionally, the T-cell spot test was positive for the second and fourth cases, meanwhile the protein levels in CSF elevated significantly for both these 2 patients, which implied possible tuberculosis infection, therefore anti-tuberculosis treatment as well as antibiotics were administrated. However, all the cases in our study were pathogen diagnosis within 72 h after admission. Once the sequences of VZV were detected by NGS, inappropriate treatment were stopped.

NGS is a rapid and accurate approach for the molecular diagnosis with diseases compared to traditional clinical testing. It could dramatically reduce the diagnostic period to less than 3 days [14]. Pathogen-specific PCR is widely used to detect common viruses like herpes simplex virus, VZV and enterovirus because of its high sensitivity and specificity. The turn-around time, which is the time taken from CSF collection to receipt result report, is often 2 days for PCR, and is comparable to that of the NGS test. Most centers around the world would consider NGS only for samples that have been tested negative by pathogen-specific PCR. However, our center opted for direct NGS instead of PCR in this study, for 3 of the cases were difficult to distinguish between viral and bacterial etiology from CSF characteristic or clinical feature. The main limitation of PCR is the level to which assays can be multiplexed, which constrains the number of targets that can be assessed per reaction. For cases of common virus infection, the diagnosis will be quickly confirmed by PCR; for cases of other microorganism infection which are out of the PCR test range, the diagnosis time would be extended. In contrast, NGS is a high-throughput approach that can interrogate all genetic material in a biologic sample simultaneously [15]. It enable sequencing the total DNA or ribonucleic acid (RNA) from a human sample and identify all possible microorganism present in the specimen. Besides, NGS is an untarged assay as it can amplify and sequence the entire DNA content of a sample without using any primers or probes. The NGS results were further validated by Sanger sequencing in our cases, which was consistent with our expectation, and indicated the reliability of the results and the great practical guiding value of NGS.

VZV reactivation leading to aseptic meningitis in immunocompetent adults with or without cutaneous zoster is more common that previous regarded. Relatively high CSF protein levels could be observed in VZV meningitis. This study highlighted the feasibility of using NGS of CSF as a diagnostic tool for CNS infection. Unbiased NGS could facilitate identification of all the potential pathogens in a single assay theoretically, which is of great importance for providing the rapid and accurate diagnosis and the targeted antimicrobial therapy for CNS infection.

## Supplementary information

**Additional file 1.** Details of NGS detection process and the results of Sanger sequencing

**Additional file 2.** Microbe reads of bacterium, fungi, parasite and virus detected in Case No. 1

**Additional file 3.** Microbe reads of bacterium, fungi, parasite and virus detected in Case No. 2

**Additional file 4.** Microbe reads of bacterium, fungi, parasite and virus detected in Case No. 3

**Additional file 5.** Microbe reads of bacterium, fungi, parasite and virus detected in Case No. 4

**Additional file 6.** Original images for results of Sanger sequencing detection of VZV from CSF specimen

## Data Availability

All data generated or analysed during this study are included in this published article**.** The CSF was detected by mNGS (illumine Nextseq 550, Vision Medical Research Institute). The VZV DNA sequence assembled using the NGS data was submitted to GenBank (accession no. KY062165). Other microbe reads including that of bacterium, fungi, parasite and virus were listed in Additional files 2–5.

## Abbreviations

CNS: Central nervous system
CSF: Cerebrospinal fluid
DNA: Deoxyribonucleic acid
EEG: Electroencephalography
MRI: Magnetic resonance imaging
NGS: Next-generation sequencing
PCR: Polymerase chain reaction
RNA: Ribonucleic acid
VZV: Varicella zoster virus
WBC: White blood cells
