# Supplementary material for: Clinical features of aseptic meningitis with varicella zoster virus infection diagnosed by next-generation sequencing: case reports
Source: BMC Infect Dis. 2020 Jun 22;20:435. doi: 10.1186/s12879-020-05155-8 (PMC7309994; doi:10.1186/s12879-020-05155-8)
Supplement: Supplementary file 1 — Additional file 1. Details of NGS detection process and the results of Sanger sequencing [file 12879_2020_5155_MOESM1_ESM.docx]

1. **Methods of next-generation sequencing**

The methodology about NGS in this manuscript is based on the Methods in this reference:

Blauwkamp TA, Thair S, Rosen MJ, et al. Analytical and clinical validation of a microbial cell-free DNA sequencing test for infectious disease. Nat Microbiol, 2019,4(4):663-674.

**Sample collection and information**

The CSF of the 4 patients were collected from the Department of Neurology in Northern Jiangsu People’s Hospital according to standard procedures (Case report, Case No. 1, paragraph 2; Case No. 2, paragraph 1; Case No. 3, paragraph 1; Case No. 4, paragraph 1). The 4 CSF samples (each more than 2 milliliter) were immediately sent for pathogen detection by next-generation sequencing (NGS) at Vision Medicals. The patients had signed informed consent, and samples were used for research only. The Institutional Review Board of Northern Jiangsu People’s Hospital approved this study. The Ethics Committee of Northern Jiangsu People’s Hospital approved the use of human subjects for this study. All the patients provided written (signed) informed consent to participate in this study.

**DNA extraction, library preparation, and sequencing** (Case report, Case No. 1, paragraph 2; Case No. 2, paragraph 1; Case No. 3, paragraph 1; Case No. 4, paragraph 1)

CSF sample from patient was collected according to standard procedures, and centrifuged at 2000 RPM for 5 min. 300 uL supernatant were subjected to nucleotides purification with TIANamp Micro DNA Kit (DP316, TianGen Biotech) according to the manufacturer’s recommendation.

DNA were sheared under power 50 for 155 seconds by using focused-ultrasonicators (Covaris). Total 100 ng sheared DNA were subjected to library construction with VAHTS Universal DNA Library Prep Kit for Illumina V3 kit (ND607-2, Vazyme Biotech). After purification and sorting, the DNA length of the library was profiled by Agilent 2100 bioanalyzer. Library with 300±50 bp peak passed and was subjected to library pooling. Pooled libraries were diluted and up to 24 libraries per batch were multiplexed and subjected to Illumina NextSeq 550 sequencing platform using a 75-cycle single-end, following manual instruction.

**Data treatment and analysis** (Case report, Case No. 1, paragraph 2; Case No. 2, paragraph 1; Case No. 3, paragraph 1; Case No. 4, paragraph 1)

High-quality sequencing data were generated by removing low-quality, and short (length < 35bp) reads, followed by computational substraction of human host sequences mapped to the human reference genome (hg19) using Burrows-Wheeler Alignment. The remaining data by removal of low-complexity reads were classified by simultaneously aligning to four Microbial Genome Databases, consisting of viruses, bacteria, fungi, and parasites. The classification reference databases were downloaded from NCBI (ftp://ftp.ncbi.nlm.nih.gov/genomes/). RefSeq or Genebank contains 5348 whole genome sequence of viral taxa, 4170 bacterial genomes or scaffolds, 393 fungi related to human infection, and 140 parasites associated with human diseases.

1. **The results of Sanger sequencing**

The Sanger sequencing identification of VZV was carried out to validate the NGS results for the 4 cases. The specific primers used for the gene amplification were VZV-F2 (GACAATATCATATACATGGAATGTG) and VZV-R2 (GCGGTAGTAACAGAGAATTTCTT). The results showed that the read from Sanger sequencing was consistent with VZV genome (Fig. 2).

**The result for case no. 1**

>TGACAATATCAATACATGGAATGTGTTAAAGCGGGGGTCAAACTTATCCC

CACGAAAGTCGATTTCCCCCCAAATATTCACGCGTCTAGGCCAGGGGCTG

GAACAACGAAAATCCAGAATCGGAACTTCTTTTCCATTACAGTAAACTTT

AGGCGGTCGACTAAGTGTACCGACGTGAACCCCCTTTCGTTCTTCCATGG

GCACATCTTCATCTAAACATTTAGGGGCCAAAAATTGAAACGATGACATG

GTAGTTTTGTAACTATGAAGAAATTCTCTGTTACTACCGCA

**The result for case no. 2**

>TGACAATATCAATACATGGAATGTGTTAAAGCGGGGGTCAAACTTATCCC

CACGAAAGTCGATTTCCCCCCAAATATTCACGCGTCTAGGCCAGGGGCTG

GAACAACGAAAATCCAGAATCGGAACTTCTTTTCCATTACAGTAAACTTT

AGGCGGTCGACTAAGTGTACCGACGTGAACCCCCTTTCGTTCTTCCATGG

GCACATCTTCATCTAAACATTTAGGGGCCAAAAATTGAAACGATGACATG

GTAGTTTTGTAACTATGAAGAAATTCTCTGTTACTACCGCA

**The result for case no. 3**

>TACATGGAATGTGTTAAAGCGGGGGTCAAACTTATCCCCACGAAAGTCGATTTCC

CCCCAAATATTCACGCGTCTAGGCCAGGGGCTGGAACAACGAAAATCCAGAATC

GGAACTTCTTTTCCATTACAGTAAACTTTAGGCGGTCGACTAAGTGTACCGACGTGAA

CCCCCTTTCGTTCTTCCATGGGCACATCTTCATCTAAACATTTAGGGGCCAAAAATTGAAACGATGACATGGTAGTTTTGTAACTATGAAGAAATTCTCTGTTACTACCG

**The result for case no. 4**

>ATACATGGAATGTGTTAAAGCGGGGGTCAAACTTATCCCCACGAAAGTCGATTTC

CCCCCAAATATTCACGCGTCTAGGCCAGGGGCTGGAACAACGAAAATCCAGAATC

GGAACTTCTTTTCCATTACAGTAAACTTTAGGCGGTCGACTAAGTGTACCGACGTG

AACCCCCTTTCGTTCTTCCATGGGCACATCTTCATCTAAACATTTAGGGGCCAAAAA

TTGAAACGATGACATGGTAGTTTTGTAACTATGAAGAAATTCTCTGTTACTACCGCAA
