# Supplementary material for: Clinical features of aseptic meningitis with varicella zoster virus infection diagnosed by next-generation sequencing: case reports
Source: BMC Infect Dis. 2020 Jun 22;20:435. doi: 10.1186/s12879-020-05155-8 (PMC7309994; doi:10.1186/s12879-020-05155-8)
Supplement: Supplementary file 2 — Additional file 2. Microbe reads of bacterium, fungi, parasite and virus detected in Case No. 1 [file 12879_2020_5155_MOESM2_ESM.docx]

**Additional file 2:** Microbe reads of bacterium, fungi, parasite and virus detected in Case No. 1

　Table 1. Microbe reads of bacterium detected in Case No. 1

| Genus | Genus_Abs_Abu | SMRNG | SDSMRNG | Species | SMRN | SDSMRN | Coverage | CovRate | Depth |
| --- | --- | --- | --- | --- | --- | --- | --- | --- | --- |
| Ralstonia | 103.3262 | 560 | 537 | Ralstonia_insidiosa | 445 | 427 | 60799/5808308 | 1.05 | 1.01 |
| Ralstonia | 103.3262 | 560 | 537 | Ralstonia_mannitolilytica | 22 | 21 | 5348/4881769 | 0.1095 | 1 |
| Ralstonia | 103.3262 | 560 | 537 | Ralstonia_solanacearum | 6 | 6 | 1687/3984240 | 0.0423 | 1.05 |
| Ralstonia | 103.3262 | 560 | 537 | Ralstonia_pickettii | 0 | 0 | 102/8125850 | 0.0013 | 1 |
| Burkholderia | 32.5771 | 251 | 241 | Burkholderia_contaminans | 58 | 56 | 16776/8509269 | 0.1972 | 1.01 |
| Burkholderia | 32.5771 | 251 | 241 | Burkholderia_multivorans | 6 | 6 | 923/7281887 | 0.0127 | 1 |
| Burkholderia | 32.5771 | 251 | 241 | Burkholderia_ubonensis | 4 | 4 | 1214/8028779 | 0.0151 | 1 |
| Burkholderia | 32.5771 | 251 | 241 | Burkholderia_lata | 3 | 3 | 3897/8676277 | 0.0449 | 1 |
| Burkholderia | 32.5771 | 251 | 241 | Burkholderia_pyrrocinia | 3 | 3 | 907/7847882 | 0.0116 | 1 |
| Burkholderia | 32.5771 | 251 | 241 | Burkholderia_cenocepacia | 2 | 2 | 844/8531679 | 0.0099 | 1 |
| Burkholderia | 32.5771 | 251 | 241 | Burkholderia_CCGE1002 | 1 | 1 | 288/7395722 | 0.0039 | 1 |
| Burkholderia | 32.5771 | 251 | 241 | Burkholderia_dolosa | 1 | 1 | 202/6409115 | 0.0032 | 1 |
| Burkholderia | 32.5771 | 251 | 241 | Burkholderia_glumae | 1 | 1 | 314/6733840 | 0.0047 | 1 |
| Burkholderia | 32.5771 | 251 | 241 | Burkholderia_metallica | 1 | 1 | 755/7424260 | 0.0102 | 1 |
| Burkholderia | 32.5771 | 251 | 241 | Burkholderia_thailandensis | 1 | 1 | 104/6739510 | 0.0015 | 1 |
| Burkholderia | 32.5771 | 251 | 241 | Burkholderia_CCGE1001 | 0 | 0 | 83/6833751 | 0.0012 | 1 |
| Burkholderia | 32.5771 | 251 | 241 | Burkholderia_KJ006 | 0 | 0 | 558/6584551 | 0.0085 | 1 |
| Burkholderia | 32.5771 | 251 | 241 | Burkholderia_ambifaria | 0 | 0 | 385/7484986 | 0.0051 | 1 |
| Burkholderia | 32.5771 | 251 | 241 | Burkholderia_anthina | 0 | 0 | 653/7273081 | 0.009 | 1 |
| Burkholderia | 32.5771 | 251 | 241 | Burkholderia_cepacia | 0 | 0 | 577/8396158 | 0.0069 | 1 |
| Burkholderia | 32.5771 | 251 | 241 | Burkholderia_diffusa | 0 | 0 | 85/6857853 | 0.0012 | 1 |
| Burkholderia | 32.5771 | 251 | 241 | Burkholderia_gladioli | 0 | 0 | 221/8114449 | 0.0027 | 1 |
| Burkholderia | 32.5771 | 251 | 241 | Burkholderia_mallei | 0 | 0 | 82/5913144 | 0.0014 | 1 |
| Burkholderia | 32.5771 | 251 | 241 | Burkholderia_oklahomensis | 0 | 0 | 92/7313683 | 0.0013 | 1 |
| Burkholderia | 32.5771 | 251 | 241 | Burkholderia_phytofirmans | 0 | 0 | 101/8093536 | 0.0013 | 1 |
| Burkholderia | 32.5771 | 251 | 241 | Burkholderia_pseudomallei | 0 | 0 | 128/7446579 | 0.0017 | 1 |
| Burkholderia | 32.5771 | 251 | 241 | Burkholderia_stabilis | 0 | 0 | 514/8527967 | 0.006 | 1 |
| Burkholderia | 32.5771 | 251 | 241 | Burkholderia_vietnamiensis | 0 | 0 | 153/6827896 | 0.0022 | 1 |
| Bradyrhizobium | 39.8056 | 143 | 137 | Bradyrhizobium_japonicum | 21 | 20 | 11425/9207384 | 0.1241 | 1.01 |
| Bradyrhizobium | 39.8056 | 143 | 137 | Bradyrhizobium_BTAi1 | 13 | 12 | 5087/8264687 | 0.0616 | 1 |
| Bradyrhizobium | 39.8056 | 143 | 137 | Bradyrhizobium_ORS | 10 | 10 | 6015/7456587 | 0.0807 | 1.02 |
| Bradyrhizobium | 39.8056 | 143 | 137 | Bradyrhizobium_S23321 | 9 | 9 | 8291/7231841 | 0.1147 | 1.01 |
| Bradyrhizobium | 39.8056 | 143 | 137 | Bradyrhizobium_oligotrophicum | 7 | 7 | 5576/8264165 | 0.0675 | 1.02 |
| Propionibacterium | 48.6989 | 118 | 113 | Propionibacterium_acnes | 106 | 102 | 14172/2560282 | 0.5535 | 1 |
| Propionibacterium | 48.6989 | 118 | 113 | Propionibacterium_humerusii | 8 | 8 | 1146/2644116 | 0.0433 | 1 |
| Mesorhizobium | 23.3491 | 79 | 76 | Mesorhizobium_ciceri | 8 | 8 | 5792/6264489 | 0.0925 | 1.09 |
| Mesorhizobium | 23.3491 | 79 | 76 | Mesorhizobium_australicum | 6 | 6 | 2782/6200534 | 0.0449 | 1.01 |
| Mesorhizobium | 23.3491 | 79 | 76 | Mesorhizobium_loti | 2 | 2 | 4523/7036071 | 0.0643 | 1.04 |
| Mesorhizobium | 23.3491 | 79 | 76 | Mesorhizobium_opportunistum | 2 | 2 | 2916/6884444 | 0.0424 | 1.09 |
| Ochrobactrum | 21.663 | 30 | 29 | Ochrobactrum_intermedium | 11 | 11 | 6850/4665240 | 0.1468 | 1.08 |
| Ochrobactrum | 21.663 | 30 | 29 | Ochrobactrum_anthropi | 6 | 6 | 4213/4783208 | 0.0881 | 1.05 |
| Methylobacterium | 5.7017 | 24 | 23 | Methylobacterium_radiotolerans | 4 | 4 | 1103/6077833 | 0.0181 | 1.08 |
| Methylobacterium | 5.7017 | 24 | 23 | Methylobacterium_populi | 2 | 2 | 216/5800441 | 0.0037 | 1 |
| Methylobacterium | 5.7017 | 24 | 23 | Methylobacterium_aquaticum | 1 | 1 | 798/5348274 | 0.0149 | 1 |
| Methylobacterium | 5.7017 | 24 | 23 | Methylobacterium_mesophilicum | 1 | 1 | 480/6214729 | 0.0077 | 1 |
| Methylobacterium | 5.7017 | 24 | 23 | Methylobacterium_4 | 0 | 0 | 96/7659055 | 0.0013 | 1 |
| Methylobacterium | 5.7017 | 24 | 23 | Methylobacterium_brachiatum | 0 | 0 | 384/5807713 | 0.0066 | 1 |
| Methylobacterium | 5.7017 | 24 | 23 | Methylobacterium_chloromethanicum | 0 | 0 | 263/5777908 | 0.0046 | 1 |
| Methylobacterium | 5.7017 | 24 | 23 | Methylobacterium_extorquens | 0 | 0 | 274/5943768 | 0.0046 | 1 |
| Methylobacterium | 5.7017 | 24 | 23 | Methylobacterium_nodulans | 0 | 0 | 178/7772460 | 0.0023 | 1 |
| Rhizobium | 15.5301 | 23 | 22 | Rhizobium_leguminosarum | 6 | 6 | 2531/5119898 | 0.0494 | 1.03 |
| Rhizobium | 15.5301 | 23 | 22 | Rhizobium_tropici | 6 | 6 | 2690/3837060 | 0.0701 | 1.08 |
| Rhizobium | 15.5301 | 23 | 22 | Rhizobium_IRBG74 | 0 | 0 | 682/2844565 | 0.024 | 1 |
| Rhizobium | 15.5301 | 23 | 22 | Rhizobium_etli | 0 | 0 | 1023/4598466 | 0.0222 | 1 |
| Brucella | 22.6871 | 15 | 14 | Brucella_inopinata | 2 | 2 | 2180/3367314 | 0.0647 | 1.07 |
| Brucella | 22.6871 | 15 | 14 | Brucella_abortus | 0 | 0 | 300/3289510 | 0.0091 | 1 |
| Brucella | 22.6871 | 15 | 14 | Brucella_canis | 0 | 0 | 935/3312761 | 0.0282 | 1 |
| Brucella | 22.6871 | 15 | 14 | Brucella_ceti | 0 | 0 | 759/3278034 | 0.0232 | 1 |
| Brucella | 22.6871 | 15 | 14 | Brucella_melitensis | 0 | 0 | 1002/3311758 | 0.0303 | 1.07 |
| Brucella | 22.6871 | 15 | 14 | Brucella_microti | 0 | 0 | 913/3337369 | 0.0274 | 1 |
| Brucella | 22.6871 | 15 | 14 | Brucella_ovis | 0 | 0 | 408/3275590 | 0.0125 | 1 |
| Brucella | 22.6871 | 15 | 14 | Brucella_pinnipedialis | 0 | 0 | 1097/3399268 | 0.0323 | 1.06 |
| Brucella | 22.6871 | 15 | 14 | Brucella_suis | 0 | 0 | 711/3493289 | 0.0204 | 1 |
| Asticcacaulis | 3.842 | 14 | 13 | Asticcacaulis_excentricus | 14 | 13 | 1622/3904170 | 0.0416 | 1 |
| Pantoea | 3.0759 | 13 | 12 | Pantoea_dispersa | 12 | 12 | 1586/4951455 | 0.032 | 1 |
| Sinorhizobium | 9.7622 | 13 | 12 | Sinorhizobium_meliloti | 5 | 5 | 2515/6813723 | 0.0369 | 1.07 |
| Sinorhizobium | 9.7622 | 13 | 12 | Sinorhizobium_fredii | 4 | 4 | 2905/6476459 | 0.0449 | 1 |
| Agrobacterium | 12.1883 | 12 | 12 | Agrobacterium_fabrum | 3 | 3 | 1777/4917167 | 0.0361 | 1.06 |
| Agrobacterium | 12.1883 | 12 | 12 | Agrobacterium_vitis | 2 | 2 | 1379/5009562 | 0.0275 | 1.03 |
| Agrobacterium | 12.1883 | 12 | 12 | Agrobacterium_H13 | 0 | 0 | 1810/4972229 | 0.0364 | 1.05 |
| Agrobacterium | 12.1883 | 12 | 12 | Agrobacterium_tumefaciens | 0 | 0 | 1536/5481605 | 0.028 | 1.16 |
| Pantoea | 3.0759 | 13 | 12 | Pantoea_vagans | 0 | 0 | 122/4024986 | 0.003 | 1 |
| Sinorhizobium | 9.7622 | 13 | 12 | Sinorhizobium_medicae | 0 | 0 | 928/3781904 | 0.0245 | 1.12 |
| Cutibacterium | 5.1429 | 9 | 9 | Propionibacterium_namnetense | 5 | 5 | 950/2369944 | 0.0401 | 1.15 |
| Micrococcus | 3.2945 | 9 | 9 | Micrococcus_luteus | 4 | 4 | 619/2501097 | 0.0248 | 1 |
| Micrococcus | 3.2945 | 9 | 9 | Micrococcus_terreus | 4 | 4 | 557/3087820 | 0.018 | 1 |
| Cutibacterium | 5.1429 | 9 | 9 | Cutibacterium_granulosum | 3 | 3 | 373/2141325 | 0.0174 | 1 |
| Cutibacterium | 5.1429 | 9 | 9 | Cutibacterium_avidum | 1 | 1 | 113/2729848 | 0.0041 | 1 |
| Rhodopseudomonas | 4.7005 | 8 | 8 | Rhodopseudomonas_palustris | 8 | 8 | 3127/5744041 | 0.0544 | 1 |
| Acidovorax | 4.1205 | 8 | 8 | Acidovorax_KKS102 | 7 | 7 | 1358/5196935 | 0.0261 | 1 |
| Acinetobacter | 2.2793 | 8 | 8 | Acinetobacter_johnsonii | 7 | 7 | 835/3509795 | 0.0238 | 1 |
| Corynebacterium | 3.2693 | 8 | 8 | Corynebacterium_accolens | 2 | 2 | 278/2465976 | 0.0113 | 1 |
| Corynebacterium | 3.2693 | 8 | 8 | Corynebacterium_afermentans | 1 | 1 | 137/2345845 | 0.0058 | 1 |
| Corynebacterium | 3.2693 | 8 | 8 | Corynebacterium_pseudodiphtheriticum | 1 | 1 | 115/2328531 | 0.0049 | 1 |
| Corynebacterium | 3.2693 | 8 | 8 | Corynebacterium_simulans | 1 | 1 | 129/2737971 | 0.0047 | 1 |
| Acidovorax | 4.1205 | 8 | 8 | Acidovorax_JS42 | 0 | 0 | 245/4448856 | 0.0055 | 1 |
| Acidovorax | 4.1205 | 8 | 8 | Acidovorax_citrulli | 0 | 0 | 115/5352772 | 0.0022 | 1 |
| Acidovorax | 4.1205 | 8 | 8 | Acidovorax_ebreus | 0 | 0 | 403/3796573 | 0.0106 | 1 |
| Corynebacterium | 3.2693 | 8 | 8 | Corynebacterium_diphtheriae | 0 | 0 | 146/2535346 | 0.0058 | 1 |
| Corynebacterium | 3.2693 | 8 | 8 | Corynebacterium_tuberculostearicum | 0 | 0 | 216/2372621 | 0.0091 | 1 |
| Xanthobacter | 1.5069 | 7 | 7 | Xanthobacter_autotrophicus | 7 | 7 | 936/5308934 | 0.0176 | 1 |
| Cupriavidus | 2.9012 | 7 | 7 | Cupriavidus_metallidurans | 5 | 5 | 790/3928089 | 0.0201 | 1 |
| Cupriavidus | 2.9012 | 7 | 7 | Cupriavidus_gilardii | 1 | 1 | 215/5578753 | 0.0039 | 1 |
| Cupriavidus | 2.9012 | 7 | 7 | Cupriavidus_necator | 0 | 0 | 135/6557542 | 0.0021 | 1 |
| Cupriavidus | 2.9012 | 7 | 7 | Cupriavidus_pauculus | 0 | 0 | 352/6829059 | 0.0052 | 1 |
| Cupriavidus | 2.9012 | 7 | 7 | Cupriavidus_taiwanensis | 0 | 0 | 85/5919322 | 0.0014 | 1 |
| Sphingobium | 1.9788 | 6 | 6 | Sphingobium_xenophagum | 6 | 6 | 743/4487790 | 0.0166 | 1 |
| Staphylococcus | 2.7197 | 6 | 6 | Staphylococcus_cohnii | 2 | 2 | 248/2677922 | 0.0093 | 1 |
| Staphylococcus | 2.7197 | 6 | 6 | Staphylococcus_warneri | 2 | 2 | 282/2486042 | 0.0113 | 1 |
| Staphylococcus | 2.7197 | 6 | 6 | Staphylococcus_capitis | 1 | 1 | 130/2466594 | 0.0053 | 1 |
| Staphylococcus | 2.7197 | 6 | 6 | Staphylococcus_pasteuri | 1 | 1 | 104/2559946 | 0.0041 | 1 |
| Sphingobium | 1.9788 | 6 | 6 | Sphingobium_japonicum | 0 | 0 | 149/4196714 | 0.0036 | 1 |
| Sphingobium | 1.9788 | 6 | 6 | Sphingobium_yanoikuyae | 0 | 0 | 129/5532659 | 0.0023 | 1 |
| Staphylococcus | 2.7197 | 6 | 6 | Staphylococcus_haemolyticus | 0 | 0 | 122/2685015 | 0.0045 | 1 |
| Herbaspirillum | 1.0882 | 5 | 5 | Herbaspirillum_seropedicae | 5 | 5 | 645/5513887 | 0.0117 | 1 |
| Delftia | 0.9816 | 5 | 5 | Delftia_tsuruhatensis | 4 | 4 | 664/7195716 | 0.0092 | 1 |
| Sphingomonas | 1.8805 | 5 | 5 | Sphingomonas_echinoides | 3 | 3 | 370/4264986 | 0.0087 | 1 |
| Sphingomonas | 1.8805 | 5 | 5 | Sphingomonas_MM | 1 | 1 | 123/4054833 | 0.003 | 1 |
| Delftia | 0.9816 | 5 | 5 | Delftia_acidovorans | 0 | 0 | 117/6767514 | 0.0017 | 1 |
| Sphingomonas | 1.8805 | 5 | 5 | Sphingomonas_melonis | 0 | 0 | 109/4156476 | 0.0026 | 1 |
| Sphingomonas | 1.8805 | 5 | 5 | Sphingomonas_parapaucimobilis | 0 | 0 | 121/3995782 | 0.003 | 1 |
| Sphingomonas | 1.8805 | 5 | 5 | Sphingomonas_paucimobilis | 0 | 0 | 84/4874985 | 0.0017 | 1 |
| Oligotropha | 2.4028 | 4 | 4 | Oligotropha_carboxidovorans | 4 | 4 | 946/3745629 | 0.0253 | 1 |
| Actinomyces | 1.7656 | 4 | 4 | Actinomyces_odontolyticus | 2 | 2 | 222/2432045 | 0.0091 | 1 |
| Actinomyces | 1.7656 | 4 | 4 | Actinomyces_johnsonii | 1 | 1 | 112/3386293 | 0.0033 | 1 |
| Actinomyces | 1.7656 | 4 | 4 | Actinomyces_naeslundii | 0 | 0 | 121/3040449 | 0.004 | 1 |
| Actinomyces | 1.7656 | 4 | 4 | Actinomyces_viscosus | 0 | 0 | 31/3134536 | 0.001 | 1 |
| Afipia | 2.379 | 3 | 3 | Afipia_felis | 3 | 3 | 1113/4203390 | 0.0265 | 1 |
| Haemophilus | 1.4902 | 3 | 3 | Haemophilus_parainfluenzae | 2 | 2 | 268/2086875 | 0.0128 | 1 |
| Nitrobacter | 4.0586 | 3 | 3 | Nitrobacter_hamburgensis | 2 | 2 | 1521/4406967 | 0.0345 | 1 |
| Pseudomonas | 0.6588 | 3 | 3 | Pseudomonas_putida | 2 | 2 | 167/6377271 | 0.0026 | 1 |
| Pseudomonas | 0.6588 | 3 | 3 | Pseudomonas_viridiflava | 1 | 1 | 120/5896435 | 0.002 | 1 |
| Haemophilus | 1.4902 | 3 | 3 | Haemophilus_haemolyticus | 0 | 0 | 121/1880295 | 0.0064 | 1 |
| Nitrobacter | 4.0586 | 3 | 3 | Nitrobacter_winogradskyi | 0 | 0 | 319/3402093 | 0.0094 | 1 |
| Pseudomonas | 0.6588 | 3 | 3 | Pseudomonas_denitrificans | 0 | 0 | 121/5696307 | 0.0021 | 1 |
| Brevundimonas | 1.1825 | 2 | 2 | Brevundimonas_vesicularis | 2 | 2 | 253/3358839 | 0.0075 | 1 |
| Enhydrobacter | 1.7506 | 2 | 2 | Enhydrobacter_aerosaccus | 2 | 2 | 687/2856138 | 0.024 | 1 |
| Massilia | 0.3259 | 2 | 2 | Massilia_timonae | 2 | 2 | 190/6136630 | 0.0031 | 1 |
| Pannonibacter | 1.6921 | 2 | 2 | Pannonibacter_phragmitetus | 2 | 2 | 833/5318696 | 0.0157 | 1.17 |
| Aeromonas | 0.6276 | 2 | 2 | Aeromonas_media | 1 | 1 | 209/4777154 | 0.0044 | 1 |
| Bordetella | 0.7523 | 2 | 2 | Bordetella_bronchialis | 1 | 1 | 170/5966919 | 0.0029 | 1 |
| Bordetella | 0.7523 | 2 | 2 | Bordetella_petrii | 1 | 1 | 130/5287950 | 0.0025 | 1 |
| Enterococcus | 0.6302 | 2 | 2 | Enterococcus_faecium | 1 | 1 | 94/2955294 | 0.0032 | 1 |
| Kocuria | 0.6111 | 2 | 2 | Kocuria_palustris | 1 | 1 | 128/2854447 | 0.0045 | 1 |
| Kocuria | 0.6111 | 2 | 2 | Kocuria_polaris | 1 | 1 | 136/3834128 | 0.0036 | 1 |
| Moraxella | 2.4904 | 2 | 2 | Moraxella_osloensis | 1 | 1 | 469/2434688 | 0.0193 | 1 |
| Neisseria | 0.725 | 2 | 2 | Neisseria_perflava | 1 | 1 | 109/3786149 | 0.0029 | 1 |
| Novosphingobium | 0.7921 | 2 | 2 | Novosphingobium_aromaticivorans | 1 | 1 | 98/3561584 | 0.0028 | 1 |
| Paracoccus | 1.7974 | 2 | 2 | Paracoccus_denitrificans | 1 | 1 | 221/4582379 | 0.0048 | 1 |
| Prevotella | 0.6738 | 2 | 2 | Prevotella_intermedia | 1 | 1 | 146/2699437 | 0.0054 | 1 |
| Streptococcus | 0.903 | 2 | 2 | Streptococcus_downei | 1 | 1 | 155/2239581 | 0.0069 | 1 |
| Aeromonas | 0.6276 | 2 | 2 | Aeromonas_caviae | 0 | 0 | 146/4787529 | 0.003 | 1 |
| Bordetella | 0.7523 | 2 | 2 | Bordetella_pertussis | 0 | 0 | 91/4386396 | 0.0021 | 1 |
| Brevundimonas | 1.1825 | 2 | 2 | Brevundimonas_diminuta | 0 | 0 | 84/3369386 | 0.0025 | 1 |
| Brevundimonas | 1.1825 | 2 | 2 | Brevundimonas_subvibrioides | 0 | 0 | 143/3445263 | 0.0042 | 1 |
| Enterococcus | 0.6302 | 2 | 2 | Enterococcus_casseliflavus | 0 | 0 | 151/3427276 | 0.0044 | 1 |
| Moraxella | 2.4904 | 2 | 2 | Moraxella_atlantae | 0 | 0 | 77/2289665 | 0.0034 | 1 |
| Mycobacterium | 0.3379 | 2 | 2 | Mycobacterium_VKM | 0 | 0 | 103/5438192 | 0.0019 | 1 |
| Mycobacterium | 0.3379 | 2 | 2 | Mycobacterium_vanbaalenii | 0 | 0 | 124/6491865 | 0.0019 | 1 |
| Neisseria | 0.725 | 2 | 2 | Neisseria_mucosa | 0 | 0 | 135/2169497 | 0.0062 | 1 |
| Novosphingobium | 0.7921 | 2 | 2 | Novosphingobium_PP1Y | 0 | 0 | 222/3911486 | 0.0057 | 1 |
| Paracoccus | 1.7974 | 2 | 2 | Paracoccus_aminophilus | 0 | 0 | 192/3613807 | 0.0053 | 1 |
| Paracoccus | 1.7974 | 2 | 2 | Paracoccus_versutus | 0 | 0 | 172/5502608 | 0.0031 | 1 |
| Paracoccus | 1.7974 | 2 | 2 | Paracoccus_yeei | 0 | 0 | 104/4429585 | 0.0023 | 1 |
| Prevotella | 0.6738 | 2 | 2 | Prevotella_scopos | 0 | 0 | 130/3296420 | 0.0039 | 1 |
| Streptococcus | 0.903 | 2 | 2 | Streptococcus_pseudopneumoniae | 0 | 0 | 130/2190731 | 0.0059 | 1 |
| Bifidobacterium | 0.4192 | 1 | 1 | Bifidobacterium_longum | 1 | 1 | 78/2385301 | 0.0033 | 1 |
| Blautia | 0.2217 | 1 | 1 | Blautia_wexlerae | 1 | 1 | 127/4510558 | 0.0028 | 1 |
| Cellulomonas | 0.2932 | 1 | 1 | Cellulomonas_massiliensis | 1 | 1 | 80/3410572 | 0.0023 | 1 |
| Chroococcidiopsis | 0.1583 | 1 | 1 | Chroococcidiopsis_thermalis | 1 | 1 | 105/6315792 | 0.0017 | 1 |
| Comamonas | 0.3722 | 1 | 1 | Comamonas_testosteroni | 1 | 1 | 194/5373644 | 0.0036 | 1 |
| Erwinia | 0.2155 | 1 | 1 | Erwinia_mallotivora | 1 | 1 | 122/4640809 | 0.0026 | 1 |
| Gordonia | 0.2018 | 1 | 1 | Gordonia_sputi | 1 | 1 | 130/4954549 | 0.0026 | 1 |
| Haliangium | 0.1059 | 1 | 1 | Haliangium_ochraceum | 1 | 1 | 101/9446314 | 0.0011 | 1 |
| Inquilinus | 0.8092 | 1 | 1 | Inquilinus_limosus | 1 | 1 | 733/7414344 | 0.0099 | 1 |
| Nitrosomonas | 0.2643 | 1 | 1 | Nitrosomonas_Is79A3 | 1 | 1 | 122/3783444 | 0.0032 | 1 |
| Pandoraea | 0.5104 | 1 | 1 | Pandoraea_sputorum | 1 | 1 | 156/5742997 | 0.0027 | 1 |
| Parvibaculum | 0.5109 | 1 | 1 | Parvibaculum_lavamentivorans | 1 | 1 | 193/3914745 | 0.0049 | 1 |
| Phenylobacterium | 0.2502 | 1 | 1 | Phenylobacterium_zucineum | 1 | 1 | 120/3996255 | 0.003 | 1 |
| Polymorphum | 0.8603 | 1 | 1 | Polymorphum_gilvum | 1 | 1 | 428/4649365 | 0.0092 | 1 |
| Porphyromonas | 0.4758 | 1 | 1 | Porphyromonas_catoniae | 1 | 1 | 88/2101655 | 0.0042 | 1 |
| Psychrobacter | 0.3247 | 1 | 1 | Psychrobacter_G | 1 | 1 | 107/3079438 | 0.0035 | 1 |
| Rhodobacter | 0.2367 | 1 | 1 | Rhodanobacter_2APBS1 | 1 | 1 | 112/4225490 | 0.0027 | 1 |
| Rhodospirillum | 0.4595 | 1 | 1 | Rhodospirillum_rubrum | 1 | 1 | 248/4352825 | 0.0057 | 1.04 |
| Roseomonas | 0.8087 | 1 | 1 | Roseomonas_mucosa | 1 | 1 | 223/4865340 | 0.0046 | 1 |
| Rothia | 0.4416 | 1 | 1 | Rothia_mucilaginosa | 1 | 1 | 113/2264603 | 0.005 | 1 |
| Thermus | 0.4261 | 1 | 1 | Thermus_scotoductus | 1 | 1 | 97/2346803 | 0.0041 | 1 |
| Thiobacillus | 0.3437 | 1 | 1 | Thiobacillus_denitrificans | 1 | 1 | 74/2909809 | 0.0025 | 1 |
| Verminephrobacter | 0.1796 | 1 | 1 | Verminephrobacter_eiseniae | 1 | 1 | 60/5566749 | 0.0011 | 1 |
| Yersinia | 0.208 | 1 | 1 | Yersinia_enterocolitica | 1 | 1 | 142/4807490 | 0.0029 | 1 |
| Bartonella | 3.601 | 1 | 1 | Bartonella_ancashensis | 0 | 0 | 141/1467695 | 0.0096 | 1 |
| Bartonella | 3.601 | 1 | 1 | Bartonella_australis | 0 | 0 | 114/1596490 | 0.0071 | 1 |
| Bartonella | 3.601 | 1 | 1 | Bartonella_quintana | 0 | 0 | 278/1587646 | 0.0175 | 1 |
| Bartonella | 3.601 | 1 | 1 | Bartonella_rochalimae | 0 | 0 | 107/1534163 | 0.007 | 1 |
| Bartonella | 3.601 | 1 | 1 | Bartonella_tribocorum | 0 | 0 | 125/2619061 | 0.0048 | 1 |
| Caulobacter | 0.5477 | 1 | 1 | Caulobacter_K31 | 0 | 0 | 238/5477872 | 0.0043 | 1 |
| Pandoraea | 0.5104 | 1 | 1 | Pandoraea_norimbergensis | 0 | 0 | 64/6167370 | 0.001 | 1 |
| Roseomonas | 0.8087 | 1 | 1 | Roseomonas_gilardii | 0 | 0 | 117/5030491 | 0.0023 | 1.84 |
| Achromobacter | 0.2953 | 0 | 0 | Achromobacter_insuavis | 0 | 0 | 129/6860925 | 0.0019 | 1 |
| Achromobacter | 0.2953 | 0 | 0 | Achromobacter_ruhlandii | 0 | 0 | 104/6687427 | 0.0016 | 1 |
| Acidiphilium | 0.2951 | 0 | 0 | Acidiphilium_cryptum | 0 | 0 | 138/3389227 | 0.0041 | 1 |
| Aureimonas | 2.1474 | 0 | 0 | Aureimonas_altamirensis | 0 | 0 | 988/4191065 | 0.0236 | 1 |
| Azoarcus | 0.1968 | 0 | 0 | Azoarcus_KH32C | 0 | 0 | 115/5081166 | 0.0023 | 1 |
| Azorhizobium | 0.3725 | 0 | 0 | Azorhizobium_caulinodans | 0 | 0 | 231/5369772 | 0.0043 | 1 |
| Azospirillum | 0.3346 | 0 | 0 | Azospirillum_lipoferum | 0 | 0 | 125/2988332 | 0.0042 | 1 |
| Cedecea | 0.2046 | 0 | 0 | Cedecea_davisae | 0 | 0 | 96/4887001 | 0.002 | 1 |
| Cellvibrio | 0.2185 | 0 | 0 | Cellvibrio_japonicus | 0 | 0 | 61/4576573 | 0.0013 | 1 |
| Chelativorans | 0.9065 | 0 | 0 | Chelativorans_BNC1 | 0 | 0 | 458/4412446 | 0.0104 | 1 |
| Dinoroseobacter | 0.5278 | 0 | 0 | Dinoroseobacter_shibae | 0 | 0 | 157/3789584 | 0.0041 | 1 |
| Empedobacter | 0.2691 | 0 | 0 | Empedobacter_falsenii | 0 | 0 | 89/3715858 | 0.0024 | 1 |
| Enterobacter | 0.6147 | 0 | 0 | Enterobacter_cloacae | 0 | 0 | 86/4879702 | 0.0018 | 1 |
| Enterobacter | 0.6147 | 0 | 0 | Enterobacter_kobei | 0 | 0 | 219/4880257 | 0.0045 | 1 |
| Erysipelothrix | 0.5329 | 0 | 0 | Erysipelothrix_rhusiopathiae | 0 | 0 | 138/1876490 | 0.0073 | 1 |
| Flavobacterium | 0.3447 | 0 | 0 | Flavobacterium_psychrophilum | 0 | 0 | 103/2900735 | 0.0036 | 1 |
| Gemmatimonas | 0.2157 | 0 | 0 | Gemmatimonas_aurantiaca | 0 | 0 | 88/4636964 | 0.0019 | 1 |
| Gluconacetobacter | 0.2535 | 0 | 0 | Gluconacetobacter_diazotrophicus | 0 | 0 | 82/3944163 | 0.0021 | 1 |
| Hyphomicrobium | 0.2102 | 0 | 0 | Hyphomicrobium_MC1 | 0 | 0 | 74/4757528 | 0.0016 | 1 |
| Kerstersia | 0.2542 | 0 | 0 | Kerstersia_gyiorum | 0 | 0 | 95/3934293 | 0.0024 | 1 |
| Leifsonia | 0.2361 | 0 | 0 | Leifsonia_aquatica | 0 | 0 | 149/4235539 | 0.0035 | 1 |
| Leisingera | 0.4825 | 0 | 0 | Leisingera_methylohalidivorans | 0 | 0 | 194/4144900 | 0.0047 | 1 |
| Leptothrix | 0.2037 | 0 | 0 | Leptothrix_cholodnii | 0 | 0 | 74/4909403 | 0.0015 | 1 |
| Magnetospirillum | 0.8885 | 0 | 0 | Magnetospirillum_gryphiswaldense | 0 | 0 | 234/4365796 | 0.0054 | 1.54 |
| Magnetospirillum | 0.8885 | 0 | 0 | Magnetospirillum_magneticum | 0 | 0 | 43/4967148 | 0.0009 | 1 |
| Methylibium | 0.4945 | 0 | 0 | Methylibium_petroleiphilum | 0 | 0 | 187/4044195 | 0.0046 | 1 |
| Methylobacillus | 0.3365 | 0 | 0 | Methylobacillus_flagellatus | 0 | 0 | 144/2971517 | 0.0049 | 1 |
| - | - | 0 | - | Methylocystis_SC2 | 0 | 0 | 311/3773444 | 0.0082 | 1 |
| Microbacterium | 0.221 | 0 | 0 | Microbacterium_trichothecenolyticum | 0 | 0 | 71/4524680 | 0.0016 | 1 |
| Octadecabacter | 0.1923 | 0 | 0 | Octadecabacter_arcticus | 0 | 0 | 119/5200279 | 0.0023 | 1 |
| Ramlibacter | 0.2457 | 0 | 0 | Ramlibacter_tataouinensis | 0 | 0 | 59/4070193 | 0.0014 | 1 |
| Sphingopyxis | 0.2989 | 0 | 0 | Sphingopyxis_alaskensis | 0 | 0 | 146/3345170 | 0.0044 | 1 |
| Starkeya | 0.4197 | 0 | 0 | Starkeya_novella | 0 | 0 | 166/4765023 | 0.0035 | 1 |
| Stenotrophomonas | 0.2217 | 0 | 0 | Stenotrophomonas_maltophilia | 0 | 0 | 126/4509724 | 0.0028 | 1 |
| Thauera | 0.2224 | 0 | 0 | Thauera_MZ1T | 0 | 0 | 123/4496212 | 0.0027 | 1 |
| Tistrella | 0.5103 | 0 | 0 | Tistrella_mobilis | 0 | 0 | 256/3919492 | 0.0065 | 1 |
| Variovorax | 0.1399 | 0 | 0 | Variovorax_paradoxus | 0 | 0 | 41/7148516 | 0.0006 | 1 |
| Candidatus_Liberibacter | 0.6646 | 0 | 0 | bacterium_BT | 0 | 0 | 95/1504659 | 0.0063 | 1 |

Note: Genus Re Abu: Relative abundance of Genus; SMRNG: Stringent mapped reads number of genus; SDSMRNG: Standard Stringent mapped reads number of genus; SMRN: Stringent mapped reads number; SDSMRN: Standard Stringent mapped reads number; CovRate: Coverage rate.

　　 Table 2. Microbe reads of fungi, parasite and virus detected in Case No. 1

| Genus | Genus Abs Abu | SMRNG | SDSMRNG | Species | SMRN | SDSMRN | Coverage | CovRate | Depth |
| --- | --- | --- | --- | --- | --- | --- | --- | --- | --- |
| Malassezia | 0.3381 | 3 | 3 | Malassezia_globosa | 3 | 3 | 380/8872979 | 0.0043 | 1 |
| Aspergillus | 0.0302 | 1 | 1 | Aspergillus_versicolor | 1 | 1 | 138/33127310 | 0.0004 | 1 |
| Phanerochaete | 0.0335 | 1 | 1 | Phanerochaete_chrysosporium | 1 | 1 | 96/29855776 | 0.0003 | 1 |
| Purpureocillium | 0.0259 | 1 | 1 | Purpureocillium_lilacinus | 1 | 1 | 106/38536221 | 0.0003 | 1 |
| Sordaria | 0.5 | 1 | 1 | Sordaria_macrospora | 1 | 1 | 1488/40002837 | 0.0037 | 1.58 |
| Saccharomyces | 0.0842 | 0 | 0 | Saccharomyces_paradoxus | 0 | 0 | 39/11880927 | 0.0003 | 1 |
| Schizosaccharomyces | 0.086 | 0 | 0 | Schizosaccharomyces_octosporus | 0 | 0 | 41/11634513 | 0.0003 | 1 |
| Trypanosoma | 1.0734 | 4 | 4 | Trypanosoma_cruzi | 4 | 4 | 290/81175611 | 0.0004 | 1.64 |
| Acanthamoeba | 3.2417 | 61 | 58 | Acanthamoeba_mauritaniensis | 3 | 3 | 4563/96417684 | 0.0047 | 1.07 |
| Hammondia | 0.0492 | 3 | 3 | Hammondia_hammondi | 3 | 3 | 361/60951907 | 0.0006 | 1 |
| Acanthamoeba | 3.2417 | 61 | 58 | Acanthamoeba_lenticulata | 1 | 1 | 110/59662351 | 0.0002 | 1 |
| Acanthamoeba | 3.2417 | 61 | 58 | Acanthamoeba_palestinensis | 0 | 0 | 14690/93383381 | 0.0157 | 1.04 |
| Acanthamoeba | 3.2417 | 61 | 58 | Acanthamoeba_triangularis | 0 | 0 | 13173/85627420 | 0.0154 | 1.01 |
| Anisakis | 0.0087 | 0 | 0 | Anisakis_simplex | 0 | 0 | 109/114426609 | 0.0001 | 1 |
| Chromera | 0.0573 | 0 | 0 | Chromera_velia | 0 | 0 | 738/174539467 | 0.0004 | 1.65 |
| Entamoeba | 0.1493 | 0 | 0 | Entamoeba_dispar | 0 | 0 | 175/27672952 | 0.0006 | 1 |
| Entamoeba | 0.1493 | 0 | 0 | Entamoeba_nuttalli | 0 | 0 | 91/12984033 | 0.0007 | 1 |
| - | - | - | - | Human alphaherpesvirus 3 (Varicella zoster virus) | 17879 | 17137 | 124544/124884 | 99.73 | 17.9 |

Note: Genus Re Abu: Relative abundance of Genus; SMRNG: Stringent mapped reads number of genus; SDSMRNG: Standard Stringent mapped reads number of genus; SMRN: Stringent mapped reads number; SDSMRN: Standard Stringent mapped reads number; CovRate: Coverage rate.
