# Supplementary material for: Clinical features of aseptic meningitis with varicella zoster virus infection diagnosed by next-generation sequencing: case reports
Source: BMC Infect Dis. 2020 Jun 22;20:435. doi: 10.1186/s12879-020-05155-8 (PMC7309994; doi:10.1186/s12879-020-05155-8)
Supplement: Supplementary file 3 — Additional file 3. Microbe reads of bacterium, fungi, parasite and virus detected in Case No. 2 [file 12879_2020_5155_MOESM3_ESM.docx]

**Additional file 3:** Microbe reads of bacterium, fungi, parasite and virus detected in Case No. 2

　Table 1. Microbe reads of bacterium detected in Case No. 2

| Genus | Genus Abs Abu | SMRNG | SDSMRNG | Species | SMRN | SDSMRN | Coverage | CovRate | Depth |
| --- | --- | --- | --- | --- | --- | --- | --- | --- | --- |
| Ralstonia | 3428.2334 | 6779 | 3028 | Ralstonia_mannitolilytica | 2449 | 1094 | 357977/4881769 | 7.33 | 1.2 |
| Ralstonia | 3428.2334 | 6779 | 3028 | Ralstonia_insidiosa | 1249 | 558 | 280588/5808308 | 4.83 | 1.17 |
| Ralstonia | 3428.2334 | 6779 | 3028 | Ralstonia_solanacearum | 173 | 77 | 84277/3984240 | 2.12 | 1.13 |
| Ralstonia | 3428.2334 | 6779 | 3028 | Ralstonia_pickettii | 4 | 2 | 7994/8125850 | 0.0984 | 1.03 |
| Delftia | 824.0061 | 4897 | 2188 | Delftia_tsuruhatensis | 1489 | 665 | 187030/7195716 | 2.6 | 1.02 |
| Delftia | 824.0061 | 4897 | 2188 | Delftia_acidovorans | 108 | 48 | 50301/6767514 | 0.7433 | 1.01 |
| Delftia | 824.0061 | 4897 | 2188 | Delftia_Cs1 | 54 | 24 | 46398/6685842 | 0.694 | 1.01 |
| Burkholderia | 1378.6079 | 3988 | 1782 | Burkholderia_ubonensis | 136 | 61 | 73485/8028779 | 0.9153 | 1.05 |
| Burkholderia | 1378.6079 | 3988 | 1782 | Burkholderia_stabilis | 32 | 14 | 21563/8527967 | 0.2529 | 1.05 |
| Burkholderia | 1378.6079 | 3988 | 1782 | Burkholderia_multivorans | 26 | 12 | 31901/7281887 | 0.4381 | 1.04 |
| Burkholderia | 1378.6079 | 3988 | 1782 | Burkholderia_dolosa | 15 | 7 | 25900/6409115 | 0.4041 | 1.03 |
| Burkholderia | 1378.6079 | 3988 | 1782 | Burkholderia_glumae | 15 | 7 | 9523/6733840 | 0.1414 | 1.07 |
| Burkholderia | 1378.6079 | 3988 | 1782 | Burkholderia_ambifaria | 13 | 6 | 20089/7484986 | 0.2684 | 1.04 |
| Burkholderia | 1378.6079 | 3988 | 1782 | Burkholderia_oklahomensis | 14 | 6 | 17645/7313683 | 0.2413 | 1.06 |
| Burkholderia | 1378.6079 | 3988 | 1782 | Burkholderia_cenocepacia | 11 | 5 | 20973/8531679 | 0.2458 | 1.03 |
| Burkholderia | 1378.6079 | 3988 | 1782 | Burkholderia_latens | 12 | 5 | 18846/6569017 | 0.2869 | 1.03 |
| Burkholderia | 1378.6079 | 3988 | 1782 | Burkholderia_cepacia | 10 | 4 | 20556/8396158 | 0.2448 | 1.04 |
| Burkholderia | 1378.6079 | 3988 | 1782 | Burkholderia_diffusa | 7 | 3 | 22336/6857853 | 0.3257 | 1.04 |
| Burkholderia | 1378.6079 | 3988 | 1782 | Burkholderia_metallica | 6 | 3 | 17699/7424260 | 0.2384 | 1.02 |
| Burkholderia | 1378.6079 | 3988 | 1782 | Burkholderia_pyrrocinia | 6 | 3 | 21931/7847882 | 0.2795 | 1.04 |
| Burkholderia | 1378.6079 | 3988 | 1782 | Burkholderia_thailandensis | 6 | 3 | 14190/6739510 | 0.2105 | 1.03 |
| Burkholderia | 1378.6079 | 3988 | 1782 | Burkholderia_anthina | 5 | 2 | 19909/7273081 | 0.2737 | 1.03 |
| Burkholderia | 1378.6079 | 3988 | 1782 | Burkholderia_contaminans | 4 | 2 | 16912/8509269 | 0.1987 | 1.03 |
| Burkholderia | 1378.6079 | 3988 | 1782 | Burkholderia_gladioli | 5 | 2 | 9949/8114449 | 0.1226 | 1.05 |
| Burkholderia | 1378.6079 | 3988 | 1782 | Burkholderia_rhizoxinica | 4 | 2 | 866/2755309 | 0.0314 | 1 |
| Burkholderia | 1378.6079 | 3988 | 1782 | Burkholderia_CCGE1002 | 2 | 1 | 5581/7395722 | 0.0755 | 1.04 |
| Burkholderia | 1378.6079 | 3988 | 1782 | Burkholderia_CCGE1003 | 1 | 1 | 3220/7043595 | 0.0457 | 1.01 |
| Burkholderia | 1378.6079 | 3988 | 1782 | Burkholderia_KJ006 | 2 | 1 | 14745/6584551 | 0.2239 | 1.01 |
| Burkholderia | 1378.6079 | 3988 | 1782 | Burkholderia_lata | 2 | 1 | 15566/8676277 | 0.1794 | 1.04 |
| Burkholderia | 1378.6079 | 3988 | 1782 | Burkholderia_phymatum | 2 | 1 | 4700/6176561 | 0.0761 | 1.1 |
| Burkholderia | 1378.6079 | 3988 | 1782 | Burkholderia_phytofirmans | 3 | 1 | 3969/8093536 | 0.049 | 1.02 |
| Burkholderia | 1378.6079 | 3988 | 1782 | Burkholderia_pseudomallei | 1 | 1 | 9380/7446579 | 0.126 | 1.03 |
| Burkholderia | 1378.6079 | 3988 | 1782 | Burkholderia_RPE64 | 2 | 1 | 3176/5379596 | 0.059 | 1.04 |
| Burkholderia | 1378.6079 | 3988 | 1782 | Burkholderia_xenovorans | 3 | 1 | 4199/9731138 | 0.0432 | 1.05 |
| Burkholderia | 1378.6079 | 3988 | 1782 | Burkholderia_YI23 | 3 | 1 | 3764/6473869 | 0.0581 | 1.06 |
| Burkholderia | 1378.6079 | 3988 | 1782 | Burkholderia_CCGE1001 | 0 | 0 | 2782/6833751 | 0.0407 | 1.02 |
| Burkholderia | 1378.6079 | 3988 | 1782 | Burkholderia_mallei | 0 | 0 | 8704/5913144 | 0.1472 | 1.02 |
| Burkholderia | 1378.6079 | 3988 | 1782 | Burkholderia_phenoliruptrix | 0 | 0 | 3055/6865712 | 0.0445 | 1.04 |
| Burkholderia | 1378.6079 | 3988 | 1782 | Burkholderia_vietnamiensis | 0 | 0 | 14657/6827896 | 0.2147 | 1.03 |
| Sphingomonas | 986.861 | 1683 | 752 | Sphingomonas_parapaucimobilis | 1065 | 476 | 124698/3995782 | 3.12 | 1.05 |
| Sphingomonas | 986.861 | 1683 | 752 | Sphingomonas_echinoides | 332 | 148 | 34087/4264986 | 0.7992 | 1.02 |
| Sphingomonas | 986.861 | 1683 | 752 | Sphingomonas_melonis | 126 | 56 | 18351/4156476 | 0.4415 | 1.06 |
| Sphingomonas | 986.861 | 1683 | 752 | Sphingomonas_adhaesiva | 25 | 11 | 8384/4026130 | 0.2082 | 1.05 |
| Sphingomonas | 986.861 | 1683 | 752 | Sphingomonas_MM | 1 | 1 | 1528/4054833 | 0.0377 | 1 |
| Sphingomonas | 986.861 | 1683 | 752 | Sphingomonas_paucimobilis | 0 | 0 | 1102/4874985 | 0.0226 | 1 |
| Sphingomonas | 986.861 | 1683 | 752 | Sphingomonas_wittichii | 0 | 0 | 1740/5382261 | 0.0323 | 1 |
| Staphylococcus | 237.7161 | 437 | 195 | Staphylococcus_epidermidis | 147 | 66 | 8400/2616530 | 0.321 | 1 |
| Staphylococcus | 237.7161 | 437 | 195 | Staphylococcus_hominis | 87 | 39 | 4774/1140916 | 0.4184 | 1 |
| Staphylococcus | 237.7161 | 437 | 195 | Staphylococcus_haemolyticus | 51 | 23 | 3182/2685015 | 0.1185 | 1 |
| Staphylococcus | 237.7161 | 437 | 195 | Staphylococcus_pasteuri | 31 | 14 | 3748/2559946 | 0.1464 | 1 |
| Staphylococcus | 237.7161 | 437 | 195 | Staphylococcus_warneri | 14 | 6 | 2587/2486042 | 0.1041 | 1 |
| Staphylococcus | 237.7161 | 437 | 195 | Staphylococcus_auricularis | 5 | 2 | 400/2202238 | 0.0182 | 1 |
| Staphylococcus | 237.7161 | 437 | 195 | Staphylococcus_capitis | 5 | 2 | 450/2466594 | 0.0182 | 1 |
| Staphylococcus | 237.7161 | 437 | 195 | Staphylococcus_pettenkoferi | 4 | 2 | 200/2502346 | 0.008 | 1 |
| Staphylococcus | 237.7161 | 437 | 195 | Staphylococcus_caprae | 1 | 1 | 200/2629820 | 0.0076 | 1 |
| Staphylococcus | 237.7161 | 437 | 195 | Staphylococcus_cohnii | 2 | 1 | 100/2677922 | 0.0037 | 1 |
| Staphylococcus | 237.7161 | 437 | 195 | Staphylococcus_equorum | 1 | 1 | 100/2753539 | 0.0036 | 1 |
| Staphylococcus | 237.7161 | 437 | 195 | Staphylococcus_saprophyticus | 1 | 1 | 92/2516575 | 0.0037 | 1 |
| Staphylococcus | 237.7161 | 437 | 195 | Staphylococcus_aureus | 0 | 0 | 50/2898306 | 0.0017 | 1 |
| Staphylococcus | 237.7161 | 437 | 195 | Staphylococcus_delphini | 0 | 0 | 51/2850609 | 0.0018 | 1 |
| Staphylococcus | 237.7161 | 437 | 195 | Staphylococcus_lutrae | 0 | 0 | 50/2533115 | 0.002 | 1 |
| Staphylococcus | 237.7161 | 437 | 195 | Staphylococcus_massiliensis | 0 | 0 | 50/2366665 | 0.0021 | 1 |
| Staphylococcus | 237.7161 | 437 | 195 | Staphylococcus_saccharolyticus | 0 | 0 | 100/2661041 | 0.0038 | 1 |
| Propionibacterium | 207.1374 | 415 | 185 | Propionibacterium_acnes | 374 | 167 | 25674/2560282 | 1 | 1 |
| Propionibacterium | 207.1374 | 415 | 185 | Propionibacterium_humerusii | 7 | 3 | 646/2644116 | 0.0244 | 1 |
| Propionibacterium | 207.1374 | 415 | 185 | Propionibacterium_propionicum | 1 | 1 | 50/3449360 | 0.0014 | 1 |
| Alicycliphilus | 145.9383 | 397 | 177 | Alicycliphilus_denitrificans | 397 | 177 | 29018/4995263 | 0.5809 | 1.25 |
| Moraxella | 110.1289 | 192 | 86 | Moraxella_osloensis | 186 | 83 | 13128/2434688 | 0.5392 | 1 |
| Moraxella | 110.1289 | 192 | 86 | Moraxella_atlantae | 1 | 1 | 100/2289665 | 0.0044 | 1 |
| Moraxella | 110.1289 | 192 | 86 | Moraxella_boevrei | 0 | 0 | 50/2427634 | 0.0021 | 1 |
| Acinetobacter | 67.5841 | 172 | 77 | Acinetobacter_johnsonii | 77 | 34 | 5357/3509795 | 0.1526 | 1 |
| Acinetobacter | 67.5841 | 172 | 77 | Acinetobacter_parvus | 26 | 12 | 3328/3606729 | 0.0923 | 1 |
| Acinetobacter | 67.5841 | 172 | 77 | Acinetobacter_soli | 7 | 3 | 350/3463526 | 0.0101 | 1 |
| Acinetobacter | 67.5841 | 172 | 77 | Acinetobacter_guillouiae | 5 | 2 | 250/4905802 | 0.0051 | 1 |
| Acinetobacter | 67.5841 | 172 | 77 | Acinetobacter_junii | 4 | 2 | 296/3782588 | 0.0078 | 1 |
| Acinetobacter | 67.5841 | 172 | 77 | Acinetobacter_baumannii | 2 | 1 | 450/4086879 | 0.011 | 1 |
| Acinetobacter | 67.5841 | 172 | 77 | Acinetobacter_beijerinckii | 1 | 1 | 100/3563374 | 0.0028 | 1 |
| Acinetobacter | 67.5841 | 172 | 77 | Acinetobacter_bereziniae | 1 | 1 | 150/5026552 | 0.003 | 1 |
| Acinetobacter | 67.5841 | 172 | 77 | Acinetobacter_haemolyticus | 3 | 1 | 150/3715798 | 0.004 | 1 |
| Acinetobacter | 67.5841 | 172 | 77 | Acinetobacter_indicus | 1 | 1 | 50/3157380 | 0.0016 | 1 |
| Acinetobacter | 67.5841 | 172 | 77 | Acinetobacter_schindleri | 2 | 1 | 101/3415006 | 0.003 | 1 |
| Acinetobacter | 67.5841 | 172 | 77 | Acinetobacter_ursingii | 2 | 1 | 200/3825524 | 0.0052 | 1 |
| Acinetobacter | 67.5841 | 172 | 77 | Acinetobacter_bohemicus | 0 | 0 | 150/3729387 | 0.004 | 1 |
| Acinetobacter | 67.5841 | 172 | 77 | Acinetobacter_bouvetii | 0 | 0 | 150/3388872 | 0.0044 | 1 |
| Acinetobacter | 67.5841 | 172 | 77 | Acinetobacter_calcoaceticus | 0 | 0 | 50/3862530 | 0.0013 | 1 |
| Acinetobacter | 67.5841 | 172 | 77 | Acinetobacter_dijkshoorniae | 0 | 0 | 200/3859369 | 0.0052 | 1 |
| Acinetobacter | 67.5841 | 172 | 77 | Acinetobacter_harbinensis | 0 | 0 | 50/2899835 | 0.0017 | 1 |
| Acinetobacter | 67.5841 | 172 | 77 | Acinetobacter_kookii | 0 | 0 | 142/3085611 | 0.0046 | 1 |
| Acinetobacter | 67.5841 | 172 | 77 | Acinetobacter_lwoffii | 0 | 0 | 145/2970206 | 0.0049 | 1 |
| Acinetobacter | 67.5841 | 172 | 77 | Acinetobacter_nosocomialis | 0 | 0 | 50/3858956 | 0.0013 | 1 |
| Acinetobacter | 67.5841 | 172 | 77 | Acinetobacter_oleivorans | 0 | 0 | 150/4152543 | 0.0036 | 1 |
| Acinetobacter | 67.5841 | 172 | 77 | Acinetobacter_pittii | 0 | 0 | 150/4033345 | 0.0037 | 1 |
| Acinetobacter | 67.5841 | 172 | 77 | Acinetobacter_proteolyticus | 0 | 0 | 100/4375000 | 0.0023 | 1 |
| Brevundimonas | 76.4108 | 169 | 75 | Brevundimonas_vesicularis | 150 | 67 | 11394/3358839 | 0.3392 | 1 |
| Brevundimonas | 76.4108 | 169 | 75 | Brevundimonas_diminuta | 1 | 1 | 737/3369386 | 0.0219 | 1 |
| Brevundimonas | 76.4108 | 169 | 75 | Brevundimonas_subvibrioides | 0 | 0 | 590/3445263 | 0.0171 | 1 |
| Corynebacterium | 75.1339 | 153 | 68 | Corynebacterium_callunae | 71 | 32 | 3873/2839551 | 0.1364 | 1 |
| Corynebacterium | 75.1339 | 153 | 68 | Corynebacterium_accolens | 17 | 8 | 1349/2465976 | 0.0547 | 1 |
| Corynebacterium | 75.1339 | 153 | 68 | Corynebacterium_lowii | 5 | 2 | 250/2354533 | 0.0106 | 1 |
| Corynebacterium | 75.1339 | 153 | 68 | Corynebacterium_matruchotii | 4 | 2 | 300/2992345 | 0.01 | 1 |
| Corynebacterium | 75.1339 | 153 | 68 | Corynebacterium_tuberculostearicum | 5 | 2 | 1288/2372621 | 0.0543 | 1 |
| Corynebacterium | 75.1339 | 153 | 68 | Corynebacterium_casei | 2 | 1 | 100/3113488 | 0.0032 | 1 |
| Corynebacterium | 75.1339 | 153 | 68 | Corynebacterium_glutamicum | 1 | 1 | 50/3342897 | 0.0015 | 1 |
| Corynebacterium | 75.1339 | 153 | 68 | Corynebacterium_ihumii | 1 | 1 | 150/2251322 | 0.0067 | 1 |
| Corynebacterium | 75.1339 | 153 | 68 | Corynebacterium_kroppenstedtii | 2 | 1 | 198/2446804 | 0.0081 | 1 |
| Corynebacterium | 75.1339 | 153 | 68 | Corynebacterium_lipophiloflavum | 1 | 1 | 50/2386544 | 0.0021 | 1 |
| Corynebacterium | 75.1339 | 153 | 68 | Corynebacterium_pseudogenitalium | 1 | 1 | 742/2601506 | 0.0285 | 1 |
| Corynebacterium | 75.1339 | 153 | 68 | Corynebacterium_simulans | 2 | 1 | 100/2737971 | 0.0037 | 1 |
| Corynebacterium | 75.1339 | 153 | 68 | Corynebacterium_singulare | 1 | 1 | 50/2830519 | 0.0018 | 1 |
| Corynebacterium | 75.1339 | 153 | 68 | Corynebacterium_ureicelerivorans | 2 | 1 | 100/2279990 | 0.0044 | 1 |
| Corynebacterium | 75.1339 | 153 | 68 | Corynebacterium_xerosis | 1 | 1 | 50/2751422 | 0.0018 | 1 |
| Corynebacterium | 75.1339 | 153 | 68 | Corynebacterium_afermentans | 0 | 0 | 250/2345845 | 0.0107 | 1 |
| Corynebacterium | 75.1339 | 153 | 68 | Corynebacterium_aurimucosum | 0 | 0 | 45/2790189 | 0.0016 | 1 |
| Corynebacterium | 75.1339 | 153 | 68 | Corynebacterium_bovis | 0 | 0 | 66/2527982 | 0.0026 | 1.52 |
| Corynebacterium | 75.1339 | 153 | 68 | Corynebacterium_capitovis | 0 | 0 | 50/1960906 | 0.0025 | 1 |
| Corynebacterium | 75.1339 | 153 | 68 | Corynebacterium_durum | 0 | 0 | 45/2809766 | 0.0016 | 1 |
| Corynebacterium | 75.1339 | 153 | 68 | Corynebacterium_efficiens | 0 | 0 | 50/3147090 | 0.0016 | 1 |
| Corynebacterium | 75.1339 | 153 | 68 | Corynebacterium_flavescens | 0 | 0 | 50/2758653 | 0.0018 | 1 |
| Corynebacterium | 75.1339 | 153 | 68 | Corynebacterium_humireducens | 0 | 0 | 41/2681312 | 0.0015 | 1 |
| Corynebacterium | 75.1339 | 153 | 68 | Corynebacterium_imitans | 0 | 0 | 50/2565321 | 0.0019 | 1 |
| Corynebacterium | 75.1339 | 153 | 68 | Corynebacterium_jeikeium | 0 | 0 | 95/2462499 | 0.0039 | 1 |
| Corynebacterium | 75.1339 | 153 | 68 | Corynebacterium_maris | 0 | 0 | 42/2787574 | 0.0015 | 1 |
| Corynebacterium | 75.1339 | 153 | 68 | Corynebacterium_mastitidis | 0 | 0 | 46/2370385 | 0.0019 | 1 |
| Corynebacterium | 75.1339 | 153 | 68 | Corynebacterium_mycetoides | 0 | 0 | 100/2266470 | 0.0044 | 1 |
| Corynebacterium | 75.1339 | 153 | 68 | Corynebacterium_oculi | 0 | 0 | 44/2413903 | 0.0018 | 1 |
| Corynebacterium | 75.1339 | 153 | 68 | Corynebacterium_riegelii | 0 | 0 | 47/2563723 | 0.0018 | 1 |
| Corynebacterium | 75.1339 | 153 | 68 | Corynebacterium_vitaeruminis | 0 | 0 | 50/2931780 | 0.0017 | 1 |
| Aquabacterium | 83.7106 | 150 | 67 | Aquabacterium_parvum | 150 | 67 | 18256/4599179 | 0.3969 | 1.04 |
| Empedobacter | 60.2822 | 140 | 63 | Empedobacter_falsenii | 140 | 63 | 8511/3715858 | 0.229 | 1.31 |
| Methylobacterium | 33.0942 | 115 | 51 | Methylobacterium_chloromethanicum | 11 | 5 | 3373/5777908 | 0.0584 | 1 |
| Methylobacterium | 33.0942 | 115 | 51 | Methylobacterium_mesophilicum | 4 | 2 | 766/6214729 | 0.0123 | 1 |
| Methylobacterium | 33.0942 | 115 | 51 | Methylobacterium_radiotolerans | 4 | 2 | 1220/6077833 | 0.0201 | 1 |
| Methylobacterium | 33.0942 | 115 | 51 | Methylobacterium_aquaticum | 2 | 1 | 272/5348274 | 0.0051 | 1 |
| Methylobacterium | 33.0942 | 115 | 51 | Methylobacterium_brachiatum | 3 | 1 | 927/5807713 | 0.016 | 1 |
| Methylobacterium | 33.0942 | 115 | 51 | Methylobacterium_extorquens | 3 | 1 | 2280/5943768 | 0.0384 | 1 |
| Methylobacterium | 33.0942 | 115 | 51 | Methylobacterium_populi | 2 | 1 | 348/5800441 | 0.006 | 1 |
| Methylobacterium | 33.0942 | 115 | 51 | Methylobacterium_4 | 0 | 0 | 296/7659055 | 0.0039 | 1 |
| Methylobacterium | 33.0942 | 115 | 51 | Methylobacterium_nodulans | 0 | 0 | 200/7772460 | 0.0026 | 1 |
| Neisseria | 91.1351 | 82 | 37 | Neisseria_perflava | 42 | 19 | 12186/3786149 | 0.3219 | 1.1 |
| Neisseria | 91.1351 | 82 | 37 | Neisseria_elongata | 2 | 1 | 200/2256647 | 0.0089 | 1 |
| Neisseria | 91.1351 | 82 | 37 | Neisseria_macacae | 2 | 1 | 1033/2749018 | 0.0376 | 1 |
| Neisseria | 91.1351 | 82 | 37 | Neisseria_meningitidis | 1 | 1 | 100/2179483 | 0.0046 | 1 |
| Neisseria | 91.1351 | 82 | 37 | Neisseria_sicca | 3 | 1 | 601/2831372 | 0.0212 | 1 |
| Neisseria | 91.1351 | 82 | 37 | Neisseria_subflava | 1 | 1 | 150/2293606 | 0.0065 | 1 |
| Neisseria | 91.1351 | 82 | 37 | Neisseria_canis | 0 | 0 | 50/2530349 | 0.002 | 1 |
| Neisseria | 91.1351 | 82 | 37 | Neisseria_flavescens | 0 | 0 | 100/2345744 | 0.0043 | 1 |
| Neisseria | 91.1351 | 82 | 37 | Neisseria_gonorrhoeae | 0 | 0 | 50/2233640 | 0.0022 | 1 |
| Neisseria | 91.1351 | 82 | 37 | Neisseria_lactamica | 0 | 0 | 244/2220606 | 0.011 | 1 |
| Mitsuaria | 109.4509 | 77 | 34 | Mitsuaria_chitosanitabida | 77 | 34 | 28596/5819963 | 0.4913 | 1.08 |
| Acidovorax | 80.4366 | 70 | 31 | Acidovorax_KKS102 | 59 | 26 | 8314/5196935 | 0.16 | 1.02 |
| Acidovorax | 80.4366 | 70 | 31 | Acidovorax_JS42 | 4 | 2 | 2985/4448856 | 0.0671 | 1.02 |
| Acidovorax | 80.4366 | 70 | 31 | Acidovorax_avenae | 0 | 0 | 2822/5482170 | 0.0515 | 1.02 |
| Acidovorax | 80.4366 | 70 | 31 | Acidovorax_citrulli | 0 | 0 | 3249/5352772 | 0.0607 | 1.01 |
| Acidovorax | 80.4366 | 70 | 31 | Acidovorax_ebreus | 0 | 0 | 1813/3796573 | 0.0478 | 1 |
| Bradyrhizobium | 27.3114 | 50 | 22 | Bradyrhizobium_BTAi1 | 25 | 11 | 1979/8264687 | 0.0239 | 1.01 |
| Bradyrhizobium | 27.3114 | 50 | 22 | Bradyrhizobium_S23321 | 10 | 4 | 3804/7231841 | 0.0526 | 1 |
| Bradyrhizobium | 27.3114 | 50 | 22 | Bradyrhizobium_japonicum | 3 | 1 | 2833/9207384 | 0.0308 | 1 |
| Bradyrhizobium | 27.3114 | 50 | 22 | Bradyrhizobium_oligotrophicum | 0 | 0 | 1356/8264165 | 0.0164 | 1 |
| Bradyrhizobium | 27.3114 | 50 | 22 | Bradyrhizobium_ORS | 0 | 0 | 679/7456587 | 0.0091 | 1 |
| Cupriavidus | 186.308 | 50 | 22 | Cupriavidus_gilardii | 8 | 4 | 10787/5578753 | 0.1934 | 1.04 |
| Cupriavidus | 186.308 | 50 | 22 | Cupriavidus_necator | 6 | 3 | 8023/6557542 | 0.1223 | 1.06 |
| Cupriavidus | 186.308 | 50 | 22 | Cupriavidus_nantongensis | 4 | 2 | 8530/7075719 | 0.1206 | 1.04 |
| Cupriavidus | 186.308 | 50 | 22 | Cupriavidus_pauculus | 5 | 2 | 8249/6829059 | 0.1208 | 1.06 |
| Cupriavidus | 186.308 | 50 | 22 | Cupriavidus_taiwanensis | 4 | 2 | 6537/5919322 | 0.1104 | 1.07 |
| Cupriavidus | 186.308 | 50 | 22 | Cupriavidus_metallidurans | 3 | 1 | 7606/3928089 | 0.1936 | 1.08 |
| Xanthomonas | 13.5601 | 42 | 19 | Xanthomonas_campestris | 36 | 16 | 2616/4908383 | 0.0533 | 1 |
| Xanthomonas | 13.5601 | 42 | 19 | Xanthomonas_albilineans | 0 | 0 | 100/3768695 | 0.0027 | 1 |
| Xanthomonas | 13.5601 | 42 | 19 | Xanthomonas_citri | 0 | 0 | 89/5175552 | 0.0017 | 1 |
| Xanthomonas | 13.5601 | 42 | 19 | Xanthomonas_oryzae | 0 | 0 | 135/5238555 | 0.0026 | 1 |
| Xanthomonas | 13.5601 | 42 | 19 | Xanthomonas_translucens | 0 | 0 | 286/4715357 | 0.0061 | 1 |
| Kocuria | 13.4455 | 30 | 13 | Kocuria_palustris | 23 | 10 | 1300/2854447 | 0.0455 | 1 |
| Kocuria | 13.4455 | 30 | 13 | Kocuria_polaris | 4 | 2 | 292/3834128 | 0.0076 | 1 |
| Kocuria | 13.4455 | 30 | 13 | Kocuria_kristinae | 3 | 1 | 200/2409808 | 0.0083 | 1 |
| Kocuria | 13.4455 | 30 | 13 | Kocuria_rhizophila | 0 | 0 | 145/2697540 | 0.0054 | 1 |
| Aquincola | 45.5659 | 24 | 11 | Aquincola_tertiaricarbonis | 24 | 11 | 13126/6320516 | 0.2077 | 1.06 |
| Asticcacaulis | 10.8712 | 22 | 10 | Asticcacaulis_excentricus | 21 | 9 | 2038/3904170 | 0.0522 | 1 |
| Asticcacaulis | 10.8712 | 22 | 10 | Asticcacaulis_biprosthecium | 0 | 0 | 95/5411575 | 0.0018 | 1 |
| Pseudomonas | 29.4085 | 23 | 10 | Pseudomonas_aeruginosa | 1 | 1 | 432/6818030 | 0.0063 | 1.11 |
| Pseudomonas | 29.4085 | 23 | 10 | Pseudomonas_alcaligenes | 1 | 1 | 427/4406305 | 0.0097 | 1 |
| Pseudomonas | 29.4085 | 23 | 10 | Pseudomonas_fluorescens | 1 | 1 | 290/6136735 | 0.0047 | 1 |
| Pseudomonas | 29.4085 | 23 | 10 | Pseudomonas_mendocina | 1 | 1 | 338/5434353 | 0.0062 | 1 |
| Pseudomonas | 29.4085 | 23 | 10 | Pseudomonas_putida | 3 | 1 | 1235/6377271 | 0.0194 | 1 |
| Pseudomonas | 29.4085 | 23 | 10 | Pseudomonas_VLB120 | 1 | 1 | 196/5644569 | 0.0035 | 1 |
| Pseudomonas | 29.4085 | 23 | 10 | Pseudomonas_brassicacearum | 0 | 0 | 403/6976764 | 0.0058 | 1.08 |
| Pseudomonas | 29.4085 | 23 | 10 | Pseudomonas_chlororaphis | 0 | 0 | 198/7122173 | 0.0028 | 1 |
| Pseudomonas | 29.4085 | 23 | 10 | Pseudomonas_denitrificans | 0 | 0 | 286/5696307 | 0.005 | 1 |
| Pseudomonas | 29.4085 | 23 | 10 | Pseudomonas_entomophila | 0 | 0 | 425/5888780 | 0.0072 | 1 |
| Pseudomonas | 29.4085 | 23 | 10 | Pseudomonas_fulva | 0 | 0 | 384/4920769 | 0.0078 | 1 |
| Pseudomonas | 29.4085 | 23 | 10 | Pseudomonas_luteola | 0 | 0 | 43/4629099 | 0.0009 | 1 |
| Pseudomonas | 29.4085 | 23 | 10 | Pseudomonas_monteilii | 0 | 0 | 560/6000087 | 0.0093 | 1 |
| Pseudomonas | 29.4085 | 23 | 10 | Pseudomonas_oleovorans | 0 | 0 | 334/5006004 | 0.0067 | 1 |
| Pseudomonas | 29.4085 | 23 | 10 | Pseudomonas_oryzihabitans | 0 | 0 | 119/4834356 | 0.0025 | 1.26 |
| Pseudomonas | 29.4085 | 23 | 10 | Pseudomonas_poae | 0 | 0 | 324/5512241 | 0.0059 | 1 |
| Pseudomonas | 29.4085 | 23 | 10 | Pseudomonas_protegens | 0 | 0 | 337/6867980 | 0.0049 | 1 |
| Pseudomonas | 29.4085 | 23 | 10 | Pseudomonas_resinovorans | 0 | 0 | 613/6285863 | 0.0098 | 1 |
| Pseudomonas | 29.4085 | 23 | 10 | Pseudomonas_stutzeri | 0 | 0 | 96/4650155 | 0.0021 | 1 |
| Pseudomonas | 29.4085 | 23 | 10 | Pseudomonas_syringae | 0 | 0 | 142/6665031 | 0.0021 | 1 |
| Pseudomonas | 29.4085 | 23 | 10 | Pseudomonas_TKP | 0 | 0 | 400/7012672 | 0.0057 | 1 |
| Pseudomonas | 29.4085 | 23 | 10 | Pseudomonas_veronii | 0 | 0 | 515/6649830 | 0.0077 | 1 |
| Pseudomonas | 29.4085 | 23 | 10 | Pseudomonas_viridiflava | 0 | 0 | 187/5896435 | 0.0032 | 1 |
| Achromobacter | 18.4059 | 21 | 9 | Achromobacter_xylosoxidans | 11 | 5 | 2463/6813182 | 0.0362 | 1.04 |
| Achromobacter | 18.4059 | 21 | 9 | Achromobacter_denitrificans | 1 | 1 | 1076/6226215 | 0.0173 | 1 |
| Achromobacter | 18.4059 | 21 | 9 | Achromobacter_insuavis | 0 | 0 | 1058/6860925 | 0.0154 | 1.03 |
| Achromobacter | 18.4059 | 21 | 9 | Achromobacter_ruhlandii | 0 | 0 | 1167/6687427 | 0.0175 | 1.07 |
| Streptococcus | 13.4673 | 20 | 9 | Streptococcus_thermophilus | 10 | 4 | 550/1869510 | 0.0294 | 1 |
| Streptococcus | 13.4673 | 20 | 9 | Streptococcus_australis | 1 | 1 | 50/2130003 | 0.0023 | 1 |
| Streptococcus | 13.4673 | 20 | 9 | Streptococcus_cristatus | 1 | 1 | 50/2142100 | 0.0023 | 1 |
| Streptococcus | 13.4673 | 20 | 9 | Streptococcus_gordonii | 1 | 1 | 50/2196662 | 0.0023 | 1 |
| Streptococcus | 13.4673 | 20 | 9 | Streptococcus_I_P16 | 1 | 1 | 150/2023580 | 0.0074 | 1 |
| Streptococcus | 13.4673 | 20 | 9 | Streptococcus_oralis | 1 | 1 | 50/1958690 | 0.0026 | 1 |
| Streptococcus | 13.4673 | 20 | 9 | Streptococcus_sanguinis | 1 | 1 | 100/2388435 | 0.0042 | 1 |
| Streptococcus | 13.4673 | 20 | 9 | Streptococcus_sinensis | 1 | 1 | 100/2062993 | 0.0048 | 1 |
| Streptococcus | 13.4673 | 20 | 9 | Streptococcus_infantis | 0 | 0 | 50/2107541 | 0.0024 | 1 |
| Streptococcus | 13.4673 | 20 | 9 | Streptococcus_pneumoniae | 0 | 0 | 100/2150813 | 0.0046 | 1 |
| Streptococcus | 13.4673 | 20 | 9 | Streptococcus_pseudopneumoniae | 0 | 0 | 50/2190731 | 0.0023 | 1 |
| Streptococcus | 13.4673 | 20 | 9 | Streptococcus_vestibularis | 0 | 0 | 50/1882731 | 0.0027 | 1 |
| Sphingobium | 24.1963 | 15 | 7 | Sphingobium_yanoikuyae | 9 | 4 | 1943/5532659 | 0.0351 | 1.05 |
| Sphingobium | 24.1963 | 15 | 7 | Sphingobium_japonicum | 1 | 1 | 880/4196714 | 0.021 | 1 |
| Sphingobium | 24.1963 | 15 | 7 | Sphingobium_SYK | 2 | 1 | 735/4199332 | 0.0175 | 1.06 |
| Sphingobium | 24.1963 | 15 | 7 | Sphingobium_xenophagum | 2 | 1 | 875/4487790 | 0.0195 | 1 |
| Sphingobium | 24.1963 | 15 | 7 | Sphingobium_chlorophenolicum | 0 | 0 | 963/4449488 | 0.0216 | 1 |
| Azohydromonas | 27.4119 | 14 | 6 | Azohydromonas_lata | 14 | 6 | 9250/7186661 | 0.1287 | 1.03 |
| Methylibium | 49.2063 | 14 | 6 | Methylibium_petroleiphilum | 14 | 6 | 9240/4044195 | 0.2285 | 1.05 |
| Micrococcus | 7.5545 | 14 | 6 | Micrococcus_luteus | 9 | 4 | 544/2501097 | 0.0218 | 1 |
| Micrococcus | 7.5545 | 14 | 6 | Micrococcus_lylae | 1 | 1 | 243/2686748 | 0.009 | 1 |
| Micrococcus | 7.5545 | 14 | 6 | Micrococcus_terreus | 3 | 1 | 200/3087820 | 0.0065 | 1 |
| Rubrivivax | 49.9677 | 14 | 6 | Rubrivivax_gelatinosus | 14 | 6 | 11700/5043253 | 0.232 | 1.04 |
| Actinomyces | 6.0723 | 12 | 5 | Actinomyces_cardiffensis | 1 | 1 | 50/2214991 | 0.0023 | 1 |
| Actinomyces | 6.0723 | 12 | 5 | Actinomyces_dentalis | 3 | 1 | 195/3532293 | 0.0055 | 1 |
| Actinomyces | 6.0723 | 12 | 5 | Actinomyces_johnsonii | 2 | 1 | 100/3386293 | 0.003 | 1 |
| Actinomyces | 6.0723 | 12 | 5 | Actinomyces_turicensis | 1 | 1 | 50/1967795 | 0.0025 | 1 |
| Actinomyces | 6.0723 | 12 | 5 | Actinomyces_viscosus | 1 | 1 | 150/3134536 | 0.0048 | 1 |
| Actinomyces | 6.0723 | 12 | 5 | Actinomyces_denticolens | 0 | 0 | 46/2833168 | 0.0016 | 1 |
| Actinomyces | 6.0723 | 12 | 5 | Actinomyces_naeslundii | 0 | 0 | 100/3040449 | 0.0033 | 1 |
| Actinomyces | 6.0723 | 12 | 5 | Actinomyces_odontolyticus | 0 | 0 | 50/2432045 | 0.0021 | 1 |
| Actinomyces | 6.0723 | 12 | 5 | Actinomyces_oris | 0 | 0 | 50/3042917 | 0.0016 | 1 |
| Actinomyces | 6.0723 | 12 | 5 | Actinomyces_timonensis | 0 | 0 | 86/2932974 | 0.0029 | 1 |
| Comamonas | 32.6977 | 11 | 5 | Comamonas_testosteroni | 4 | 2 | 1603/5373644 | 0.0298 | 1 |
| Comamonas | 32.6977 | 11 | 5 | Comamonas_aquatica | 3 | 1 | 1929/3764434 | 0.0512 | 1.02 |
| Comamonas | 32.6977 | 11 | 5 | Comamonas_kerstersii | 1 | 1 | 541/3734555 | 0.0145 | 1 |
| Comamonas | 32.6977 | 11 | 5 | Comamonas_terrae | 2 | 1 | 2818/4717103 | 0.0597 | 1.04 |
| Ideonella | 41.339 | 11 | 5 | Ideonella_sakaiensis | 11 | 5 | 11887/6144323 | 0.1935 | 1.04 |
| Leuconostoc | 7.3969 | 12 | 5 | Leuconostoc_lactis | 9 | 4 | 495/1737502 | 0.0285 | 1 |
| Leuconostoc | 7.3969 | 12 | 5 | Leuconostoc_citreum | 2 | 1 | 100/1796284 | 0.0056 | 1 |
| Leuconostoc | 7.3969 | 12 | 5 | Leuconostoc_gelidum | 1 | 1 | 50/1893499 | 0.0026 | 1 |
| Massilia | 24.6395 | 12 | 5 | Massilia_putida | 7 | 3 | 2990/6987670 | 0.0428 | 1.07 |
| Massilia | 24.6395 | 12 | 5 | Massilia_alkalitolerans | 2 | 1 | 2770/6112424 | 0.0453 | 1.05 |
| Massilia | 24.6395 | 12 | 5 | Massilia_timonae | 1 | 1 | 1456/6136630 | 0.0237 | 1.05 |
| Pandoraea | 40.5905 | 11 | 5 | Pandoraea_norimbergensis | 3 | 1 | 2486/6167370 | 0.0403 | 1.06 |
| Pandoraea | 40.5905 | 11 | 5 | Pandoraea_pnomenusa | 1 | 1 | 2197/5584076 | 0.0393 | 1.01 |
| Pandoraea | 40.5905 | 11 | 5 | Pandoraea_sputorum | 2 | 1 | 1720/5742997 | 0.0299 | 1.04 |
| Pandoraea | 40.5905 | 11 | 5 | Pandoraea_apista | 0 | 0 | 1683/5609637 | 0.03 | 1.02 |
| Pandoraea | 40.5905 | 11 | 5 | Pandoraea_pulmonicola | 0 | 0 | 2939/5867621 | 0.0501 | 1.05 |
| Deinococcus | 8.8225 | 9 | 4 | Deinococcus_ficus | 1 | 1 | 242/4144928 | 0.0058 | 1 |
| Deinococcus | 8.8225 | 9 | 4 | Deinococcus_geothermalis | 3 | 1 | 150/2467205 | 0.0061 | 1 |
| Deinococcus | 8.8225 | 9 | 4 | Deinococcus_radiodurans | 2 | 1 | 327/3060986 | 0.0107 | 1 |
| Deinococcus | 8.8225 | 9 | 4 | Deinococcus_apachensis | 0 | 0 | 240/4453152 | 0.0054 | 1 |
| Deinococcus | 8.8225 | 9 | 4 | Deinococcus_deserti | 0 | 0 | 43/2819842 | 0.0015 | 1 |
| Deinococcus | 8.8225 | 9 | 4 | Deinococcus_gobiensis | 0 | 0 | 287/3137147 | 0.0091 | 1 |
| Deinococcus | 8.8225 | 9 | 4 | Deinococcus_peraridilitoris | 0 | 0 | 41/3881839 | 0.0011 | 1 |
| Deinococcus | 8.8225 | 9 | 4 | Deinococcus_proteolyticus | 0 | 0 | 50/2147060 | 0.0023 | 1 |
| Derxia | 10.0283 | 8 | 4 | Derxia_gummosa | 8 | 4 | 2326/5185308 | 0.0449 | 1.08 |
| Gordonia | 3.6306 | 10 | 4 | Gordonia_bronchialis | 4 | 2 | 200/5208602 | 0.0038 | 1 |
| Gordonia | 3.6306 | 10 | 4 | Gordonia_polyisoprenivorans | 4 | 2 | 248/5669805 | 0.0044 | 1 |
| Gordonia | 3.6306 | 10 | 4 | Gordonia_sputi | 1 | 1 | 50/4954549 | 0.001 | 1 |
| Gordonia | 3.6306 | 10 | 4 | Gordonia_amarae | 0 | 0 | 50/5307195 | 0.0009 | 1 |
| Gordonia | 3.6306 | 10 | 4 | Gordonia_effusa | 0 | 0 | 41/4704339 | 0.0009 | 1 |
| Gordonia | 3.6306 | 10 | 4 | Gordonia_KTR9 | 0 | 0 | 200/5441391 | 0.0037 | 1 |
| Gordonia | 3.6306 | 10 | 4 | Gordonia_neofelifaecis | 0 | 0 | 50/4257746 | 0.0012 | 1 |
| Gordonia | 3.6306 | 10 | 4 | Gordonia_paraffinivorans | 0 | 0 | 50/4633432 | 0.0011 | 1 |
| Gordonia | 3.6306 | 10 | 4 | Gordonia_rubripertincta | 0 | 0 | 44/5205555 | 0.0008 | 1 |
| Haematobacter | 2.757 | 8 | 4 | Haematobacter_missouriensis | 8 | 4 | 367/3960158 | 0.0093 | 1.22 |
| Haematobacter | 2.757 | 8 | 4 | Haematobacter_massiliensis | 0 | 0 | 100/4129200 | 0.0024 | 1 |
| Hylemonella | 14.3918 | 8 | 4 | Hylemonella_gracilis | 8 | 4 | 2526/3821625 | 0.0661 | 1.05 |
| Leucobacter | 4.0533 | 10 | 4 | Leucobacter_celer | 10 | 4 | 662/4143510 | 0.016 | 1.05 |
| Leucobacter | 4.0533 | 10 | 4 | Leucobacter_chironomi | 0 | 0 | 100/2964972 | 0.0034 | 1 |
| Microbacterium | 6.5855 | 9 | 4 | Microbacterium_aurum | 2 | 1 | 193/3424892 | 0.0056 | 1 |
| Microbacterium | 6.5855 | 9 | 4 | Microbacterium_laevaniformans | 2 | 1 | 100/3000545 | 0.0033 | 1 |
| Microbacterium | 6.5855 | 9 | 4 | Microbacterium_azadirachtae | 0 | 0 | 100/4243569 | 0.0024 | 1 |
| Microbacterium | 6.5855 | 9 | 4 | Microbacterium_hominis | 0 | 0 | 150/3506963 | 0.0043 | 1 |
| Microbacterium | 6.5855 | 9 | 4 | Microbacterium_oleivorans | 0 | 0 | 41/2916866 | 0.0014 | 1 |
| Microbacterium | 6.5855 | 9 | 4 | Microbacterium_paraoxydans | 0 | 0 | 50/3552313 | 0.0014 | 1 |
| Microbacterium | 6.5855 | 9 | 4 | Microbacterium_profundi | 0 | 0 | 50/3369467 | 0.0015 | 1 |
| Microbacterium | 6.5855 | 9 | 4 | Microbacterium_sediminis | 0 | 0 | 134/2760241 | 0.0049 | 1 |
| Microbacterium | 6.5855 | 9 | 4 | Microbacterium_testaceum | 0 | 0 | 145/3982034 | 0.0036 | 1 |
| Microbacterium | 6.5855 | 9 | 4 | Microbacterium_trichothecenolyticum | 0 | 0 | 140/4524680 | 0.0031 | 1 |
| Xanthobacter | 2.6371 | 8 | 4 | Xanthobacter_autotrophicus | 8 | 4 | 677/5308934 | 0.0128 | 1 |
| Aestuariibacter | 1.6291 | 6 | 3 | Aestuariibacter_aggregatus | 6 | 3 | 391/4910825 | 0.008 | 1 |
| Aquitalea | 4.0978 | 6 | 3 | Aquitalea_pelogenes | 6 | 3 | 887/4392639 | 0.0202 | 1 |
| Auricoccucs | 4.0096 | 7 | 3 | Auricoccus_indicus | 7 | 3 | 350/1745789 | 0.02 | 1 |
| Bordetella | 32.5611 | 6 | 3 | Bordetella_flabilis | 1 | 1 | 775/5835727 | 0.0133 | 1 |
| Bordetella | 32.5611 | 6 | 3 | Bordetella_holmesii | 2 | 1 | 611/3701221 | 0.0165 | 1 |
| Bordetella | 32.5611 | 6 | 3 | Bordetella_petrii | 2 | 1 | 863/5287950 | 0.0163 | 1.13 |
| Bordetella | 32.5611 | 6 | 3 | Bordetella_avium | 0 | 0 | 397/3732255 | 0.0106 | 1 |
| Bordetella | 32.5611 | 6 | 3 | Bordetella_bronchialis | 0 | 0 | 573/5966919 | 0.0096 | 1.08 |
| Bordetella | 32.5611 | 6 | 3 | Bordetella_bronchiseptica | 0 | 0 | 294/5339179 | 0.0055 | 1.16 |
| Bordetella | 32.5611 | 6 | 3 | Bordetella_hinzii | 0 | 0 | 1163/4912977 | 0.0237 | 1.04 |
| Bordetella | 32.5611 | 6 | 3 | Bordetella_parapertussis | 0 | 0 | 1290/4887379 | 0.0264 | 1.03 |
| Bordetella | 32.5611 | 6 | 3 | Bordetella_pertussis | 0 | 0 | 276/4386396 | 0.0063 | 1 |
| Bordetella | 32.5611 | 6 | 3 | Bordetella_trematum | 0 | 0 | 878/4480141 | 0.0196 | 1.19 |
| Brachybacterium | 2.6771 | 6 | 3 | Brachybacterium_alimentarium | 4 | 2 | 246/4263776 | 0.0058 | 1 |
| Brachybacterium | 2.6771 | 6 | 3 | Brachybacterium_muris | 2 | 1 | 195/3257876 | 0.006 | 1 |
| Brachybacterium | 2.6771 | 6 | 3 | Brachybacterium_faecium | 0 | 0 | 45/3614992 | 0.0012 | 1 |
| Cutibacterium | 7.4838 | 7 | 3 | Propionibacterium_namnetense | 5 | 2 | 792/2369944 | 0.0334 | 1 |
| Cutibacterium | 7.4838 | 7 | 3 | Cutibacterium_avidum | 1 | 1 | 95/2729848 | 0.0035 | 1 |
| Enterobacter | 4.0201 | 6 | 3 | Enterobacter_638 | 1 | 1 | 196/4518712 | 0.0043 | 1 |
| Enterobacter | 4.0201 | 6 | 3 | Enterobacter_cancerogenus | 1 | 1 | 146/4638743 | 0.0031 | 1 |
| Enterobacter | 4.0201 | 6 | 3 | Enterobacter_cloacae_complex_Hoffmann_cluster_III | 1 | 1 | 50/4633407 | 0.0011 | 1 |
| Enterobacter | 4.0201 | 6 | 3 | Enterobacter_asburiae | 0 | 0 | 100/4753402 | 0.0021 | 1 |
| Enterobacter | 4.0201 | 6 | 3 | Enterobacter_cloacae_complex_Hoffmann_cluster_IV | 0 | 0 | 50/4748414 | 0.0011 | 1 |
| Enterobacter | 4.0201 | 6 | 3 | Enterobacter_cloacae | 0 | 0 | 100/4879702 | 0.002 | 1 |
| Enterobacter | 4.0201 | 6 | 3 | Enterobacter_hormaechei | 0 | 0 | 50/4897485 | 0.001 | 1 |
| Enterobacter | 4.0201 | 6 | 3 | Enterobacter_kobei | 0 | 0 | 50/4880257 | 0.001 | 1 |
| Enterobacter | 4.0201 | 6 | 3 | Enterobacter_ludwigii | 0 | 0 | 50/4857439 | 0.001 | 1 |
| Enterobacter | 4.0201 | 6 | 3 | Enterobacter_massiliensis | 0 | 0 | 51/4924597 | 0.001 | 1 |
| Enterobacter | 4.0201 | 6 | 3 | Enterobacter_R4 | 0 | 0 | 50/5039027 | 0.001 | 1 |
| Enterobacter | 4.0201 | 6 | 3 | Enterobacter_xiangfangensis | 0 | 0 | 50/4661849 | 0.0011 | 1 |
| Herbaspirillum | 9.6121 | 6 | 3 | Herbaspirillum_seropedicae | 6 | 3 | 2540/5513887 | 0.0461 | 1.01 |
| Lactobacillus | 3.2902 | 7 | 3 | Lactobacillus_alimentarius | 4 | 2 | 200/2336775 | 0.0086 | 1 |
| Lactobacillus | 3.2902 | 7 | 3 | Lactobacillus_curvatus | 1 | 1 | 50/1986991 | 0.0025 | 1 |
| Lactobacillus | 3.2902 | 7 | 3 | Lactobacillus_iners | 1 | 1 | 50/1277759 | 0.0039 | 1 |
| Lactobacillus | 3.2902 | 7 | 3 | Lactobacillus_plantarum | 1 | 1 | 50/3418468 | 0.0015 | 1 |
| Lautropia | 6.0279 | 7 | 3 | Lautropia_mirabilis | 7 | 3 | 924/3151995 | 0.0293 | 1 |
| Myroides | 1.9264 | 7 | 3 | Myroides_odoratus | 1 | 1 | 50/4301842 | 0.0012 | 1 |
| Myroides | 1.9264 | 7 | 3 | Myroides_odoratimimus | 0 | 0 | 100/4366950 | 0.0023 | 1 |
| Myroides | 1.9264 | 7 | 3 | Myroides_profundi | 0 | 0 | 143/4064534 | 0.0035 | 1 |
| Myroides | 1.9264 | 7 | 3 | Myroides_xuanwuensis | 0 | 0 | 100/4017850 | 0.0025 | 1 |
| Prevotella | 3.2025 | 7 | 3 | Prevotella_bivia | 1 | 1 | 50/2580302 | 0.0019 | 1 |
| Prevotella | 3.2025 | 7 | 3 | Prevotella_copri | 1 | 1 | 50/3512733 | 0.0014 | 1 |
| Prevotella | 3.2025 | 7 | 3 | Prevotella_denticola | 1 | 1 | 50/2937589 | 0.0017 | 1 |
| Prevotella | 3.2025 | 7 | 3 | Prevotella_intermedia | 1 | 1 | 50/2699437 | 0.0019 | 1 |
| Prevotella | 3.2025 | 7 | 3 | Prevotella_melaninogenica | 1 | 1 | 50/3168282 | 0.0016 | 1 |
| Prevotella | 3.2025 | 7 | 3 | Hallella_seregens | 0 | 0 | 100/3267323 | 0.0031 | 1 |
| Prevotella | 3.2025 | 7 | 3 | Prevotella_loescheii | 0 | 0 | 45/3508889 | 0.0013 | 1 |
| Prevotella | 3.2025 | 7 | 3 | Prevotella_scopos | 0 | 0 | 100/3296420 | 0.003 | 1 |
| Rhodococcus | 1.9374 | 7 | 3 | Rhodococcus_ruber | 6 | 3 | 300/5465137 | 0.0055 | 1 |
| Rhodococcus | 1.9374 | 7 | 3 | Rhodococcus_erythropolis | 1 | 1 | 150/6516310 | 0.0023 | 1 |
| Rhodococcus | 1.9374 | 7 | 3 | Rhodococcus_opacus | 0 | 0 | 146/7913450 | 0.0018 | 1 |
| Abiotrophia | 1.9575 | 4 | 2 | Abiotrophia_defectiva | 4 | 2 | 200/2043469 | 0.0098 | 1 |
| Aeromonas | 3.8029 | 4 | 2 | Aeromonas_caviae | 3 | 1 | 194/4787529 | 0.0041 | 1 |
| Aeromonas | 3.8029 | 4 | 2 | Aeromonas_diversa | 0 | 0 | 147/4062867 | 0.0036 | 1 |
| Aeromonas | 3.8029 | 4 | 2 | Aeromonas_enteropelogenes | 0 | 0 | 51/4475188 | 0.0011 | 1.9 |
| Aeromonas | 3.8029 | 4 | 2 | Aeromonas_eucrenophila | 0 | 0 | 50/4540269 | 0.0011 | 1 |
| Aeromonas | 3.8029 | 4 | 2 | Aeromonas_media | 0 | 0 | 138/4777154 | 0.0029 | 1 |
| Aeromonas | 3.8029 | 4 | 2 | Aeromonas_salmonicida | 0 | 0 | 50/4702402 | 0.0011 | 1 |
| Aeromonas | 3.8029 | 4 | 2 | Aeromonas_simiae | 0 | 0 | 42/3988684 | 0.0011 | 1 |
| Aeromonas | 3.8029 | 4 | 2 | Aeromonas_taiwanensis | 0 | 0 | 87/4251941 | 0.002 | 1 |
| Bosea | 1.48 | 4 | 2 | Bosea_lupini | 4 | 2 | 437/6081108 | 0.0072 | 1 |
| Capnocytophaga | 2.1884 | 4 | 2 | Capnocytophaga_gingivalis | 1 | 1 | 100/2667498 | 0.0037 | 1 |
| Capnocytophaga | 2.1884 | 4 | 2 | Capnocytophaga_ochracea | 1 | 1 | 50/2612925 | 0.0019 | 1 |
| Capnocytophaga | 2.1884 | 4 | 2 | Capnocytophaga_sputigena | 2 | 1 | 100/2998485 | 0.0033 | 1 |
| Capnocytophaga | 2.1884 | 4 | 2 | Capnocytophaga_canimorsus | 0 | 0 | 42/2571406 | 0.0016 | 1 |
| Chryseobacterium | 1.6134 | 4 | 2 | Chryseobacterium_gleum | 1 | 1 | 50/5570680 | 0.0009 | 1 |
| Chryseobacterium | 1.6134 | 4 | 2 | Chryseobacterium_hominis | 3 | 1 | 200/2789542 | 0.0072 | 1 |
| Exiguobacterium | 1.5802 | 5 | 2 | Exiguobacterium_MH3 | 5 | 2 | 250/3164195 | 0.0079 | 1 |
| Fusobacterium | 3.7037 | 5 | 2 | Fusobacterium_hwasookii | 1 | 1 | 200/2463707 | 0.0081 | 1 |
| Fusobacterium | 3.7037 | 5 | 2 | Fusobacterium_periodonticum | 2 | 1 | 100/2615523 | 0.0038 | 1 |
| Fusobacterium | 3.7037 | 5 | 2 | Fusobacterium_gonidiaformans | 0 | 0 | 44/1930700 | 0.0023 | 1 |
| Fusobacterium | 3.7037 | 5 | 2 | Fusobacterium_nucleatum | 0 | 0 | 100/2507720 | 0.004 | 1 |
| Gulbenkiania | 3.8621 | 4 | 2 | Gulbenkiania_indica | 4 | 2 | 542/2848192 | 0.019 | 1 |
| Haemophilus | 4.3427 | 4 | 2 | Haemophilus_parainfluenzae | 3 | 1 | 400/2086875 | 0.0192 | 1 |
| Haemophilus | 4.3427 | 4 | 2 | Haemophilus_aegyptius | 0 | 0 | 50/1963793 | 0.0025 | 1 |
| Kytococcus | 1.4363 | 4 | 2 | Kytococcus_sedentarius | 4 | 2 | 200/2785024 | 0.0072 | 1 |
| Lactococcus | 2.8582 | 5 | 2 | Lactococcus_lactis | 2 | 1 | 100/2598144 | 0.0038 | 1 |
| Lactococcus | 2.8582 | 5 | 2 | Lactococcus_piscium | 3 | 1 | 250/2394138 | 0.0104 | 1 |
| Leptothrix | 41.9603 | 5 | 2 | Leptothrix_cholodnii | 5 | 2 | 9312/4909403 | 0.1897 | 1.06 |
| Mycobacterium | 5.9485 | 5 | 2 | Mycobacterium_aromaticivorans | 1 | 1 | 50/6297673 | 0.0008 | 1 |
| Mycobacterium | 5.9485 | 5 | 2 | Mycobacterium_chlorophenolicum | 1 | 1 | 50/7380230 | 0.0007 | 1 |
| Mycobacterium | 5.9485 | 5 | 2 | Mycobacterium_gilvum | 1 | 1 | 50/5619607 | 0.0009 | 1 |
| Mycobacterium | 5.9485 | 5 | 2 | Mycobacterium_alsense | 0 | 0 | 46/5694405 | 0.0008 | 1 |
| Mycobacterium | 5.9485 | 5 | 2 | Mycobacterium_asiaticum | 0 | 0 | 47/5939022 | 0.0008 | 1 |
| Mycobacterium | 5.9485 | 5 | 2 | Mycobacterium_aurum | 0 | 0 | 100/6038730 | 0.0017 | 1 |
| Mycobacterium | 5.9485 | 5 | 2 | Mycobacterium_avium | 0 | 0 | 43/5197664 | 0.0008 | 1 |
| Mycobacterium | 5.9485 | 5 | 2 | Mycobacterium_bohemicum | 0 | 0 | 50/5097360 | 0.001 | 1 |
| Mycobacterium | 5.9485 | 5 | 2 | Mycobacterium_chelonae | 0 | 0 | 45/5029817 | 0.0009 | 1 |
| Mycobacterium | 5.9485 | 5 | 2 | Mycobacterium_chubuense | 0 | 0 | 50/5583723 | 0.0009 | 1 |
| Mycobacterium | 5.9485 | 5 | 2 | Mycobacterium_colombiense | 0 | 0 | 50/5947639 | 0.0008 | 1 |
| Mycobacterium | 5.9485 | 5 | 2 | Mycobacterium_doricum | 0 | 0 | 50/3952833 | 0.0013 | 1 |
| Mycobacterium | 5.9485 | 5 | 2 | Mycobacterium_europaeum | 0 | 0 | 100/6152643 | 0.0016 | 1 |
| Mycobacterium | 5.9485 | 5 | 2 | Mycobacterium_franklinii | 0 | 0 | 50/5023905 | 0.001 | 1 |
| Mycobacterium | 5.9485 | 5 | 2 | Mycobacterium_goodii | 0 | 0 | 50/7105933 | 0.0007 | 1 |
| Mycobacterium | 5.9485 | 5 | 2 | Mycobacterium_hassiacum | 0 | 0 | 44/5083305 | 0.0009 | 1 |
| Mycobacterium | 5.9485 | 5 | 2 | Mycobacterium_holsaticum | 0 | 0 | 44/5751856 | 0.0008 | 1 |
| Mycobacterium | 5.9485 | 5 | 2 | Mycobacterium_intermedium | 0 | 0 | 100/6857760 | 0.0015 | 1 |
| Mycobacterium | 5.9485 | 5 | 2 | Mycobacterium_komaniense | 0 | 0 | 100/5379610 | 0.0019 | 1 |
| Mycobacterium | 5.9485 | 5 | 2 | Mycobacterium_llatzerense | 0 | 0 | 91/6700160 | 0.0014 | 1 |
| Mycobacterium | 5.9485 | 5 | 2 | Mycobacterium_marinum | 0 | 0 | 50/6636827 | 0.0008 | 1 |
| Mycobacterium | 5.9485 | 5 | 2 | Mycobacterium_neoaurum | 0 | 0 | 50/5421267 | 0.0009 | 1 |
| Mycobacterium | 5.9485 | 5 | 2 | Mycobacterium_obuense | 0 | 0 | 50/6384246 | 0.0008 | 1 |
| Mycobacterium | 5.9485 | 5 | 2 | Mycobacterium_septicum | 0 | 0 | 92/6908895 | 0.0013 | 1 |
| Mycobacterium | 5.9485 | 5 | 2 | Mycobacterium_simiae | 0 | 0 | 41/5938797 | 0.0007 | 1 |
| Mycobacterium | 5.9485 | 5 | 2 | Mycobacterium_vaccae | 0 | 0 | 50/6235754 | 0.0008 | 1 |
| Mycobacterium | 5.9485 | 5 | 2 | Mycobacterium_VKM | 0 | 0 | 50/5438192 | 0.0009 | 1 |
| Mycobacterium | 5.9485 | 5 | 2 | Mycobacterium_vulneris | 0 | 0 | 45/7088049 | 0.0006 | 1 |
| Mycobacterium | 5.9485 | 5 | 2 | Mycobacterium_yongonense | 0 | 0 | 50/5521023 | 0.0009 | 1 |
| Novosphingobium | 8.0716 | 4 | 2 | Novosphingobium_aromaticivorans | 4 | 2 | 784/3561584 | 0.022 | 1 |
| Novosphingobium | 8.0716 | 4 | 2 | Novosphingobium_PP1Y | 0 | 0 | 677/3911486 | 0.0173 | 1 |
| Pectobacterium | 1.6207 | 5 | 2 | Pectobacterium_carotovorum | 2 | 1 | 246/4862913 | 0.0051 | 1 |
| Pectobacterium | 1.6207 | 5 | 2 | Pectobacterium_atrosepticum | 0 | 0 | 50/5064019 | 0.001 | 1 |
| Pectobacterium | 1.6207 | 5 | 2 | Pectobacterium_wasabiae | 0 | 0 | 95/5063892 | 0.0019 | 1 |
| Ramlibacter | 24.5689 | 4 | 2 | Ramlibacter_tataouinensis | 4 | 2 | 4559/4070193 | 0.112 | 1.06 |
| Stenotrophomonas | 4.2131 | 5 | 2 | Stenotrophomonas_maltophilia | 5 | 2 | 927/4509724 | 0.0206 | 1 |
| Veillonella | 2.8213 | 5 | 2 | Veillonella_parvula | 3 | 1 | 248/2132142 | 0.0116 | 1 |
| Veillonella | 2.8213 | 5 | 2 | Veillonella_atypica | 0 | 0 | 50/2099783 | 0.0024 | 1 |
| Verminephrobacter | 8.8023 | 4 | 2 | Verminephrobacter_eiseniae | 4 | 2 | 2225/5566749 | 0.04 | 1.07 |
| Acidiphilium | 1.9236 | 1 | 1 | Acidiphilium_multivorum | 1 | 1 | 209/3749411 | 0.0056 | 1.19 |
| Acidiphilium | 1.9236 | 1 | 1 | Acidiphilium_cryptum | 0 | 0 | 98/3389227 | 0.0029 | 1 |
| Acidithiobacillus | 0.9588 | 2 | 1 | Acidithiobacillus_ferrivorans | 2 | 1 | 63/3207552 | 0.002 | 1.59 |
| Acidithiobacillus | 0.9588 | 2 | 1 | Acidithiobacillus_ferrooxidans | 0 | 0 | 50/2982397 | 0.0017 | 1 |
| Aeromicrobium | 0.5844 | 1 | 1 | Aeromicrobium_choanae | 1 | 1 | 100/3422026 | 0.0029 | 1 |
| Aggregatibacter | 1.3617 | 2 | 1 | Aggregatibacter_aphrophilus | 1 | 1 | 100/2313035 | 0.0043 | 1 |
| Aggregatibacter | 1.3617 | 2 | 1 | Aggregatibacter_segnis | 1 | 1 | 50/2012050 | 0.0025 | 1 |
| Agrobacterium | 1.5738 | 1 | 1 | Agrobacterium_albertimagni | 0 | 0 | 44/5085601 | 0.0009 | 1 |
| Agrobacterium | 1.5738 | 1 | 1 | Agrobacterium_fabrum | 0 | 0 | 150/4917167 | 0.0031 | 1 |
| Agrobacterium | 1.5738 | 1 | 1 | Agrobacterium_H13 | 0 | 0 | 96/4972229 | 0.0019 | 1 |
| Agrobacterium | 1.5738 | 1 | 1 | Agrobacterium_tumefaciens | 0 | 0 | 100/5481605 | 0.0018 | 1 |
| Agrococcus | 0.6384 | 1 | 1 | Agrococcus_jejuensis | 1 | 1 | 50/3400178 | 0.0015 | 1 |
| Agrococcus | 0.6384 | 1 | 1 | Agrococcus_baldri | 0 | 0 | 46/2904536 | 0.0016 | 1 |
| Alcaligenes | 0.9448 | 3 | 1 | Alcaligenes_faecalis | 3 | 1 | 196/4233756 | 0.0046 | 1 |
| Algoriella | 0.3062 | 1 | 1 | Algoriella_xinjiangensis | 1 | 1 | 50/3266341 | 0.0015 | 1 |
| Alishewanella | 0.5727 | 1 | 1 | Alishewanella_agri | 1 | 1 | 100/3491999 | 0.0029 | 1 |
| Alteromonas | 1.2737 | 3 | 1 | Alteromonas_macleodii | 3 | 1 | 250/4653851 | 0.0054 | 1 |
| Alteromonas | 1.2737 | 3 | 1 | Alteromonas_lipolytica | 0 | 0 | 50/5018651 | 0.001 | 1 |
| Amycolatopsis | 0.4525 | 1 | 1 | Amycolatopsis_marina | 1 | 1 | 50/7021279 | 0.0007 | 1 |
| Amycolatopsis | 0.4525 | 1 | 1 | Amycolatopsis_benzoatilytica | 0 | 0 | 50/8704271 | 0.0006 | 1 |
| Amycolatopsis | 0.4525 | 1 | 1 | Amycolatopsis_mediterranei | 0 | 0 | 100/10246920 | 0.001 | 1 |
| Anaerococcus | 0.9837 | 1 | 1 | Anaerococcus_prevotii | 1 | 1 | 50/1883067 | 0.0027 | 1 |
| Anaerococcus | 0.9837 | 1 | 1 | Anaerococcus_lactolyticus | 0 | 0 | 46/2209175 | 0.0021 | 1 |
| Anaeromyxobacter | 0.7903 | 1 | 1 | Anaeromyxobacter_K | 0 | 0 | 196/5061632 | 0.0039 | 1 |
| Aquimonas | 1.5807 | 1 | 1 | Aquimonas_voraii | 1 | 1 | 338/4428557 | 0.0076 | 1 |
| Arsenicicoccus | 0.5005 | 1 | 1 | Arsenicicoccus_bolidensis | 1 | 1 | 91/3996173 | 0.0023 | 1 |
| Arthrobacter | 0.9421 | 1 | 1 | Arthrobacter_arilaitensis | 1 | 1 | 95/3859257 | 0.0025 | 1 |
| Arthrobacter | 0.9421 | 1 | 1 | Arthrobacter_FB24 | 0 | 0 | 50/4698945 | 0.0011 | 1 |
| Arthrobacter | 0.9421 | 1 | 1 | Arthrobacter_nitroguajacolicus | 0 | 0 | 41/4736495 | 0.0009 | 1 |
| Atopobium | 0.6647 | 1 | 1 | Atopobium_vaginae | 1 | 1 | 50/1504394 | 0.0033 | 1 |
| Azoarcus | 15.6918 | 1 | 1 | Azoarcus_BH72 | 1 | 1 | 1502/4376040 | 0.0343 | 1.03 |
| Azoarcus | 15.6918 | 1 | 1 | Azoarcus_KH32C | 0 | 0 | 939/5081166 | 0.0185 | 1.1 |
| Azoarcus | 15.6918 | 1 | 1 | Azoarcus_toluclasticus | 0 | 0 | 1043/5926443 | 0.0176 | 1.09 |
| Azospira | 3.9401 | 1 | 1 | Azospira_oryzae | 1 | 1 | 676/3806980 | 0.0178 | 1.06 |
| Azovibrio | 2.4837 | 1 | 1 | Azovibrio_restrictus | 1 | 1 | 399/4026211 | 0.0099 | 1.21 |
| Bacillus | 1.4559 | 3 | 1 | Bacillus_anthracis | 0 | 0 | 100/5228842 | 0.0019 | 1 |
| Bacillus | 1.4559 | 3 | 1 | Bacillus_galactosidilyticus | 0 | 0 | 50/5187773 | 0.001 | 1 |
| Bacillus | 1.4559 | 3 | 1 | Bacillus_subtilis | 0 | 0 | 50/4251652 | 0.0012 | 1 |
| Bacillus | 1.4559 | 3 | 1 | Bacillus_toyonensis | 0 | 0 | 100/4940474 | 0.002 | 1 |
| Bacillus | 1.4559 | 3 | 1 | Bacillus_vallismortis | 0 | 0 | 50/4155525 | 0.0012 | 1 |
| Bacteroides | 0.3657 | 1 | 1 | Bacteroides_dorei | 1 | 1 | 50/5444912 | 0.0009 | 1 |
| Bacteroides | 0.3657 | 1 | 1 | Bacteroides_caccae | 0 | 0 | 41/5493167 | 0.0007 | 1 |
| Blastococcus | 0.4102 | 1 | 1 | Blastococcus_saxobsidens | 1 | 1 | 100/4875340 | 0.0021 | 1 |
| Brachymonas | 14.7434 | 1 | 1 | Brachymonas_denitrificans | 1 | 1 | 1892/2713070 | 0.0697 | 1.02 |
| Brevibacterium | 1.4282 | 3 | 1 | Brevibacterium_casei | 1 | 1 | 100/3769980 | 0.0027 | 1 |
| Brevibacterium | 1.4282 | 3 | 1 | Brevibacterium_mcbrellneri | 1 | 1 | 50/2562754 | 0.002 | 1 |
| Brevibacterium | 1.4282 | 3 | 1 | Brevibacterium_epidermidis | 0 | 0 | 50/3703501 | 0.0014 | 1 |
| Brevibacterium | 1.4282 | 3 | 1 | Brevibacterium_linens | 0 | 0 | 50/4209935 | 0.0012 | 1 |
| Brochothrix | 0.3825 | 1 | 1 | Brochothrix_thermosphacta | 1 | 1 | 50/2614483 | 0.0019 | 1 |
| Caballeronia | 19.1418 | 2 | 1 | Caballeronia_sordidicola | 2 | 1 | 2254/10264041 | 0.022 | 1.06 |
| Caballeronia | 19.1418 | 2 | 1 | Caballeronia_concitans | 0 | 0 | 4004/6166631 | 0.0649 | 1.06 |
| Caldimonas | 19.8132 | 3 | 1 | Caldimonas_manganoxidans | 3 | 1 | 3246/3532996 | 0.0919 | 1.04 |
| Campylobacter | 1.0273 | 1 | 1 | Campylobacter_mucosalis | 1 | 1 | 50/1752144 | 0.0029 | 1 |
| Campylobacter | 1.0273 | 1 | 1 | Campylobacter_showae | 0 | 0 | 50/2190071 | 0.0023 | 1 |
| Carnobacterium | 0.7831 | 2 | 1 | Carnobacterium_divergens | 1 | 1 | 50/2590974 | 0.0019 | 1 |
| Carnobacterium | 0.7831 | 2 | 1 | Carnobacterium_jeotgali | 1 | 1 | 50/2518354 | 0.002 | 1 |
| Castellaniella | 3.5137 | 1 | 1 | Castellaniella_caeni | 1 | 1 | 578/3415197 | 0.0169 | 1 |
| Caulobacter | 3.836 | 2 | 1 | Caulobacter_crescentus | 1 | 1 | 300/4042929 | 0.0074 | 1 |
| Caulobacter | 3.836 | 2 | 1 | Caulobacter_K31 | 0 | 0 | 325/5477872 | 0.0059 | 1 |
| Caulobacter | 3.836 | 2 | 1 | Caulobacter_segnis | 0 | 0 | 234/4655622 | 0.005 | 1 |
| Cedecea | 0.6106 | 1 | 1 | Cedecea_davisae | 0 | 0 | 100/4887001 | 0.002 | 1 |
| Cedecea | 0.6106 | 1 | 1 | Cedecea_neteri | 0 | 0 | 50/4965436 | 0.001 | 1 |
| Cellvibrio | 1.0925 | 3 | 1 | Cellvibrio_japonicus | 3 | 1 | 236/4576573 | 0.0052 | 1 |
| Chromobacterium | 4.7239 | 2 | 1 | Chromobacterium_haemolyticum | 2 | 1 | 467/5030819 | 0.0093 | 1.04 |
| Chromobacterium | 4.7239 | 2 | 1 | Chromobacterium_violaceum | 0 | 0 | 633/4751080 | 0.0133 | 1 |
| Croceicoccus | 3.0876 | 3 | 1 | Croceicoccus_mobilis | 3 | 1 | 637/4210389 | 0.0151 | 1 |
| Curvibacter | 17.3018 | 3 | 1 | Curvibacter_delicatus | 3 | 1 | 2948/3756831 | 0.0785 | 1.07 |
| Dermacoccus | 0.3301 | 1 | 1 | Dermacoccus_nishinomiyaensis | 1 | 1 | 50/3029021 | 0.0017 | 1 |
| Devosia | 1.4291 | 3 | 1 | Devosia_chinhatensis | 3 | 1 | 189/3498689 | 0.0054 | 1.32 |
| Diaphorobacter | 7.8793 | 1 | 1 | Diaphorobacter_polyhydroxybutyrativorans | 1 | 1 | 1563/4061276 | 0.0385 | 1 |
| Dolosigranulum | 1.611 | 3 | 1 | Dolosigranulum_pigrum | 3 | 1 | 150/1862145 | 0.0081 | 1 |
| Donghicola | 0.2259 | 1 | 1 | Donghicola_eburneus | 1 | 1 | 50/4427046 | 0.0011 | 1 |
| Duganella | 5.0928 | 1 | 1 | Duganella_sacchari | 1 | 1 | 1592/6676036 | 0.0238 | 1.03 |
| Dyella | 1.6559 | 1 | 1 | Dyella_japonica | 1 | 1 | 340/4831185 | 0.007 | 1.14 |
| Enhydrobacter | 1.4771 | 2 | 1 | Enhydrobacter_aerosaccus | 2 | 1 | 493/6770053 | 0.0073 | 1 |
| Ensifer | 0.4913 | 1 | 1 | Ensifer_adhaerens | 1 | 1 | 100/4071185 | 0.0025 | 1 |
| Erythrobacter | 6.3651 | 1 | 1 | Erythrobacter_flavus | 1 | 1 | 299/3184307 | 0.0094 | 1 |
| Erythrobacter | 6.3651 | 1 | 1 | Erythrobacter_litoralis | 0 | 0 | 144/3052398 | 0.0047 | 1 |
| Erythrobacter | 6.3651 | 1 | 1 | Erythrobacter_vulgaris | 0 | 0 | 484/2858686 | 0.0169 | 1 |
| Faecalibacterium | 0.3011 | 1 | 1 | Faecalibacterium_prausnitzii | 1 | 1 | 50/3321367 | 0.0015 | 1 |
| Flavobacterium | 1.8166 | 1 | 1 | Flavobacterium_branchiophilum | 1 | 1 | 50/3559884 | 0.0014 | 1 |
| Flavobacterium | 1.8166 | 1 | 1 | Flavobacterium_aquatile | 0 | 0 | 91/3488993 | 0.0026 | 1 |
| Flavobacterium | 1.8166 | 1 | 1 | Flavobacterium_columnare | 0 | 0 | 50/3329120 | 0.0015 | 1 |
| Flavobacterium | 1.8166 | 1 | 1 | Flavobacterium_indicum | 0 | 0 | 51/2993089 | 0.0017 | 1 |
| Flavobacterium | 1.8166 | 1 | 1 | Flavobacterium_johnsoniae | 0 | 0 | 100/6096872 | 0.0016 | 1 |
| Formivibrio | 1.6271 | 1 | 1 | Formivibrio_citricus | 1 | 1 | 250/3072901 | 0.0081 | 1 |
| Gardnerella | 1.7992 | 3 | 1 | Gardnerella_vaginalis | 3 | 1 | 150/1667406 | 0.009 | 1 |
| Gemmiger | 0.3084 | 1 | 1 | Gemmiger_formicilis | 1 | 1 | 50/3242479 | 0.0015 | 1 |
| Granulicatella | 0.5135 | 1 | 1 | Granulicatella_adiacens | 1 | 1 | 50/1947256 | 0.0026 | 1 |
| Halorientalis | 0.2053 | 1 | 1 | Halorientalis_persicus | 1 | 1 | 46/4870958 | 0.0009 | 1 |
| Hydrogenophaga | 14.9739 | 2 | 1 | Hydrogenophaga_taeniospiralis | 2 | 1 | 3687/5275851 | 0.0699 | 1.03 |
| Hyphomicrobium | 1.2192 | 1 | 1 | Hyphomicrobium_MC1 | 1 | 1 | 100/4757528 | 0.0021 | 1 |
| Hyphomicrobium | 1.2192 | 1 | 1 | Hyphomicrobium_denitrificans | 0 | 0 | 91/3808687 | 0.0024 | 1 |
| Hyphomicrobium | 1.2192 | 1 | 1 | Hyphomicrobium_nitrativorans | 0 | 0 | 42/3653837 | 0.0011 | 1 |
| Immundisolibacter | 3.3914 | 1 | 1 | Immundisolibacter_cernigliae | 1 | 1 | 547/3243537 | 0.0169 | 1 |
| Janibacter | 0.3128 | 1 | 1 | Janibacter_melonis | 1 | 1 | 50/3196938 | 0.0016 | 1 |
| Janthinobacterium | 2.4329 | 1 | 1 | Janthinobacterium_Marseille | 1 | 1 | 491/4110251 | 0.0119 | 1 |
| Jeotgalicoccus | 0.4564 | 1 | 1 | Jeotgalicoccus_saudimassiliensis | 1 | 1 | 50/2191063 | 0.0023 | 1 |
| Klebsiella | 0.3622 | 1 | 1 | Klebsiella_variicola | 0 | 0 | 100/5521203 | 0.0018 | 1 |
| Kosakonia | 0.4304 | 2 | 1 | Kosakonia_cowanii | 2 | 1 | 98/4646998 | 0.0021 | 1 |
| Leclercia | 0.6245 | 1 | 1 | Leclercia_adecarboxylata | 1 | 1 | 147/4803917 | 0.0031 | 1 |
| Leptospira | 0.4198 | 2 | 1 | Leptospira_noguchii | 2 | 1 | 100/4763760 | 0.0021 | 1 |
| Leptotrichia | 1.6221 | 3 | 1 | Leptotrichia_hofstadii | 1 | 1 | 50/2560908 | 0.002 | 1 |
| Leptotrichia | 1.6221 | 3 | 1 | Leptotrichia_buccalis | 0 | 0 | 100/2465610 | 0.0041 | 1 |
| Leptotrichia | 1.6221 | 3 | 1 | Leptotrichia_wadei | 0 | 0 | 50/2378839 | 0.0021 | 1 |
| Limnohabitans | 4.4282 | 2 | 1 | Limnohabitans_planktonicus | 2 | 1 | 972/4742314 | 0.0205 | 1.04 |
| Lysobacter | 1.5212 | 1 | 1 | Lysobacter_antibioticus | 1 | 1 | 355/5916388 | 0.006 | 1.22 |
| Macrococcus | 0.4757 | 1 | 1 | Macrococcus_caseolyticus | 1 | 1 | 50/2102324 | 0.0024 | 1 |
| Magnetospirillum | 2.3372 | 1 | 1 | Magnetospirillum_magneticum | 1 | 1 | 196/4967148 | 0.0039 | 1 |
| Magnetospirillum | 2.3372 | 1 | 1 | Magnetospirillum_caucaseum | 0 | 0 | 141/4872864 | 0.0029 | 1 |
| Magnetospirillum | 2.3372 | 1 | 1 | Magnetospirillum_gryphiswaldense | 0 | 0 | 194/4365796 | 0.0044 | 1 |
| Marinobacter | 2.0947 | 2 | 1 | Marinobacter_adhaerens | 2 | 1 | 183/4421911 | 0.0041 | 1.04 |
| Marinobacter | 2.0947 | 2 | 1 | Marinobacter_aquaeolei | 0 | 0 | 150/4326849 | 0.0035 | 1 |
| Marinobacter | 2.0947 | 2 | 1 | Marinobacter_BSs20148 | 0 | 0 | 46/4063864 | 0.0011 | 1 |
| Marinobacter | 2.0947 | 2 | 1 | Marinobacter_hydrocarbonoclasticus | 0 | 0 | 50/3989480 | 0.0013 | 1 |
| Marmoricola | 0.4803 | 1 | 1 | Marmoricola_aequoreus | 1 | 1 | 100/4163916 | 0.0024 | 1 |
| Melaminivora | 24.8663 | 1 | 1 | Melaminivora_alkalimesophila | 1 | 1 | 3579/3016125 | 0.1187 | 1.01 |
| Mesorhizobium | 3.3624 | 2 | 1 | Mesorhizobium_ciceri | 1 | 1 | 298/6264489 | 0.0048 | 1 |
| Mesorhizobium | 3.3624 | 2 | 1 | Mesorhizobium_australicum | 0 | 0 | 282/6200534 | 0.0045 | 1 |
| Mesorhizobium | 3.3624 | 2 | 1 | Mesorhizobium_loti | 0 | 0 | 239/7036071 | 0.0034 | 1 |
| Mesorhizobium | 3.3624 | 2 | 1 | Mesorhizobium_opportunistum | 0 | 0 | 242/6884444 | 0.0035 | 1 |
| Methylophilus | 0.3496 | 1 | 1 | Methylophilus_methylotrophus | 1 | 1 | 50/2860349 | 0.0017 | 1 |
| Methyloversatilis | 6.0398 | 3 | 1 | Methyloversatilis_discipulorum | 3 | 1 | 1231/4304808 | 0.0286 | 1 |
| Microterricola | 0.271 | 1 | 1 | Microterricola_viridarii | 1 | 1 | 46/3689726 | 0.0012 | 1 |
| Microvirga | 0.4963 | 1 | 1 | Microvirga_flocculans | 1 | 1 | 100/4030071 | 0.0025 | 1 |
| Mobiluncus | 0.8606 | 2 | 1 | Mobiluncus_curtisii | 1 | 1 | 50/2146480 | 0.0023 | 1 |
| Mobiluncus | 0.8606 | 2 | 1 | Mobiluncus_mulieris | 1 | 1 | 50/2533863 | 0.002 | 1 |
| Morganella | 0.5264 | 2 | 1 | Morganella_morganii | 2 | 1 | 100/3799539 | 0.0026 | 1 |
| Nitrobacter | 3.8058 | 3 | 1 | Nitrobacter_hamburgensis | 2 | 1 | 426/4406967 | 0.0097 | 1 |
| Nitrobacter | 3.8058 | 3 | 1 | Nitrobacter_winogradskyi | 0 | 0 | 292/3402093 | 0.0086 | 1 |
| Nocardia | 1.5054 | 1 | 1 | Nocardia_farcinica | 1 | 1 | 50/6021225 | 0.0008 | 1 |
| Nocardia | 1.5054 | 1 | 1 | Nocardia_africana | 0 | 0 | 86/7811902 | 0.0011 | 1 |
| Nocardia | 1.5054 | 1 | 1 | Nocardia_aobensis | 0 | 0 | 42/7551032 | 0.0006 | 1 |
| Nocardia | 1.5054 | 1 | 1 | Nocardia_araoensis | 0 | 0 | 43/7726046 | 0.0006 | 1 |
| Nocardia | 1.5054 | 1 | 1 | Nocardia_beijingensis | 0 | 0 | 50/7478959 | 0.0007 | 1 |
| Nocardia | 1.5054 | 1 | 1 | Nocardia_nova | 0 | 0 | 45/8348532 | 0.0005 | 1 |
| Nocardia | 1.5054 | 1 | 1 | Nocardia_otitidiscaviarum | 0 | 0 | 47/7896203 | 0.0006 | 1 |
| Nocardia | 1.5054 | 1 | 1 | Nocardia_terpenica | 0 | 0 | 92/9282248 | 0.001 | 1 |
| Nocardia | 1.5054 | 1 | 1 | Nocardia_transvalensis | 0 | 0 | 41/8384573 | 0.0005 | 1 |
| Nocardia | 1.5054 | 1 | 1 | Nocardia_vulneris | 0 | 0 | 45/9378340 | 0.0005 | 1 |
| Nocardioides | 0.8023 | 1 | 1 | Nocardioides_JS614 | 1 | 1 | 191/4985871 | 0.0038 | 1 |
| Noviherbaspirillum | 7.4016 | 2 | 1 | Herbaspirillum_massiliense | 2 | 1 | 1356/4188289 | 0.0324 | 1.09 |
| Ochrobactrum | 0.6325 | 1 | 1 | Ochrobactrum_anthropi | 1 | 1 | 100/4783208 | 0.0021 | 1 |
| Ochrobactrum | 0.6325 | 1 | 1 | Ochrobactrum_intermedium | 0 | 0 | 45/4665240 | 0.001 | 1 |
| Pantoea | 3.2078 | 2 | 1 | Pantoea_dispersa | 1 | 1 | 500/4951455 | 0.0101 | 1 |
| Pantoea | 3.2078 | 2 | 1 | Pantoea_vagans | 1 | 1 | 50/4024986 | 0.0012 | 1 |
| Pantoea | 3.2078 | 2 | 1 | Pantoea_agglomerans | 0 | 0 | 100/4050093 | 0.0025 | 1 |
| Pantoea | 3.2078 | 2 | 1 | Pantoea_ananatis | 0 | 0 | 43/4605545 | 0.0009 | 1 |
| Pantoea | 3.2078 | 2 | 1 | Pantoea_At | 0 | 0 | 50/4368708 | 0.0011 | 1 |
| Paracoccus | 5.4303 | 2 | 1 | Paracoccus_yeei | 1 | 1 | 535/4429585 | 0.0121 | 1 |
| Paracoccus | 5.4303 | 2 | 1 | Paracoccus_aminophilus | 0 | 0 | 241/3613807 | 0.0067 | 1 |
| Paracoccus | 5.4303 | 2 | 1 | Paracoccus_denitrificans | 0 | 0 | 145/4582379 | 0.0032 | 1 |
| Paracoccus | 5.4303 | 2 | 1 | Paracoccus_versutus | 0 | 0 | 237/5502608 | 0.0043 | 1 |
| Parvibaculum | 1.2772 | 1 | 1 | Parvibaculum_lavamentivorans | 1 | 1 | 242/3914745 | 0.0062 | 1 |
| Peptoniphilus | 0.5401 | 1 | 1 | Peptoniphilus_senegalensis | 0 | 0 | 50/1851620 | 0.0027 | 1 |
| Planomicrobium | 0.2552 | 1 | 1 | Planomicrobium_glaciei | 1 | 1 | 50/3917936 | 0.0013 | 1 |
| Polaromonas | 21.3165 | 3 | 1 | Polaromonas_JS666 | 2 | 1 | 2132/5200264 | 0.041 | 1.03 |
| Polaromonas | 21.3165 | 3 | 1 | Polaromonas_naphthalenivorans | 1 | 1 | 2598/4410291 | 0.0589 | 1.02 |
| Porphyromonas | 1.8648 | 3 | 1 | Porphyromonas_catoniae | 1 | 1 | 50/2101655 | 0.0024 | 1 |
| Porphyromonas | 1.8648 | 3 | 1 | Porphyromonas_endodontalis | 1 | 1 | 94/2064868 | 0.0046 | 1 |
| Porphyromonas | 1.8648 | 3 | 1 | Porphyromonas_gingivalis | 1 | 1 | 50/2378872 | 0.0021 | 1 |
| Providencia | 0.2092 | 1 | 1 | Providencia_rettgeri | 1 | 1 | 50/4780676 | 0.001 | 1 |
| Pseudoxanthomonas | 7.7751 | 3 | 1 | Pseudoxanthomonas_Mexicana | 1 | 1 | 470/3943279 | 0.0119 | 1 |
| Pseudoxanthomonas | 7.7751 | 3 | 1 | Pseudoxanthomonas_suwonensis | 1 | 1 | 425/3419049 | 0.0124 | 1 |
| Pseudoxanthomonas | 7.7751 | 3 | 1 | Pseudoxanthomonas_spadix | 0 | 0 | 437/3452554 | 0.0127 | 1 |
| Psychrobacter | 1.404 | 2 | 1 | Psychrobacter_arcticus | 1 | 1 | 100/2650701 | 0.0038 | 1 |
| Psychrobacter | 1.404 | 2 | 1 | Psychrobacter_cryohalolentis | 0 | 0 | 50/3059876 | 0.0016 | 1 |
| Psychrobacter | 1.404 | 2 | 1 | Psychrobacter_phenylpyruvicus | 0 | 0 | 50/3099103 | 0.0016 | 1 |
| Pusillimonas | 1.8024 | 2 | 1 | Pusillimonas_T7 | 2 | 1 | 334/3883605 | 0.0086 | 1 |
| Raoultella | 0.6995 | 1 | 1 | Raoultella_terrigena | 1 | 1 | 150/5717287 | 0.0026 | 1 |
| Raoultella | 0.6995 | 1 | 1 | Raoultella_ornithinolytica | 0 | 0 | 50/5719363 | 0.0009 | 1 |
| Rhodobacter | 4.3284 | 1 | 1 | Rhodanobacter_2APBS1 | 1 | 1 | 544/4225490 | 0.0129 | 1.15 |
| Rhodobacter | 4.3284 | 1 | 1 | Rhodobacter_capsulatus | 0 | 0 | 128/3738958 | 0.0034 | 1 |
| Rhodobacter | 4.3284 | 1 | 1 | Rhodobacter_sphaeroides | 0 | 0 | 92/4450439 | 0.0021 | 1 |
| Rhodoferax | 4.2442 | 1 | 1 | Rhodoferax_ferrireducens | 1 | 1 | 907/4712337 | 0.0192 | 1.04 |
| Rhodopseudomonas | 4.3523 | 1 | 1 | Rhodopseudomonas_palustris | 1 | 1 | 1164/5744041 | 0.0203 | 1.04 |
| Roseomonas | 2.1981 | 2 | 1 | Roseomonas_mucosa | 2 | 1 | 150/4865340 | 0.0031 | 1 |
| Roseomonas | 2.1981 | 2 | 1 | Roseomonas_cervicalis | 0 | 0 | 150/5105947 | 0.0029 | 1 |
| Roseomonas | 2.1981 | 2 | 1 | Roseomonas_gilardii | 0 | 0 | 223/5030491 | 0.0044 | 1 |
| Rothia | 1.6514 | 3 | 1 | Rothia_aeria | 2 | 1 | 100/2603498 | 0.0038 | 1 |
| Rothia | 1.6514 | 3 | 1 | Rothia_mucilaginosa | 1 | 1 | 100/2264603 | 0.0044 | 1 |
| Serinicoccus | 1.1244 | 1 | 1 | Serinicoccus_chungangensis | 1 | 1 | 200/3557308 | 0.0056 | 1 |
| Serratia | 3.3844 | 3 | 1 | Serratia_ficaria | 1 | 1 | 252/5262621 | 0.0048 | 1.12 |
| Serratia | 3.3844 | 3 | 1 | Serratia_grimesii | 0 | 0 | 43/5134258 | 0.0008 | 1 |
| Serratia | 3.3844 | 3 | 1 | Serratia_marcescens | 0 | 0 | 339/5471439 | 0.0062 | 1 |
| Serratia | 3.3844 | 3 | 1 | Serratia_plymuthica | 0 | 0 | 50/5546041 | 0.0009 | 1 |
| Serratia | 3.3844 | 3 | 1 | Serratia_proteamaculans | 0 | 0 | 50/5448853 | 0.0009 | 1 |
| Serratia | 3.3844 | 3 | 1 | Serratia_rubidaea | 0 | 0 | 93/4922834 | 0.0019 | 1 |
| Sphingopyxis | 4.1851 | 1 | 1 | Sphingopyxis_alaskensis | 1 | 1 | 685/3345170 | 0.0205 | 1 |
| Starkeya | 1.2592 | 1 | 1 | Starkeya_novella | 1 | 1 | 291/4765023 | 0.0061 | 1 |
| Thauera | 10.2308 | 2 | 1 | Thauera_MZ1T | 2 | 1 | 2171/4496212 | 0.0483 | 1.03 |
| Thermus | 0.9713 | 1 | 1 | Thermus_CCB | 0 | 0 | 50/2243772 | 0.0022 | 1 |
| Thermus | 0.9713 | 1 | 1 | Thermus_thermophilus | 0 | 0 | 50/1902595 | 0.0026 | 1 |
| Turicella | 1.392 | 2 | 1 | Turicella_otitidis | 2 | 1 | 150/2155169 | 0.007 | 1 |
| Variovorax | 13.4294 | 2 | 1 | Variovorax_paradoxus | 2 | 1 | 4463/7148516 | 0.0624 | 1.04 |
| Weissella | 0.4507 | 1 | 1 | Weissella_confusa | 1 | 1 | 51/2218694 | 0.0023 | 1 |
| - | - | - | - | Comamonadaceae_bacterium | 1 | 1 | 223/2991840 | 0.0075 | 1 |
| - | - | - | - | gamma_proteobacterium | 0 | 0 | 41/4587455 | 0.0009 | 1 |
| - | - | - | - | Methylocystis_SC2 | 0 | 0 | 50/3773444 | 0.0013 | 1 |
| - | - | - | - | Plautia_stali_symbiont | 0 | 0 | 94/4035456 | 0.0023 | 1 |
| Acidibacillus | 0.3121 | 0 | 0 | Acidibacillus_ferrooxidans | 0 | 0 | 41/3203796 | 0.0013 | 1 |
| Acidisphaera | 1.5485 | 0 | 0 | Acidisphaera_rubrifaciens | 0 | 0 | 278/3874653 | 0.0072 | 1 |
| Actibacterium | 0.2691 | 0 | 0 | Actibacterium_mucosum | 0 | 0 | 50/3715547 | 0.0013 | 1 |
| Actinokineospora | 0.2573 | 0 | 0 | Actinokineospora_bangkokensis | 0 | 0 | 42/7454023 | 0.0006 | 1 |
| Actinokineospora | 0.2573 | 0 | 0 | Actinokineospora_enzanensis | 0 | 0 | 50/8120728 | 0.0006 | 1 |
| Actinomadura | 0.7087 | 0 | 0 | Actinomadura_atramentaria | 0 | 0 | 45/6710939 | 0.0007 | 1 |
| Actinomadura | 0.7087 | 0 | 0 | Actinomadura_latina | 0 | 0 | 92/7868398 | 0.0012 | 1 |
| Actinomadura | 0.7087 | 0 | 0 | Actinomadura_macra | 0 | 0 | 42/9046901 | 0.0005 | 1 |
| Actinomadura | 0.7087 | 0 | 0 | Actinomadura_madurae | 0 | 0 | 94/10255684 | 0.0009 | 1 |
| Actinomycetospora | 0.6824 | 0 | 0 | Actinomycetospora_chiangmaiensis | 0 | 0 | 194/5861389 | 0.0033 | 1 |
| Actinoplanes | 0.408 | 0 | 0 | Actinoplanes_friuliensis | 0 | 0 | 46/9376071 | 0.0005 | 1 |
| Actinoplanes | 0.408 | 0 | 0 | Actinoplanes_globisporus | 0 | 0 | 50/10989147 | 0.0005 | 1 |
| Actinoplanes | 0.408 | 0 | 0 | Actinoplanes_missouriensis | 0 | 0 | 46/8773466 | 0.0005 | 1 |
| Actinoplanes | 0.408 | 0 | 0 | Actinoplanes_philippinensis | 0 | 0 | 50/10381810 | 0.0005 | 1 |
| Actinospica | 0.1008 | 0 | 0 | Actinospica_robiniae | 0 | 0 | 50/9918887 | 0.0005 | 1 |
| Actinotalea | 0.2504 | 0 | 0 | Actinotalea_ferrariae | 0 | 0 | 50/3993327 | 0.0013 | 1 |
| Acuticoccus | 0.7844 | 0 | 0 | Acuticoccus_yangtzensis | 0 | 0 | 193/5099489 | 0.0038 | 1 |
| Advenella | 0.6509 | 0 | 0 | Advenella_kashmirensis | 0 | 0 | 45/4365995 | 0.001 | 1 |
| Advenella | 0.6509 | 0 | 0 | Advenella_mimigardefordensis | 0 | 0 | 86/4740516 | 0.0018 | 1 |
| Aequorivita | 0.2828 | 0 | 0 | Aequorivita_viscosa | 0 | 0 | 50/3536142 | 0.0014 | 1 |
| Afifella | 0.2523 | 0 | 0 | Afifella_marina | 0 | 0 | 44/3963651 | 0.0011 | 1 |
| Afipia | 6.568 | 0 | 0 | Afipia_birgiae | 0 | 0 | 773/5334186 | 0.0145 | 1 |
| Afipia | 6.568 | 0 | 0 | Afipia_felis | 0 | 0 | 711/4203390 | 0.0169 | 1 |
| Agreia | 0.255 | 0 | 0 | Agreia_bicolorata | 0 | 0 | 50/3920909 | 0.0013 | 1 |
| Agromyces | 0.4675 | 0 | 0 | Agromyces_aureus | 0 | 0 | 50/4373124 | 0.0011 | 1 |
| Agromyces | 0.4675 | 0 | 0 | Agromyces_cerinus | 0 | 0 | 50/4187579 | 0.0012 | 1 |
| Aidingimonas | 0.2573 | 0 | 0 | Aidingimonas_halophila | 0 | 0 | 45/3886683 | 0.0012 | 1 |
| Akkermansia | 0.3754 | 0 | 0 | Akkermansia_muciniphila | 0 | 0 | 42/2664102 | 0.0016 | 1 |
| Albimonas | 0.5982 | 0 | 0 | Albimonas_donghaensis | 0 | 0 | 147/5014684 | 0.0029 | 1 |
| Alcanivorax | 0.6786 | 0 | 0 | Alcanivorax_dieselolei | 0 | 0 | 93/4928223 | 0.0019 | 1 |
| Alcanivorax | 0.6786 | 0 | 0 | Alcanivorax_hongdengensis | 0 | 0 | 50/3665796 | 0.0014 | 1 |
| Aliiroseovarius | 0.546 | 0 | 0 | Aliiroseovarius_crassostreae | 0 | 0 | 100/3663285 | 0.0027 | 1 |
| Alistipes | 0.8857 | 0 | 0 | Alistipes_indistinctus | 0 | 0 | 45/2855479 | 0.0016 | 1 |
| Alistipes | 0.8857 | 0 | 0 | Alistipes_senegalensis | 0 | 0 | 45/4017659 | 0.0011 | 1 |
| Alistipes | 0.8857 | 0 | 0 | Alistipes_timonensis | 0 | 0 | 45/3489164 | 0.0013 | 1 |
| Alkalilimnicola | 0.6105 | 0 | 0 | Alkalilimnicola_ehrlichii | 0 | 0 | 92/3275944 | 0.0028 | 1 |
| Alloactinosynnema | 0.152 | 0 | 0 | Alloactinosynnema_iranicum | 0 | 0 | 42/6579143 | 0.0006 | 1 |
| Allochromatium | 0.905 | 0 | 0 | Allochromatium_vinosum | 0 | 0 | 93/3526903 | 0.0026 | 1 |
| Allochromatium | 0.905 | 0 | 0 | Allochromatium_warmingii | 0 | 0 | 45/2959437 | 0.0015 | 1 |
| Altererythrobacter | 4.5775 | 0 | 0 | Altererythrobacter_atlanticus | 0 | 0 | 525/3386291 | 0.0155 | 1 |
| Altererythrobacter | 4.5775 | 0 | 0 | Altererythrobacter_dongtanensis | 0 | 0 | 192/3009495 | 0.0064 | 1 |
| Amantichitinum | 1.4201 | 0 | 0 | Amantichitinum_ursilacus | 0 | 0 | 342/4929321 | 0.0069 | 1 |
| Aminobacter | 0.3556 | 0 | 0 | Aminobacter_aminovorans | 0 | 0 | 97/5623946 | 0.0017 | 1 |
| Amorphus | 0.4632 | 0 | 0 | Amorphus_coralli | 0 | 0 | 92/4317655 | 0.0021 | 1 |
| Ancylobacter | 0.6659 | 0 | 0 | Ancylobacter_rudongensis | 0 | 0 | 134/4505254 | 0.003 | 1 |
| Andreprevotia | 4.3224 | 0 | 0 | Andreprevotia_lacus | 0 | 0 | 915/4627022 | 0.0198 | 1.05 |
| Antarctobacter | 0.4235 | 0 | 0 | Antarctobacter_heliothermus | 0 | 0 | 90/4723013 | 0.0019 | 1 |
| Aquamicrobium | 0.4188 | 0 | 0 | Aquamicrobium_defluvii | 0 | 0 | 87/4775173 | 0.0018 | 1 |
| Aquimixticola | 0.5729 | 0 | 0 | Aquimixticola_soesokkakensis | 0 | 0 | 100/3491057 | 0.0029 | 1 |
| Aquisalimonas | 0.9905 | 0 | 0 | Aquisalimonas_asiatica | 0 | 0 | 193/4038167 | 0.0048 | 1 |
| Arabia | 0.5921 | 0 | 0 | Arabia_massiliensis | 0 | 0 | 97/3377915 | 0.0029 | 1 |
| Archangium | 0.2402 | 0 | 0 | Archangium_gephyra | 0 | 0 | 148/12489432 | 0.0012 | 1 |
| Arenibacter | 0.2004 | 0 | 0 | Arenibacter_certesii | 0 | 0 | 50/4990571 | 0.001 | 1 |
| Arenimonas | 2.2463 | 0 | 0 | Arenimonas_malthae | 0 | 0 | 335/3116278 | 0.0108 | 1 |
| Aromatoleum | 3.957 | 0 | 0 | Aromatoleum_aromaticum | 0 | 0 | 788/4296230 | 0.0183 | 1.06 |
| Atlantibacter | 0.2224 | 0 | 0 | Atlantibacter_hermannii | 0 | 0 | 42/4495710 | 0.0009 | 1 |
| Aurantimonas | 0.4622 | 0 | 0 | Aurantimonas_manganoxydans | 0 | 0 | 93/4327024 | 0.0021 | 1 |
| Auraticoccus | 0.4483 | 0 | 0 | Auraticoccus_monumenti | 0 | 0 | 95/4461788 | 0.0021 | 1 |
| Aureimonas | 0.4826 | 0 | 0 | Aureimonas_altamirensis | 0 | 0 | 46/4191065 | 0.0011 | 1 |
| Aureimonas | 0.4826 | 0 | 0 | Aureimonas_frigidaquae | 0 | 0 | 41/4098523 | 0.001 | 1 |
| Azonexus | 3.8907 | 0 | 0 | Azonexus_hydrophilus | 0 | 0 | 564/3341331 | 0.0169 | 1.07 |
| Azorhizobium | 2.306 | 0 | 0 | Azorhizobium_caulinodans | 0 | 0 | 235/5369772 | 0.0044 | 1 |
| Azorhizobium | 2.306 | 0 | 0 | Azorhizobium_doebereinerae | 0 | 0 | 366/5818544 | 0.0063 | 1 |
| Azospirillum | 5.0888 | 0 | 0 | Azospirillum_B510 | 0 | 0 | 243/3311395 | 0.0073 | 1 |
| Azospirillum | 5.0888 | 0 | 0 | Azospirillum_brasilense | 0 | 0 | 250/3023440 | 0.0083 | 1 |
| Azospirillum | 5.0888 | 0 | 0 | Azospirillum_halopraeferens | 0 | 0 | 289/6512867 | 0.0044 | 1 |
| Azospirillum | 5.0888 | 0 | 0 | Azospirillum_lipoferum | 0 | 0 | 150/2988332 | 0.005 | 1 |
| Azotobacter | 0.7454 | 0 | 0 | Azotobacter_vinelandii | 0 | 0 | 193/5366370 | 0.0036 | 1 |
| Balneatrix | 0.2737 | 0 | 0 | Balneatrix_alpica | 0 | 0 | 47/3654048 | 0.0013 | 1 |
| Basilea | 0.5104 | 0 | 0 | Basilea_psittacipulmonis | 0 | 0 | 43/1959069 | 0.0022 | 1 |
| Bauldia | 1.1777 | 0 | 0 | Bauldia_litoralis | 0 | 0 | 281/5094828 | 0.0055 | 1 |
| Belnapia | 0.1486 | 0 | 0 | Belnapia_moabensis | 0 | 0 | 50/6729903 | 0.0007 | 1 |
| Bergeriella | 0.4296 | 0 | 0 | Bergeriella_denitrificans | 0 | 0 | 50/2327825 | 0.0021 | 1 |
| Bifidobacterium | 0.4128 | 0 | 0 | Bifidobacterium_breve | 0 | 0 | 44/2422684 | 0.0018 | 1 |
| Blastochloris | 0.5367 | 0 | 0 | Blastochloris_viridis | 0 | 0 | 100/3726627 | 0.0027 | 1 |
| Blastopirellula | 0.4502 | 0 | 0 | Blastopirellula_marina | 0 | 0 | 135/6663871 | 0.002 | 1 |
| Brucella | 0.5805 | 0 | 0 | Brucella_pinnipedialis | 0 | 0 | 50/3399268 | 0.0015 | 1 |
| Brucella | 0.5805 | 0 | 0 | Brucella_suis | 0 | 0 | 50/3493289 | 0.0014 | 1 |
| Caenispirillum | 1.2114 | 0 | 0 | Caenispirillum_salinarum | 0 | 0 | 285/4953065 | 0.0058 | 1 |
| Calditerricola | 0.4488 | 0 | 0 | Calditerricola_satsumensis | 0 | 0 | 50/2228189 | 0.0022 | 1 |
| Candidatus_Accumulibacter | 0.9884 | 0 | 0 | Candidatus_Accumulibacter_phosphatis | 0 | 0 | 246/5058518 | 0.0049 | 1 |
| Candidimonas | 1.7725 | 0 | 0 | Candidimonas_bauzanensis | 0 | 0 | 491/5641810 | 0.0087 | 1 |
| Cardiobacterium | 1.5136 | 0 | 0 | Cardiobacterium_hominis | 0 | 0 | 148/2642744 | 0.0056 | 1.29 |
| Catenulispora | 0.0955 | 0 | 0 | Catenulispora_acidiphila | 0 | 0 | 50/10467782 | 0.0005 | 1 |
| Celeribacter | 0.4988 | 0 | 0 | Celeribacter_ethanolicus | 0 | 0 | 100/4009276 | 0.0025 | 1 |
| Cellulomonas | 1.3044 | 0 | 0 | Cellulomonas_flavigena | 0 | 0 | 98/4123179 | 0.0024 | 1.45 |
| Cellulomonas | 1.3044 | 0 | 0 | Cellulomonas_gilvus | 0 | 0 | 50/3526441 | 0.0014 | 1 |
| Cellulomonas | 1.3044 | 0 | 0 | Cellulomonas_massiliensis | 0 | 0 | 44/3410572 | 0.0013 | 1 |
| Cellulosimicrobium | 0.4345 | 0 | 0 | Cellulosimicrobium_cellulans | 0 | 0 | 92/4602850 | 0.002 | 1 |
| Chelatococcus | 0.2286 | 0 | 0 | Chelatococcus_sambhunathii | 0 | 0 | 47/4374160 | 0.0011 | 1 |
| Chitinilyticum | 1.0852 | 0 | 0 | Chitinilyticum_aquatile | 0 | 0 | 194/3685817 | 0.0053 | 1 |
| Chitinimonas | 5.1911 | 0 | 0 | Chitinimonas_koreensis | 0 | 0 | 1290/5586482 | 0.0231 | 1.07 |
| Chitiniphilus | 3.8535 | 0 | 0 | Chitiniphilus_shinanonensis | 0 | 0 | 777/4152038 | 0.0187 | 1 |
| Chondromyces | 0.1727 | 0 | 0 | Chondromyces_apiculatus | 0 | 0 | 91/11579531 | 0.0008 | 1 |
| Chromohalobacter | 0.541 | 0 | 0 | Chromohalobacter_salexigens | 0 | 0 | 92/3696649 | 0.0025 | 1 |
| Chthoniobacter | 0.1274 | 0 | 0 | Chthoniobacter_flavus | 0 | 0 | 50/7849310 | 0.0006 | 1 |
| Citreicella | 0.7127 | 0 | 0 | Citreicella_marina | 0 | 0 | 181/5612702 | 0.0032 | 1 |
| Citrobacter | 0.395 | 0 | 0 | Citrobacter_amalonaticus | 0 | 0 | 50/5584358 | 0.0009 | 1 |
| Citrobacter | 0.395 | 0 | 0 | Citrobacter_sedlakii | 0 | 0 | 50/4631756 | 0.0011 | 1 |
| Citromicrobium | 3.0544 | 0 | 0 | Citromicrobium_bathyomarinum | 0 | 0 | 475/3274004 | 0.0145 | 1 |
| Cohaesibacter | 0.3745 | 0 | 0 | Cohaesibacter_marisflavi | 0 | 0 | 94/5340122 | 0.0018 | 1 |
| Collimonas | 7.6588 | 0 | 0 | Collimonas_fungivorans | 0 | 0 | 818/5186898 | 0.0158 | 1.06 |
| Collimonas | 7.6588 | 0 | 0 | Collimonas_pratensis | 0 | 0 | 1075/5730025 | 0.0188 | 1.08 |
| Collinsella | 0.4039 | 0 | 0 | Collinsella_stercoris | 0 | 0 | 50/2475769 | 0.002 | 1 |
| Conexibacter | 0.1572 | 0 | 0 | Conexibacter_woesei | 0 | 0 | 43/6359369 | 0.0007 | 1 |
| Crenobacter | 7.6933 | 0 | 0 | Crenobacter_luteus | 0 | 0 | 1042/2859625 | 0.0364 | 1 |
| Cribrihabitans | 0.7183 | 0 | 0 | Cribrihabitans_marinus | 0 | 0 | 145/4176242 | 0.0035 | 1 |
| Criibacterium | 0.4194 | 0 | 0 | Peptostreptococcaceae_bacterium_CCRI_22567 | 0 | 0 | 47/2384640 | 0.002 | 1 |
| Cronobacter | 0.9148 | 0 | 0 | Cronobacter_condimenti | 0 | 0 | 87/4347991 | 0.002 | 1 |
| Cronobacter | 0.9148 | 0 | 0 | Cronobacter_dublinensis | 0 | 0 | 45/4431067 | 0.001 | 1 |
| Cronobacter | 0.9148 | 0 | 0 | Cronobacter_muytjensii | 0 | 0 | 43/4364114 | 0.001 | 1 |
| Cryptosporangium | 0.1087 | 0 | 0 | Cryptosporangium_arvum | 0 | 0 | 50/9195993 | 0.0005 | 1 |
| Cucumibacter | 0.2693 | 0 | 0 | Cucumibacter_marinus | 0 | 0 | 47/3713871 | 0.0013 | 1 |
| Curtobacterium | 0.2517 | 0 | 0 | Curtobacterium_pusillum | 0 | 0 | 50/3973750 | 0.0013 | 1 |
| Cyanobium | 0.2992 | 0 | 0 | Cyanobium_gracile | 0 | 0 | 50/3342364 | 0.0015 | 1 |
| Cystobacter | 0.1619 | 0 | 0 | Cystobacter_fuscus | 0 | 0 | 98/12349744 | 0.0008 | 1 |
| Dactylosporangium | 0.1747 | 0 | 0 | Dactylosporangium_aurantiacum | 0 | 0 | 98/11447840 | 0.0009 | 1 |
| Dechloromonas | 5.5755 | 0 | 0 | Dechloromonas_agitata | 0 | 0 | 698/3627226 | 0.0192 | 1.14 |
| Dechloromonas | 5.5755 | 0 | 0 | Dechloromonas_aromatica | 0 | 0 | 195/4501104 | 0.0043 | 1 |
| Deefgea | 0.2694 | 0 | 0 | Deefgea_rivuli | 0 | 0 | 50/3712199 | 0.0013 | 1 |
| Desulfobulbus | 0.2596 | 0 | 0 | Desulfobulbus_propionicus | 0 | 0 | 46/3851869 | 0.0012 | 1 |
| Desulfocurvus | 0.827 | 0 | 0 | Desulfocurvus_vexinensis | 0 | 0 | 145/3627745 | 0.004 | 1 |
| Desulfomicrobium | 0.2644 | 0 | 0 | Desulfomicrobium_escambiense | 0 | 0 | 50/3782477 | 0.0013 | 1 |
| Desulfovibrio | 2.6423 | 0 | 0 | Desulfovibrio_alaskensis | 0 | 0 | 316/3730232 | 0.0085 | 1.1 |
| Desulfovibrio | 2.6423 | 0 | 0 | Desulfovibrio_gigas | 0 | 0 | 45/3693999 | 0.0012 | 1 |
| Desulfovibrio | 2.6423 | 0 | 0 | Desulfovibrio_vulgaris | 0 | 0 | 91/4040304 | 0.0023 | 1 |
| Desulfuromonas | 0.2715 | 0 | 0 | Desulfuromonas_acetexigens | 0 | 0 | 48/3683525 | 0.0013 | 1 |
| Dickeya | 1.4371 | 0 | 0 | Dickeya_dadantii | 0 | 0 | 196/4922802 | 0.004 | 1 |
| Dickeya | 1.4371 | 0 | 0 | Dickeya_paradisiaca | 0 | 0 | 50/4679450 | 0.0011 | 1 |
| Dickeya | 1.4371 | 0 | 0 | Dickeya_solani | 0 | 0 | 43/4922468 | 0.0009 | 1 |
| Dickeya | 1.4371 | 0 | 0 | Dickeya_zeae | 0 | 0 | 44/4813854 | 0.0009 | 1 |
| Dietzia | 0.5273 | 0 | 0 | Dietzia_natronolimnaea | 0 | 0 | 93/3792731 | 0.0025 | 1 |
| Dinoroseobacter | 0.2639 | 0 | 0 | Dinoroseobacter_shibae | 0 | 0 | 50/3789584 | 0.0013 | 1 |
| Dokdonella | 0.647 | 0 | 0 | Dokdonella_immobilis | 0 | 0 | 97/4636605 | 0.0021 | 1.41 |
| DuodeniBacillus | 0.4248 | 0 | 0 | Duodenibacillus_massiliensis | 0 | 0 | 48/2353898 | 0.002 | 1 |
| Dyadobacter | 0.1435 | 0 | 0 | Dyadobacter_fermentans | 0 | 0 | 50/6967790 | 0.0007 | 1 |
| Ectothiorhodospira | 0.58 | 0 | 0 | Ectothiorhodospira_haloalkaliphila | 0 | 0 | 100/3448456 | 0.0029 | 1 |
| Eggerthella | 0.3201 | 0 | 0 | Eggerthella_YY7918 | 0 | 0 | 42/3123671 | 0.0013 | 1 |
| Eisenibacter | 0.2481 | 0 | 0 | Eisenibacter_elegans | 0 | 0 | 41/4030853 | 0.001 | 1 |
| Ekhidna | 0.2365 | 0 | 0 | Ekhidna_lutea | 0 | 0 | 47/4228456 | 0.0011 | 1 |
| Elioraea | 0.6969 | 0 | 0 | Elioraea_tepidiphila | 0 | 0 | 150/4304787 | 0.0035 | 1 |
| Elizabethkingia | 0.2595 | 0 | 0 | Elizabethkingia_meningoseptica | 0 | 0 | 41/3853873 | 0.0011 | 1 |
| Enterovibrio | 0.1776 | 0 | 0 | Enterovibrio_calviensis | 0 | 0 | 50/5630265 | 0.0009 | 1 |
| Erwinia | 0.2628 | 0 | 0 | Erwinia_amylovora | 0 | 0 | 45/3805874 | 0.0012 | 1 |
| Escherichia | 0.3979 | 0 | 0 | Escherichia_albertii | 0 | 0 | 50/4701875 | 0.0011 | 1 |
| Escherichia | 0.3979 | 0 | 0 | Escherichia_coli | 0 | 0 | 50/5399183 | 0.0009 | 1 |
| Euryhalocaulis | 0.6018 | 0 | 0 | Euryhalocaulis_caribicus | 0 | 0 | 94/3323475 | 0.0028 | 1 |
| Ewingella | 0.4108 | 0 | 0 | Ewingella_americana | 0 | 0 | 96/4868720 | 0.002 | 1 |
| Ferriphaselus | 1.1172 | 0 | 0 | Ferriphaselus_amnicola | 0 | 0 | 150/2685224 | 0.0056 | 1 |
| Fibrella | 0.1476 | 0 | 0 | Fibrella_aestuarina | 0 | 0 | 50/6775444 | 0.0007 | 1 |
| Flaviramulus | 0.253 | 0 | 0 | Flaviramulus_ichthyoenteri | 0 | 0 | 44/3953270 | 0.0011 | 1 |
| Fodinicurvata | 0.2709 | 0 | 0 | Fodinicurvata_sediminis | 0 | 0 | 42/3690738 | 0.0011 | 1 |
| Fontimonas | 1.4632 | 0 | 0 | Fontimonas_thermophila | 0 | 0 | 197/2733810 | 0.0072 | 1 |
| Formosa | 0.2365 | 0 | 0 | Formosa_agariphila | 0 | 0 | 50/4228350 | 0.0012 | 1 |
| Franconibacter | 0.409 | 0 | 0 | Franconibacter_pulveris | 0 | 0 | 95/4890151 | 0.0019 | 1 |
| Frankia | 0.6332 | 0 | 0 | Frankia_EAN1pec | 0 | 0 | 92/8982042 | 0.001 | 1.89 |
| Frankia | 0.6332 | 0 | 0 | Frankia_symbiont | 0 | 0 | 48/5323186 | 0.0009 | 1 |
| Frateuria | 0.2775 | 0 | 0 | Frateuria_aurantia | 0 | 0 | 50/3603458 | 0.0014 | 1 |
| Fulvimarina | 0.2071 | 0 | 0 | Fulvimarina_manganoxydans | 0 | 0 | 42/4828117 | 0.0009 | 1 |
| Gallaecimonas | 0.2461 | 0 | 0 | Gallaecimonas_xiamenensis | 0 | 0 | 42/4062932 | 0.001 | 1 |
| Gallionella | 1.581 | 0 | 0 | Gallionella_capsiferriformans | 0 | 0 | 198/3162471 | 0.0063 | 1.24 |
| Gelidibacter | 0.2257 | 0 | 0 | Gelidibacter_mesophilus | 0 | 0 | 50/4431483 | 0.0011 | 1 |
| Geminicoccus | 0.5265 | 0 | 0 | Geminicoccus_roseus | 0 | 0 | 141/5698410 | 0.0025 | 1 |
| Gemmata | 0.2181 | 0 | 0 | Gemmata_obscuriglobus | 0 | 0 | 95/9171051 | 0.001 | 1 |
| Gemmobacter | 0.2522 | 0 | 0 | Gemmobacter_aquatilis | 0 | 0 | 50/3964368 | 0.0013 | 1 |
| GeoAlkalibacter | 0.2604 | 0 | 0 | Geoalkalibacter_ferrihydriticus | 0 | 0 | 50/3839912 | 0.0013 | 1 |
| Geobacter | 0.427 | 0 | 0 | Geobacter_FRC | 0 | 0 | 50/4304501 | 0.0012 | 1 |
| Geobacter | 0.427 | 0 | 0 | Geobacter_uraniireducens | 0 | 0 | 50/5136364 | 0.001 | 1 |
| Geodermatophilus | 0.3758 | 0 | 0 | Geodermatophilus_obscurus | 0 | 0 | 98/5322497 | 0.0018 | 1 |
| Geothrix | 0.6079 | 0 | 0 | Geothrix_fermentans | 0 | 0 | 100/3290163 | 0.003 | 1 |
| Giesbergeria | 1.4616 | 0 | 0 | Giesbergeria_anulus | 0 | 0 | 238/3420882 | 0.007 | 1 |
| Gimesia | 0.1285 | 0 | 0 | Gimesia_maris | 0 | 0 | 43/7779237 | 0.0006 | 1 |
| Gluconacetobacter | 1.1035 | 0 | 0 | Gluconacetobacter_diazotrophicus | 0 | 0 | 100/3944163 | 0.0025 | 1 |
| Gluconacetobacter | 1.1035 | 0 | 0 | Gluconacetobacter_xylinus | 0 | 0 | 46/3136818 | 0.0015 | 1 |
| Gluconacetobacter | 1.1035 | 0 | 0 | Gluconobacter_oxydans | 0 | 0 | 50/3602424 | 0.0014 | 1 |
| Glycomyces | 0.1981 | 0 | 0 | Glycomyces_arizonensis | 0 | 0 | 50/5049059 | 0.001 | 1 |
| Granulicella | 0.3924 | 0 | 0 | Acidobacterium_MP5ACTX9 | 0 | 0 | 45/4309153 | 0.001 | 1 |
| Granulicella | 0.3924 | 0 | 0 | Granulicella_mallensis | 0 | 0 | 50/6237577 | 0.0008 | 1 |
| Granulicoccus | 0.2428 | 0 | 0 | Granulicoccus_phenolivorans | 0 | 0 | 50/4118453 | 0.0012 | 1 |
| Gryllotalpicola | 0.6791 | 0 | 0 | Gryllotalpicola_ginsengisoli | 0 | 0 | 100/2945204 | 0.0034 | 1 |
| Haliea | 0.2348 | 0 | 0 | Haliea_salexigens | 0 | 0 | 45/4259472 | 0.0011 | 1 |
| Halioglobus | 0.2448 | 0 | 0 | Halioglobus_japonicus | 0 | 0 | 46/4085301 | 0.0011 | 1 |
| Halobaculum | 0.3117 | 0 | 0 | Halobaculum_gomorrense | 0 | 0 | 43/3208396 | 0.0013 | 1 |
| Haloechinothrix | 0.2108 | 0 | 0 | Haloechinothrix_alba | 0 | 0 | 50/4744945 | 0.0011 | 1 |
| Halofilum | 0.5488 | 0 | 0 | Halofilum_ochraceum | 0 | 0 | 100/3644642 | 0.0027 | 1 |
| Halomonas | 0.7632 | 0 | 0 | Halomonas_elongata | 0 | 0 | 90/4061296 | 0.0022 | 1 |
| Halomonas | 0.7632 | 0 | 0 | Halomonas_stevensii | 0 | 0 | 50/3694265 | 0.0014 | 1 |
| Halotalea | 0.2279 | 0 | 0 | Halotalea_alkalilenta | 0 | 0 | 43/4387428 | 0.001 | 1 |
| Henriciella | 0.4611 | 0 | 0 | Henriciella_aquimarina | 0 | 0 | 97/4337139 | 0.0022 | 1 |
| Herbiconiux | 0.2597 | 0 | 0 | Herbiconiux_solani | 0 | 0 | 50/3849939 | 0.0013 | 1 |
| Herminiimonas | 1.7522 | 0 | 0 | Herminiimonas_arsenicoxydans | 0 | 0 | 297/3424307 | 0.0087 | 1 |
| Hoeflea | 0.4234 | 0 | 0 | Hoeflea_olei | 0 | 0 | 95/4724102 | 0.002 | 1 |
| Holophaga | 0.2423 | 0 | 0 | Holophaga_foetida | 0 | 0 | 50/4127257 | 0.0012 | 1 |
| Humibacter | 0.8123 | 0 | 0 | Humibacter_albus | 0 | 0 | 139/3693265 | 0.0038 | 1 |
| Hyalangium | 0.1788 | 0 | 0 | Hyalangium_minutum | 0 | 0 | 87/11186613 | 0.0008 | 1 |
| Hydrocarboniphaga | 1.5786 | 0 | 0 | Hydrocarboniphaga_daqingensis | 0 | 0 | 234/3800798 | 0.0062 | 1.18 |
| Hyphomonas | 0.2699 | 0 | 0 | Hyphomonas_neptunium | 0 | 0 | 50/3705021 | 0.0013 | 1 |
| Ilumatobacter | 0.4141 | 0 | 0 | Acidimicrobidae_bacterium | 0 | 0 | 94/4830181 | 0.0019 | 1 |
| Imhoffiella | 1.4681 | 0 | 0 | Imhoffiella_purpurea | 0 | 0 | 288/4768165 | 0.006 | 1.15 |
| Inquilinus | 1.079 | 0 | 0 | Inquilinus_limosus | 0 | 0 | 336/7414344 | 0.0045 | 1.15 |
| Insolitispirillum | 0.6468 | 0 | 0 | Insolitispirillum_peregrinum | 0 | 0 | 143/4638276 | 0.0031 | 1 |
| Jiangella | 0.3888 | 0 | 0 | Jiangella_alkaliphila | 0 | 0 | 132/7716600 | 0.0017 | 1 |
| Joostella | 0.2218 | 0 | 0 | Joostella_marina | 0 | 0 | 50/4508253 | 0.0011 | 1 |
| Kaistia | 0.4133 | 0 | 0 | Kaistia_adipata | 0 | 0 | 97/4839313 | 0.002 | 1 |
| Kerstersia | 1.7792 | 0 | 0 | Kerstersia_gyiorum | 0 | 0 | 282/3934293 | 0.0072 | 1.16 |
| Ketogulonicigenium | 0.3297 | 0 | 0 | Ketogulonicigenium_vulgare | 0 | 0 | 50/3032608 | 0.0016 | 1 |
| Kingella | 0.4503 | 0 | 0 | Kingella_denitrificans | 0 | 0 | 50/2220565 | 0.0023 | 1 |
| Kitasatospora | 0.1139 | 0 | 0 | Kitasatospora_setae | 0 | 0 | 43/8783278 | 0.0005 | 1 |
| Kluyvera | 0.1982 | 0 | 0 | Kluyvera_cryocrescens | 0 | 0 | 46/5045663 | 0.0009 | 1 |
| Komagataeibacter | 0.275 | 0 | 0 | Komagataeibacter_hansenii | 0 | 0 | 43/3636659 | 0.0012 | 1 |
| Kordiimonas | 0.245 | 0 | 0 | Kordiimonas_gwangyangensis | 0 | 0 | 50/4082365 | 0.0012 | 1 |
| Krasilnikoviella | 0.4127 | 0 | 0 | Krasilnikoviella_flava | 0 | 0 | 96/4846106 | 0.002 | 1 |
| Kribbia | 0.9099 | 0 | 0 | Kribbia_dieselivorans | 0 | 0 | 146/3296992 | 0.0044 | 1 |
| Lacunisphaera | 0.2381 | 0 | 0 | Lacunisphaera_limnophila | 0 | 0 | 50/4199284 | 0.0012 | 1 |
| Lamprocystis | 0.4341 | 0 | 0 | Lamprocystis_purpurea | 0 | 0 | 136/6910865 | 0.002 | 1 |
| Lampropedia | 5.3809 | 0 | 0 | Lampropedia_cohaerens | 0 | 0 | 801/3159322 | 0.0254 | 1 |
| Laribacter | 3.7863 | 0 | 0 | Laribacter_hongkongensis | 0 | 0 | 536/3169329 | 0.0169 | 1.08 |
| Lawsonella | 0.5222 | 0 | 0 | Lawsonella_clevelandensis | 0 | 0 | 50/1915154 | 0.0026 | 1 |
| Legionella | 0.3432 | 0 | 0 | Legionella_lansingensis | 0 | 0 | 50/2913675 | 0.0017 | 1 |
| Leifsonia | 0.7445 | 0 | 0 | Leifsonia_xyli | 0 | 0 | 96/2686418 | 0.0036 | 1 |
| Leisingera | 0.4567 | 0 | 0 | Leisingera_daeponensis | 0 | 0 | 50/4642636 | 0.0011 | 1 |
| Leisingera | 0.4567 | 0 | 0 | Leisingera_methylohalidivorans | 0 | 0 | 50/4144900 | 0.0012 | 1 |
| Lelliottia | 0.2166 | 0 | 0 | Lelliottia_amnigena | 0 | 0 | 50/4616371 | 0.0011 | 1 |
| Leucothrix | 0.1928 | 0 | 0 | Leucothrix_mucor | 0 | 0 | 50/5185684 | 0.001 | 1 |
| Levilinea | 0.2364 | 0 | 0 | Levilinea_saccharolytica | 0 | 0 | 50/4230718 | 0.0012 | 1 |
| Limimonas | 0.987 | 0 | 0 | Limimonas_halophila | 0 | 0 | 145/3039611 | 0.0048 | 1 |
| Litorimicrobium | 0.2488 | 0 | 0 | Litorimicrobium_taeanense | 0 | 0 | 41/4019874 | 0.001 | 1 |
| Loktanella | 0.9509 | 0 | 0 | Loktanella_atrilutea | 0 | 0 | 126/4206474 | 0.003 | 1.34 |
| Longispora | 0.2932 | 0 | 0 | Longispora_albida | 0 | 0 | 91/6821501 | 0.0013 | 1 |
| Lonsdalea | 0.2646 | 0 | 0 | Lonsdalea_quercina | 0 | 0 | 45/3779599 | 0.0012 | 1 |
| Luteibacter | 1.4689 | 0 | 0 | Luteibacter_rhizovicinus | 0 | 0 | 285/4765486 | 0.006 | 1.16 |
| Luteimonas | 1.7552 | 0 | 0 | Luteimonas_abyssi | 0 | 0 | 341/3988241 | 0.0086 | 1 |
| Luteipulveratus | 0.224 | 0 | 0 | Luteipulveratus_halotolerans | 0 | 0 | 42/4464306 | 0.0009 | 1 |
| Lutibaculum | 0.2325 | 0 | 0 | Lutibaculum_baratangense | 0 | 0 | 50/4301206 | 0.0012 | 1 |
| Lutimaribacter | 0.5301 | 0 | 0 | Lutimaribacter_saemankumensis | 0 | 0 | 94/3772993 | 0.0025 | 1 |
| Lysinibacillus | 0.2131 | 0 | 0 | Lysinibacillus_sphaericus | 0 | 0 | 47/4692801 | 0.001 | 1 |
| Magnetovibrio | 0.2747 | 0 | 0 | Magnetovibrio_blakemorei | 0 | 0 | 50/3639704 | 0.0014 | 1 |
| Maricaulis | 1.2351 | 0 | 0 | Maricaulis_maris | 0 | 0 | 100/3368780 | 0.003 | 1 |
| Maricaulis | 1.2351 | 0 | 0 | Maricaulis_salignorans | 0 | 0 | 88/3118128 | 0.0028 | 1 |
| Marichromatium | 1.0584 | 0 | 0 | Marichromatium_purpuratum | 0 | 0 | 141/3779112 | 0.0037 | 1.33 |
| Marinibacterium | 0.6501 | 0 | 0 | Marinibacterium_profundimaris | 0 | 0 | 200/6152792 | 0.0033 | 1 |
| Marinobacterium | 0.2547 | 0 | 0 | Marinobacterium_georgiense | 0 | 0 | 43/3925761 | 0.0011 | 1 |
| Marinovum | 0.5366 | 0 | 0 | Marinovum_algicola | 0 | 0 | 91/3727131 | 0.0024 | 1 |
| Mariprofundus | 0.6683 | 0 | 0 | Mariprofundus_ferrooxydans | 0 | 0 | 82/2992760 | 0.0027 | 1 |
| Marivita | 0.2168 | 0 | 0 | Marivita_cryptomonadis | 0 | 0 | 45/4611521 | 0.001 | 1 |
| Martelella | 0.8369 | 0 | 0 | Martelella_endophytica | 0 | 0 | 98/4817335 | 0.002 | 1.47 |
| Martelella | 0.8369 | 0 | 0 | Martelella_mediterranea | 0 | 0 | 42/4671477 | 0.0009 | 1 |
| Mastigocladus | 0.9344 | 0 | 0 | Mastigocladus_laminosus | 0 | 0 | 379/8561912 | 0.0044 | 1 |
| Mastigocoleus | 0.063 | 0 | 0 | Mastigocoleus_testarum | 0 | 0 | 41/15876877 | 0.0003 | 1 |
| Metallibacterium | 0.3004 | 0 | 0 | Metallibacterium_scheffleri | 0 | 0 | 50/3328497 | 0.0015 | 1 |
| Methylacidiphilum | 0.4372 | 0 | 0 | Methylacidiphilum_infernorum | 0 | 0 | 41/2287145 | 0.0018 | 1 |
| Methylobacillus | 4.3749 | 0 | 0 | Methylobacillus_flagellatus | 0 | 0 | 650/2971517 | 0.0219 | 1 |
| Methylobrevis | 2.2809 | 0 | 0 | Methylobrevis_pamukkalensis | 0 | 0 | 489/4384163 | 0.0112 | 1 |
| Methylocapsa | 0.2437 | 0 | 0 | Methylocapsa_acidiphila | 0 | 0 | 50/4103443 | 0.0012 | 1 |
| Methyloceanibacter | 0.5839 | 0 | 0 | Methyloceanibacter_caenitepidi | 0 | 0 | 100/3424964 | 0.0029 | 1 |
| Methylocella | 0.4645 | 0 | 0 | Methylocella_silvestris | 0 | 0 | 85/4305430 | 0.002 | 1 |
| Methylococcus | 0.9078 | 0 | 0 | Methylococcus_capsulatus | 0 | 0 | 150/3304561 | 0.0045 | 1 |
| Methyloferula | 0.7083 | 0 | 0 | Methyloferula_stellata | 0 | 0 | 139/4235448 | 0.0033 | 1 |
| Methylogaea | 0.2698 | 0 | 0 | Methylogaea_oryzae | 0 | 0 | 50/3706144 | 0.0013 | 1 |
| Methyloligella | 0.6266 | 0 | 0 | Methyloligella_halotolerans | 0 | 0 | 91/3191878 | 0.0029 | 1 |
| Methylosinus | 0.6654 | 0 | 0 | Methylosinus_trichosporium | 0 | 0 | 143/4508832 | 0.0032 | 1 |
| Methylotenera | 0.7193 | 0 | 0 | Methylotenera_301 | 0 | 0 | 50/3059871 | 0.0016 | 1 |
| Methylotenera | 0.7193 | 0 | 0 | Methylotenera_mobilis | 0 | 0 | 50/2547570 | 0.002 | 1 |
| Methylovorus | 0.3494 | 0 | 0 | Methylovorus_MP688 | 0 | 0 | 50/2862391 | 0.0017 | 1 |
| Microbispora | 0.1128 | 0 | 0 | Microbispora_rosea | 0 | 0 | 42/8867189 | 0.0005 | 1 |
| Micromonospora | 0.7093 | 0 | 0 | Micromonospora_aurantiaca | 0 | 0 | 50/7025559 | 0.0007 | 1 |
| Micromonospora | 0.7093 | 0 | 0 | Micromonospora_auratinigra | 0 | 0 | 42/6758600 | 0.0006 | 1 |
| Micromonospora | 0.7093 | 0 | 0 | Micromonospora_avicenniae | 0 | 0 | 96/6823918 | 0.0014 | 1 |
| Micromonospora | 0.7093 | 0 | 0 | Micromonospora_carbonacea | 0 | 0 | 50/7942428 | 0.0006 | 1 |
| Microtetraspora | 0.2156 | 0 | 0 | Microtetraspora_glauca | 0 | 0 | 92/9276364 | 0.001 | 1 |
| Millionella | 0.3462 | 0 | 0 | Millionella_massiliensis | 0 | 0 | 50/2888830 | 0.0017 | 1 |
| Mizugakiibacter | 4.4832 | 0 | 0 | Mizugakiibacter_sediminis | 0 | 0 | 691/3122786 | 0.0221 | 1 |
| Monashia | 0.2355 | 0 | 0 | Monashia_flava | 0 | 0 | 50/4245965 | 0.0012 | 1 |
| Mycobacterium_tuberculosis_complex | 0.2209 | 0 | 0 | Mycobacterium_canettii | 0 | 0 | 50/4525948 | 0.0011 | 1 |
| Myxococcus | 0.4265 | 0 | 0 | Myxococcus_fulvus | 0 | 0 | 44/9003593 | 0.0005 | 1 |
| Myxococcus | 0.4265 | 0 | 0 | Myxococcus_stipitatus | 0 | 0 | 50/10350586 | 0.0005 | 1 |
| Myxococcus | 0.4265 | 0 | 0 | Myxococcus_xanthus | 0 | 0 | 95/9139763 | 0.001 | 1 |
| Nitrosomonas | 0.3144 | 0 | 0 | Nitrosomonas_AL212 | 0 | 0 | 47/3180526 | 0.0015 | 1 |
| Nitrosospira | 0.314 | 0 | 0 | Nitrosospira_multiformis | 0 | 0 | 50/3184243 | 0.0016 | 1 |
| Oceanimonas | 0.2845 | 0 | 0 | Oceanimonas_GK1 | 0 | 0 | 50/3514537 | 0.0014 | 1 |
| Oceanobacillus | 0.2782 | 0 | 0 | Oceanobacillus_massiliensis | 0 | 0 | 50/3594051 | 0.0014 | 1 |
| Oerskovia | 0.2482 | 0 | 0 | Oerskovia_turbata | 0 | 0 | 45/4029396 | 0.0011 | 1 |
| Oligotropha | 3.4707 | 0 | 0 | Oligotropha_carboxidovorans | 0 | 0 | 633/3745629 | 0.0169 | 1 |
| Opitutus | 0.1679 | 0 | 0 | Opitutus_terrae | 0 | 0 | 41/5957605 | 0.0007 | 1 |
| Pannonibacter | 0.564 | 0 | 0 | Pannonibacter_phragmitetus | 0 | 0 | 146/5318696 | 0.0027 | 1 |
| Pasteurella | 0.3425 | 0 | 0 | Pasteurella_testudinis | 0 | 0 | 50/2919413 | 0.0017 | 1 |
| Pelagibacterium | 0.507 | 0 | 0 | Pelagibacterium_halotolerans | 0 | 0 | 93/3944837 | 0.0024 | 1 |
| Pelistega | 0.399 | 0 | 0 | Pelistega_indica | 0 | 0 | 43/2506372 | 0.0017 | 1 |
| Pelotomaculum | 0.3305 | 0 | 0 | Pelotomaculum_thermopropionicum | 0 | 0 | 42/3025375 | 0.0014 | 1 |
| Phaeobacter | 0.2617 | 0 | 0 | Phaeobacter_gallaeciensis | 0 | 0 | 46/3821831 | 0.0012 | 1 |
| Phenylobacterium | 2.0019 | 0 | 0 | Phenylobacterium_zucineum | 0 | 0 | 377/3996255 | 0.0094 | 1 |
| Photobacterium | 0.1581 | 0 | 0 | Photobacterium_profundum | 0 | 0 | 50/6323247 | 0.0008 | 1 |
| Phycisphaera | 0.2629 | 0 | 0 | Phycisphaera_mikurensis | 0 | 0 | 50/3803225 | 0.0013 | 1 |
| Plesiomonas | 0.5872 | 0 | 0 | Plesiomonas_shigelloides | 0 | 0 | 52/3405979 | 0.0015 | 1.92 |
| Pluralibacter | 0.7798 | 0 | 0 | Enterobacter_lignolyticus | 0 | 0 | 92/4814049 | 0.0019 | 1 |
| Pluralibacter | 0.7798 | 0 | 0 | Pluralibacter_gergoviae | 0 | 0 | 89/5489680 | 0.0016 | 1 |
| Polymorphum | 0.4302 | 0 | 0 | Polymorphum_gilvum | 0 | 0 | 92/4649365 | 0.002 | 1 |
| Pseudogulbenkiania | 5.0773 | 0 | 0 | Pseudogulbenkiania_NH8B | 0 | 0 | 1004/4332995 | 0.0232 | 1.03 |
| Rhizobium | 0.9817 | 0 | 0 | Rhizobium_etli | 0 | 0 | 91/4598466 | 0.002 | 1 |
| Rhizobium | 0.9817 | 0 | 0 | Rhizobium_IRBG74 | 0 | 0 | 50/2844565 | 0.0018 | 1 |
| Rhizobium | 0.9817 | 0 | 0 | Rhizobium_leguminosarum | 0 | 0 | 50/5119898 | 0.001 | 1 |
| Rhodomicrobium | 0.2491 | 0 | 0 | Rhodomicrobium_vannielii | 0 | 0 | 50/4014469 | 0.0012 | 1 |
| Rhodospirillum | 1.8368 | 0 | 0 | Rhodospirillum_centenum | 0 | 0 | 337/4355543 | 0.0077 | 1 |
| Rhodospirillum | 1.8368 | 0 | 0 | Rhodospirillum_rubrum | 0 | 0 | 42/4352825 | 0.001 | 1 |
| Ruegeria | 1.0424 | 0 | 0 | Ruegeria_pomeroyi | 0 | 0 | 138/4109437 | 0.0034 | 1 |
| Ruegeria | 1.0424 | 0 | 0 | Ruegeria_TM1040 | 0 | 0 | 44/3200938 | 0.0014 | 1 |
| Salmonella | 0.2027 | 0 | 0 | Salmonella_typhimurium | 0 | 0 | 43/4933631 | 0.0009 | 1 |
| Sanguibacter | 0.2351 | 0 | 0 | Sanguibacter_keddieii | 0 | 0 | 42/4253413 | 0.001 | 1 |
| Segniliparus | 0.2787 | 0 | 0 | Segniliparus_rugosus | 0 | 0 | 50/3587639 | 0.0014 | 1 |
| Shimwellia | 0.2405 | 0 | 0 | Shimwellia_blattae | 0 | 0 | 43/4158725 | 0.001 | 1 |
| Sideroxydans | 1.3317 | 0 | 0 | Sideroxydans_lithotrophicus | 0 | 0 | 182/3003656 | 0.0061 | 1 |
| Simiduia | 0.4651 | 0 | 0 | Simiduia_agarivorans | 0 | 0 | 90/4300227 | 0.0021 | 1 |
| Sinorhizobium | 0.4632 | 0 | 0 | Sinorhizobium_fredii | 0 | 0 | 135/6476459 | 0.0021 | 1 |
| Sodalis | 0.2397 | 0 | 0 | Sodalis_glossinidius | 0 | 0 | 43/4171146 | 0.001 | 1 |
| Sorangium | 0.3382 | 0 | 0 | Sorangium_cellulosum | 0 | 0 | 186/14782125 | 0.0013 | 1.32 |
| Sphingobacterium | 0.1694 | 0 | 0 | Sphingobacterium_thalpophilum | 0 | 0 | 45/5904641 | 0.0008 | 1 |
| Spiribacter | 0.519 | 0 | 0 | Spiribacter_UAH | 0 | 0 | 46/1926631 | 0.0024 | 1 |
| Spirosoma | 0.1238 | 0 | 0 | Spirosoma_linguale | 0 | 0 | 43/8078757 | 0.0005 | 1 |
| Stigmatella | 0.1949 | 0 | 0 | Stigmatella_aurantiaca | 0 | 0 | 100/10260756 | 0.001 | 1 |
| Streptomyces | 1.0678 | 0 | 0 | Streptomyces_albus | 0 | 0 | 94/8384669 | 0.0011 | 1 |
| Streptomyces | 1.0678 | 0 | 0 | Streptomyces_bingchenggensis | 0 | 0 | 100/11936683 | 0.0008 | 1 |
| Streptomyces | 1.0678 | 0 | 0 | Streptomyces_cattleya | 0 | 0 | 50/6283062 | 0.0008 | 1 |
| Streptomyces | 1.0678 | 0 | 0 | Streptomyces_coelicolor | 0 | 0 | 50/8667507 | 0.0006 | 1 |
| Streptomyces | 1.0678 | 0 | 0 | Streptomyces_collinus | 0 | 0 | 42/8272925 | 0.0005 | 1 |
| Streptomyces | 1.0678 | 0 | 0 | Streptomyces_scabiei | 0 | 0 | 42/10148695 | 0.0004 | 1 |
| Streptomyces | 1.0678 | 0 | 0 | Streptomyces_xiamenensis | 0 | 0 | 50/5961401 | 0.0008 | 1 |
| Sulfuricella | 0.6389 | 0 | 0 | Sulfuricella_denitrificans | 0 | 0 | 92/3130594 | 0.0029 | 1 |
| Sutterella | 2.0161 | 0 | 0 | Sutterella_parvirubra | 0 | 0 | 183/2378769 | 0.0077 | 1 |
| Sutterella | 2.0161 | 0 | 0 | Sutterella_wadsworthensis | 0 | 0 | 46/2988348 | 0.0015 | 1 |
| Taylorella | 0.6103 | 0 | 0 | Taylorella_asinigenitalis | 0 | 0 | 50/1638559 | 0.0031 | 1 |
| Thermomonospora | 0.532 | 0 | 0 | Thermomonospora_curvata | 0 | 0 | 142/5639016 | 0.0025 | 1 |
| Thioalkalivibrio | 2.5958 | 0 | 0 | Thioalkalivibrio_K90mix | 0 | 0 | 50/2744800 | 0.0018 | 1 |
| Thioalkalivibrio | 2.5958 | 0 | 0 | Thioalkalivibrio_nitratireducens | 0 | 0 | 94/4002352 | 0.0023 | 1 |
| Thioalkalivibrio | 2.5958 | 0 | 0 | Thioalkalivibrio_sulfidophilus | 0 | 0 | 288/3464554 | 0.0083 | 1 |
| Thiobacillus | 4.8113 | 0 | 0 | Thiobacillus_denitrificans | 0 | 0 | 529/2909809 | 0.0182 | 1.24 |
| Thiocystis | 0.1993 | 0 | 0 | Thiocystis_violascens | 0 | 0 | 50/5017071 | 0.001 | 1 |
| Thioflavicoccus | 0.494 | 0 | 0 | Thioflavicoccus_mobilis | 0 | 0 | 96/4048921 | 0.0024 | 1 |
| Thiomonas | 4.7357 | 0 | 0 | Thiomonas_3As | 0 | 0 | 483/3738778 | 0.0129 | 1 |
| Thiomonas | 4.7357 | 0 | 0 | Thiomonas_intermedia | 0 | 0 | 336/3396378 | 0.0099 | 1 |
| Tistrella | 0.7654 | 0 | 0 | Tistrella_mobilis | 0 | 0 | 143/3919492 | 0.0036 | 1 |
| Treponema | 1.1502 | 0 | 0 | Treponema_denticola | 0 | 0 | 41/2843201 | 0.0014 | 1 |
| Treponema | 1.1502 | 0 | 0 | Treponema_lecithinolyticum | 0 | 0 | 50/2341085 | 0.0021 | 1 |
| Treponema | 1.1502 | 0 | 0 | Treponema_vincentii | 0 | 0 | 50/2693513 | 0.0019 | 1 |
| Truepera | 0.3067 | 0 | 0 | Truepera_radiovictrix | 0 | 0 | 45/3260398 | 0.0014 | 1 |
| Trueperella | 0.4882 | 0 | 0 | Trueperella_bernardiae | 0 | 0 | 50/2048535 | 0.0024 | 1 |
| Tsukamurella | 0.8363 | 0 | 0 | Tsukamurella_pulmonis | 0 | 0 | 176/4783074 | 0.0037 | 1 |
| Verrucosispora | 0.2997 | 0 | 0 | Verrucosispora_maris | 0 | 0 | 90/6673976 | 0.0013 | 1 |
| Vibrio | 0.3905 | 0 | 0 | Vibrio_EJY3 | 0 | 0 | 48/5452646 | 0.0009 | 1 |
| Vibrio | 0.3905 | 0 | 0 | Vibrio_fluvialis | 0 | 0 | 50/4827734 | 0.001 | 1 |
| Xylanimonas | 0.2672 | 0 | 0 | Xylanimonas_cellulosilytica | 0 | 0 | 45/3742776 | 0.0012 | 1 |
| Yersinia | 0.4271 | 0 | 0 | Yersinia_mollaretii | 0 | 0 | 100/4682880 | 0.0021 | 1 |

Note: Genus Re Abu: Relative abundance of Genus; SMRNG: Stringent mapped reads number of genus; SDSMRNG: Standard Stringent mapped reads number of genus; SMRN: Stringent mapped reads number; SDSMRN: Standard Stringent mapped reads number; CovRate: Coverage rate.

　　 Table 2. Microbe reads of fungi, parasite and virus detected in Case No. 2

| Genus | Genus Abs Abu | SMRNG | SDSMRNG | Species | SMRN | SDSMRN | Coverage | CovRate | Depth |
| --- | --- | --- | --- | --- | --- | --- | --- | --- | --- |
| Alternaria | 1.3325 | 41 | 18 | Alternaria_alternata | 41 | 18 | 2193/33021769 | 0.0066 | 1 |
| Sordaria | 6.5745 | 25 | 11 | Sordaria_macrospora | 25 | 11 | 11660/40002837 | 0.0291 | 1.12 |
| Malassezia | 1.9547 | 13 | 6 | Malassezia_globosa | 11 | 5 | 695/8872979 | 0.0078 | 1 |
| Aspergillus | 0.3163 | 9 | 4 | Aspergillus_sydowii | 7 | 3 | 450/34381986 | 0.0013 | 1 |
| Candida | 0.0765 | 1 | 1 | Candida_parapsilosis | 1 | 1 | 50/13078718 | 0.0004 | 1 |
| Fusarium | 0.0221 | 1 | 1 | Fusarium_proliferatum | 1 | 1 | 50/45210634 | 0.0001 | 1 |
| Hanseniaspora | 0.1135 | 1 | 1 | Hanseniaspora_uvarum | 1 | 1 | 50/8807956 | 0.0006 | 1 |
| Malassezia | 1.9547 | 13 | 6 | Malassezia_furfur | 1 | 1 | 100/15273010 | 0.0007 | 1 |
| Malassezia | 1.9547 | 13 | 6 | Malassezia_pachydermatis | 1 | 1 | 100/8129607 | 0.0012 | 1 |
| Meyerozyma | 0.0943 | 1 | 1 | Meyerozyma_guilliermondii | 1 | 1 | 50/10610034 | 0.0005 | 1 |
| Penicillium | 0.0601 | 2 | 1 | Penicillium_chrysogenum | 1 | 1 | 50/32525581 | 0.0002 | 1 |
| Penicillium | 0.0601 | 2 | 1 | Penicillium_citrinum | 1 | 1 | 50/33998688 | 0.0001 | 1 |
| Rhizopus | 0.0233 | 1 | 1 | Rhizopus_oryzae | 1 | 1 | 50/42946072 | 0.0001 | 1 |
| Allomyces | 0.0526 | 0 | 0 | Allomyces_macrogynus | 0 | 0 | 133/57061573 | 0.0002 | 1 |
| Aspergillus | 0.3163 | 9 | 4 | Aspergillus_calidoustus | 0 | 0 | 45/41103455 | 0.0001 | 1 |
| Aspergillus | 0.3163 | 9 | 4 | Aspergillus_versicolor | 0 | 0 | 50/33127310 | 0.0002 | 1 |
| Coprinopsis | 0.0276 | 0 | 0 | Coprinopsis_cinerea | 0 | 0 | 50/36193260 | 0.0001 | 1 |
| Magnaporthe | 0.0244 | 0 | 0 | Magnaporthe_oryzae | 0 | 0 | 47/40979641 | 0.0001 | 1 |
| Thermothelomyces | 0.0258 | 0 | 0 | Myceliophthora_thermophila | 0 | 0 | 50/38744276 | 0.0001 | 1 |
| Phycomyces | 0.0185 | 0 | 0 | Phycomyces_blakesleeanus | 0 | 0 | 44/53939957 | 0.0001 | 1 |
| Pythium | 0.0223 | 0 | 0 | Pythium_arrhenomanes | 0 | 0 | 45/44782335 | 0.0001 | 1 |
| Saprolegnia | 0.0159 | 0 | 0 | Saprolegnia_diclina | 0 | 0 | 41/62889682 | 0.0001 | 1 |
| Sporothrix | 0.031 | 0 | 0 | Sporothrix_schenckii | 0 | 0 | 50/32228280 | 0.0002 | 1 |
| Trichosporon | 0.187 | 0 | 0 | Trichosporon_asahii | 0 | 0 | 50/25301538 | 0.0002 | 1 |
| Trichosporon | 0.187 | 0 | 0 | Trichosporon_inkin | 0 | 0 | 42/20339708 | 0.0002 | 2.95 |
| Acanthamoeba | 1.101 | 9 | 4 | Acanthamoeba_mauritaniensis | 1 | 1 | 724/96417684 | 0.0008 | 1 |
| Hammondia | 0.0164 | 1 | 1 | Hammondia_hammondi | 1 | 1 | 50/60951907 | 0.0001 | 1 |
| Necator | 0.0347 | 2 | 1 | Necator_americanus | 2 | 1 | 362/230537098 | 0.0002 | 1.08 |
| Spirometra | 0.0044 | 2 | 1 | Spirometra_erinaceieuropaei | 2 | 1 | 250/1144239852 | 0 | 1 |
| Wuchereria | 0.013 | 1 | 1 | Wuchereria_bancrofti | 1 | 1 | 50/77159569 | 0.0001 | 1 |
| Acanthamoeba | 1.101 | 9 | 4 | Acanthamoeba_palestinensis | 0 | 0 | 1727/93383381 | 0.0018 | 1.03 |
| Acanthamoeba | 1.101 | 9 | 4 | Acanthamoeba_polyphaga | 0 | 0 | 670/109518714 | 0.0006 | 1 |
| Acanthamoeba | 1.101 | 9 | 4 | Acanthamoeba_triangularis | 0 | 0 | 1805/85627420 | 0.0021 | 1 |
| Anisakis | 0.0087 | 0 | 0 | Anisakis_simplex | 0 | 0 | 50/114426609 | 0 | 1 |
| Brugia | 0.012 | 0 | 0 | Brugia_malayi | 0 | 0 | 43/83260397 | 0.0001 | 1 |
| Clonorchis | 0.0019 | 0 | 0 | Clonorchis_sinensis | 0 | 0 | 42/536768838 | 0 | 1 |
| Entamoeba | 0.0723 | 0 | 0 | Entamoeba_dispar | 0 | 0 | 58/27672952 | 0.0002 | 1.72 |
| Fasciola | 0.0008 | 0 | 0 | Fasciola_hepatica | 0 | 0 | 41/1185539267 | 0 | 1 |
| Plasmodium | 0.257 | 0 | 0 | Plasmodium_malariae | 0 | 0 | 275/28786109 | 0.001 | 1 |
| Plasmodium | 0.257 | 0 | 0 | Plasmodium_yoelii | 0 | 0 | 42/20568926 | 0.0002 | 1 |
| Schistosoma | 0.0087 | 0 | 0 | Schistosoma_curassoni | 0 | 0 | 43/310073518 | 0 | 1 |
| Schistosoma | 0.0087 | 0 | 0 | Schistosoma_japonicum | 0 | 0 | 50/362535181 | 0 | 1 |
| Schistosoma | 0.0087 | 0 | 0 | Schistosoma_mansoni | 0 | 0 | 42/364541682 | 0 | 1 |
| Trichinella | 0.0211 | 0 | 0 | Trichinella_zimbabwensis | 0 | 0 | 50/47413163 | 0.0001 | 1 |
| Trichuris | 0.0294 | 0 | 0 | Trichuris_trichiura | 0 | 0 | 100/67961556 | 0.0001 | 1 |
| Vitrella | 0.0153 | 0 | 0 | Vitrella_brassicaformis | 0 | 0 | 46/65472090 | 0.0001 | 1 |
| - | - | - | - | Human alphaherpesvirus 3 (Varicella zoster virus) | 29527 | 13190 | 124697/124884 | 99.85 | 11.87 |

Note: Genus Re Abu: Relative abundance of Genus; SMRNG: Stringent mapped reads number of genus; SDSMRNG: Standard Stringent mapped reads number of genus; SMRN: Stringent mapped reads number; SDSMRN: Standard Stringent mapped reads number; CovRate: Coverage rate.
