# Supplementary material for: Clinical features of aseptic meningitis with varicella zoster virus infection diagnosed by next-generation sequencing: case reports
Source: BMC Infect Dis. 2020 Jun 22;20:435. doi: 10.1186/s12879-020-05155-8 (PMC7309994; doi:10.1186/s12879-020-05155-8)
Supplement: Supplementary file 4 — Additional file 4. Microbe reads of bacterium, fungi, parasite and virus detected in Case No. 3 [file 12879_2020_5155_MOESM4_ESM.docx]

**Additional file 4:** Microbe reads of bacterium, fungi, parasite and virus detected in Case No. 3

　Table 1. Microbe reads of bacterium detected in Case No. 3

| Genus | Genus Re Abu | SMRNG | SDSMRNG | Species | SMRN | SDSMRN | Coverage | CovRate | Depth |
| --- | --- | --- | --- | --- | --- | --- | --- | --- | --- |
| Moraxella | 41.31 | 1043 | 1711 | Moraxella osloensis | 1028 | 1686 | 105782/2434688 | 4.34 | 1.03 |
| Moraxella | 41.31 | 1043 | 1711 | Moraxella atlantae | 0 | 0 | 617/2289665 | 0.0269 | 1 |
| Moraxella | 41.31 | 1043 | 1711 | Moraxella lacunata | 0 | 0 | 71/2687483 | 0.0026 | 1 |
| Moraxella | 41.31 | 1043 | 1711 | Moraxella lincolnii | 0 | 0 | 57/2077203 | 0.0027 | 1 |
| Moraxella | 41.31 | 1043 | 1711 | Moraxella macacae | 0 | 0 | 148/2079299 | 0.0071 | 1 |
| Moraxella | 41.31 | 1043 | 1711 | Moraxella porci | 0 | 0 | 70/2179250 | 0.0032 | 1 |
| Burkholderia | 3.61 | 159 | 261 | Burkholderia phytofirmans | 4 | 7 | 807/8093536 | 0.01 | 1 |
| Burkholderia | 3.61 | 159 | 261 | Burkholderia cenocepacia | 3 | 5 | 1219/8531679 | 0.0143 | 1 |
| Burkholderia | 3.61 | 159 | 261 | Burkholderia ubonensis | 3 | 5 | 3589/8028779 | 0.0447 | 1 |
| Burkholderia | 3.61 | 159 | 261 | Burkholderia glumae | 2 | 3 | 677/6733840 | 0.0101 | 1 |
| Burkholderia | 3.61 | 159 | 261 | Burkholderia stabilis | 2 | 3 | 1180/8527967 | 0.0138 | 1 |
| Burkholderia | 3.61 | 159 | 261 | Burkholderia CCGE1002 | 1 | 2 | 411/7395722 | 0.0056 | 1 |
| Burkholderia | 3.61 | 159 | 261 | Burkholderia dolosa | 1 | 2 | 1220/6409115 | 0.019 | 1 |
| Burkholderia | 3.61 | 159 | 261 | Burkholderia latens | 1 | 2 | 1126/6569017 | 0.0171 | 1 |
| Burkholderia | 3.61 | 159 | 261 | Burkholderia multivorans | 1 | 2 | 1390/7281887 | 0.0191 | 1 |
| Burkholderia | 3.61 | 159 | 261 | Burkholderia ambifaria | 0 | 0 | 1241/7484986 | 0.0166 | 1 |
| Burkholderia | 3.61 | 159 | 261 | Burkholderia anthina | 0 | 0 | 981/7273081 | 0.0135 | 1 |
| Burkholderia | 3.61 | 159 | 261 | Burkholderia CCGE1001 | 0 | 0 | 250/6833751 | 0.0037 | 1 |
| Burkholderia | 3.61 | 159 | 261 | Burkholderia CCGE1003 | 0 | 0 | 107/7043595 | 0.0015 | 1 |
| Burkholderia | 3.61 | 159 | 261 | Burkholderia cepacia | 0 | 0 | 707/8396158 | 0.0084 | 1.04 |
| Burkholderia | 3.61 | 159 | 261 | Burkholderia contaminans | 0 | 0 | 547/8509269 | 0.0064 | 1 |
| Burkholderia | 3.61 | 159 | 261 | Burkholderia diffusa | 0 | 0 | 1133/6857853 | 0.0165 | 1 |
| Burkholderia | 3.61 | 159 | 261 | Burkholderia gladioli | 0 | 0 | 523/8114449 | 0.0064 | 1 |
| Burkholderia | 3.61 | 159 | 261 | Burkholderia KJ006 | 0 | 0 | 369/6584551 | 0.0056 | 1 |
| Burkholderia | 3.61 | 159 | 261 | Burkholderia lata | 0 | 0 | 848/8676277 | 0.0098 | 1 |
| Burkholderia | 3.61 | 159 | 261 | Burkholderia mallei | 0 | 0 | 251/5913144 | 0.0042 | 1 |
| Burkholderia | 3.61 | 159 | 261 | Burkholderia metallica | 0 | 0 | 1030/7424260 | 0.0139 | 1 |
| Burkholderia | 3.61 | 159 | 261 | Burkholderia oklahomensis | 0 | 0 | 1288/7313683 | 0.0176 | 1 |
| Burkholderia | 3.61 | 159 | 261 | Burkholderia phenoliruptrix | 0 | 0 | 159/6865712 | 0.0023 | 1 |
| Burkholderia | 3.61 | 159 | 261 | Burkholderia phymatum | 0 | 0 | 223/6176561 | 0.0036 | 1 |
| Burkholderia | 3.61 | 159 | 261 | Burkholderia pseudomallei | 0 | 0 | 447/7446579 | 0.006 | 1 |
| Burkholderia | 3.61 | 159 | 261 | Burkholderia pyrrocinia | 0 | 0 | 1019/7847882 | 0.013 | 1 |
| Burkholderia | 3.61 | 159 | 261 | Burkholderia RPE64 | 0 | 0 | 198/5379596 | 0.0037 | 1 |
| Burkholderia | 3.61 | 159 | 261 | Burkholderia thailandensis | 0 | 0 | 482/6739510 | 0.0072 | 1 |
| Burkholderia | 3.61 | 159 | 261 | Burkholderia vietnamiensis | 0 | 0 | 647/6827896 | 0.0095 | 1 |
| Burkholderia | 3.61 | 159 | 261 | Burkholderia xenovorans | 0 | 0 | 723/9731138 | 0.0074 | 1 |
| Burkholderia | 3.61 | 159 | 261 | Burkholderia YI23 | 0 | 0 | 179/6473869 | 0.0028 | 1 |
| Propionibacterium | 4.06 | 151 | 248 | Propionibacterium acnes | 143 | 235 | 11216/2560282 | 0.4381 | 1 |
| Propionibacterium | 4.06 | 151 | 248 | Propionibacterium humerusii | 5 | 8 | 315/2644116 | 0.0119 | 1 |
| Acinetobacter | 3.42 | 149 | 244 | Acinetobacter johnsonii | 119 | 195 | 10430/3509795 | 0.2972 | 1 |
| Acinetobacter | 3.42 | 149 | 244 | Acinetobacter bohemicus | 2 | 3 | 182/3729387 | 0.0049 | 1 |
| Acinetobacter | 3.42 | 149 | 244 | Acinetobacter haemolyticus | 2 | 3 | 192/3715798 | 0.0052 | 1 |
| Acinetobacter | 3.42 | 149 | 244 | Acinetobacter baumannii | 1 | 2 | 44/4086879 | 0.0011 | 1 |
| Acinetobacter | 3.42 | 149 | 244 | Acinetobacter beijerinckii | 1 | 2 | 129/3563374 | 0.0036 | 1 |
| Acinetobacter | 3.42 | 149 | 244 | Acinetobacter bereziniae | 1 | 2 | 300/5026552 | 0.006 | 1 |
| Acinetobacter | 3.42 | 149 | 244 | Acinetobacter junii | 1 | 2 | 46/3782588 | 0.0012 | 1 |
| Acinetobacter | 3.42 | 149 | 244 | Acinetobacter lwoffii | 1 | 2 | 542/2970206 | 0.0182 | 1 |
| Acinetobacter | 3.42 | 149 | 244 | Acinetobacter soli | 1 | 2 | 75/3463526 | 0.0022 | 1 |
| Acinetobacter | 3.42 | 149 | 244 | Acinetobacter ursingii | 1 | 2 | 165/3825524 | 0.0043 | 1 |
| Acinetobacter | 3.42 | 149 | 244 | Acinetobacter bouvetii | 0 | 0 | 124/3388872 | 0.0037 | 1 |
| Acinetobacter | 3.42 | 149 | 244 | Acinetobacter guillouiae | 0 | 0 | 49/4905802 | 0.001 | 1 |
| Acinetobacter | 3.42 | 149 | 244 | Acinetobacter indicus | 0 | 0 | 75/3157380 | 0.0024 | 1 |
| Acinetobacter | 3.42 | 149 | 244 | Acinetobacter kookii | 0 | 0 | 143/3085611 | 0.0046 | 1 |
| Acinetobacter | 3.42 | 149 | 244 | Acinetobacter larvae | 0 | 0 | 52/3741098 | 0.0014 | 1 |
| Acinetobacter | 3.42 | 149 | 244 | Acinetobacter nectaris | 0 | 0 | 52/2669218 | 0.0019 | 1 |
| Acinetobacter | 3.42 | 149 | 244 | Acinetobacter parvus | 0 | 0 | 125/3606729 | 0.0035 | 1 |
| Acinetobacter | 3.42 | 149 | 244 | Acinetobacter proteolyticus | 0 | 0 | 75/4375000 | 0.0017 | 1 |
| Acinetobacter | 3.42 | 149 | 244 | Acinetobacter schindleri | 0 | 0 | 64/3415006 | 0.0019 | 1 |
| Sphingomonas | 3.72 | 144 | 236 | Sphingomonas melonis | 82 | 135 | 6927/4156476 | 0.1667 | 1 |
| Sphingomonas | 3.72 | 144 | 236 | Sphingomonas parapaucimobilis | 39 | 64 | 5323/3995782 | 0.1332 | 1.01 |
| Sphingomonas | 3.72 | 144 | 236 | Sphingomonas echinoides | 13 | 21 | 2283/4264986 | 0.0535 | 1 |
| Sphingomonas | 3.72 | 144 | 236 | Sphingomonas adhaesiva | 4 | 7 | 1264/4026130 | 0.0314 | 1 |
| Sphingomonas | 3.72 | 144 | 236 | Sphingomonas MM | 1 | 2 | 326/4054833 | 0.008 | 1 |
| Sphingomonas | 3.72 | 144 | 236 | Sphingomonas wittichii | 1 | 2 | 488/5382261 | 0.0091 | 1 |
| Sphingomonas | 3.72 | 144 | 236 | Sphingomonas paucimobilis | 0 | 0 | 251/4874985 | 0.0051 | 1 |
| Ralstonia | 2.25 | 104 | 171 | Ralstonia insidiosa | 79 | 130 | 11717/5808308 | 0.2017 | 1 |
| Ralstonia | 2.25 | 104 | 171 | Ralstonia mannitolilytica | 1 | 2 | 1637/4881769 | 0.0335 | 1 |
| Ralstonia | 2.25 | 104 | 171 | Ralstonia solanacearum | 1 | 2 | 457/3984240 | 0.0115 | 1 |
| Ralstonia | 2.25 | 104 | 171 | Ralstonia pickettii | 0 | 0 | 333/8125850 | 0.0041 | 1 |
| Staphylococcus | 2.64 | 73 | 120 | Staphylococcus hominis | 15 | 25 | 1176/1140916 | 0.1031 | 1 |
| Staphylococcus | 2.64 | 73 | 120 | Staphylococcus epidermidis | 13 | 21 | 970/2616530 | 0.0371 | 1 |
| Staphylococcus | 2.64 | 73 | 120 | Staphylococcus haemolyticus | 9 | 15 | 766/2685015 | 0.0285 | 1 |
| Staphylococcus | 2.64 | 73 | 120 | Staphylococcus massiliensis | 7 | 11 | 499/2366665 | 0.0211 | 1 |
| Staphylococcus | 2.64 | 73 | 120 | Staphylococcus warneri | 5 | 8 | 853/2486042 | 0.0343 | 1 |
| Staphylococcus | 2.64 | 73 | 120 | Staphylococcus capitis | 3 | 5 | 494/2466594 | 0.02 | 1 |
| Staphylococcus | 2.64 | 73 | 120 | Staphylococcus cohnii | 3 | 5 | 586/2677922 | 0.0219 | 1 |
| Staphylococcus | 2.64 | 73 | 120 | Staphylococcus pasteuri | 1 | 2 | 274/2559946 | 0.0107 | 1 |
| Staphylococcus | 2.64 | 73 | 120 | Staphylococcus pettenkoferi | 1 | 2 | 121/2502346 | 0.0048 | 1 |
| Staphylococcus | 2.64 | 73 | 120 | Staphylococcus hyicus | 0 | 0 | 56/2472129 | 0.0023 | 1 |
| Staphylococcus | 2.64 | 73 | 120 | Staphylococcus lugdunensis | 0 | 0 | 121/2658366 | 0.0046 | 1 |
| Staphylococcus | 2.64 | 73 | 120 | Staphylococcus saccharolyticus | 0 | 0 | 69/2661041 | 0.0026 | 1 |
| Pseudomonas | 1.04 | 69 | 113 | Pseudomonas putida | 35 | 57 | 3262/6377271 | 0.0512 | 1 |
| Pseudomonas | 1.04 | 69 | 113 | Pseudomonas alcaligenes | 4 | 7 | 341/4406305 | 0.0077 | 1 |
| Pseudomonas | 1.04 | 69 | 113 | Pseudomonas veronii | 4 | 7 | 614/6649830 | 0.0092 | 1.06 |
| Pseudomonas | 1.04 | 69 | 113 | Pseudomonas aeruginosa | 2 | 3 | 260/6818030 | 0.0038 | 1 |
| Pseudomonas | 1.04 | 69 | 113 | Pseudomonas mendocina | 2 | 3 | 581/5434353 | 0.0107 | 1 |
| Pseudomonas | 1.04 | 69 | 113 | Pseudomonas fluorescens | 1 | 2 | 371/6136735 | 0.006 | 1 |
| Pseudomonas | 1.04 | 69 | 113 | Pseudomonas protegens | 1 | 2 | 131/6867980 | 0.0019 | 1 |
| Pseudomonas | 1.04 | 69 | 113 | Pseudomonas brassicacearum | 0 | 0 | 70/6976764 | 0.001 | 1 |
| Pseudomonas | 1.04 | 69 | 113 | Pseudomonas chlororaphis | 0 | 0 | 107/7122173 | 0.0015 | 1 |
| Pseudomonas | 1.04 | 69 | 113 | Pseudomonas entomophila | 0 | 0 | 93/5888780 | 0.0016 | 1 |
| Pseudomonas | 1.04 | 69 | 113 | Pseudomonas monteilii | 0 | 0 | 50/6000087 | 0.0008 | 1 |
| Pseudomonas | 1.04 | 69 | 113 | Pseudomonas oleovorans | 0 | 0 | 44/5006004 | 0.0009 | 1 |
| Pseudomonas | 1.04 | 69 | 113 | Pseudomonas oryzihabitans | 0 | 0 | 71/4834356 | 0.0015 | 1 |
| Pseudomonas | 1.04 | 69 | 113 | Pseudomonas resinovorans | 0 | 0 | 75/6285863 | 0.0012 | 1 |
| Pseudomonas | 1.04 | 69 | 113 | Pseudomonas stutzeri | 0 | 0 | 57/4650155 | 0.0012 | 1 |
| Pseudomonas | 1.04 | 69 | 113 | Pseudomonas syringae | 0 | 0 | 67/6665031 | 0.001 | 1 |
| Pseudomonas | 1.04 | 69 | 113 | Pseudomonas TKP | 0 | 0 | 307/7012672 | 0.0044 | 1 |
| Pseudomonas | 1.04 | 69 | 113 | Pseudomonas VLB120 | 0 | 0 | 72/5644569 | 0.0013 | 1 |
| Acidovorax | 2.37 | 68 | 112 | Acidovorax KKS102 | 63 | 103 | 10700/5196935 | 0.2059 | 1 |
| Acidovorax | 2.37 | 68 | 112 | Acidovorax JS42 | 2 | 3 | 573/4448856 | 0.0129 | 1 |
| Acidovorax | 2.37 | 68 | 112 | Acidovorax citrulli | 1 | 2 | 510/5352772 | 0.0095 | 1 |
| Acidovorax | 2.37 | 68 | 112 | Acidovorax avenae | 0 | 0 | 208/5482170 | 0.0038 | 1 |
| Acidovorax | 2.37 | 68 | 112 | Acidovorax ebreus | 0 | 0 | 90/3796573 | 0.0024 | 1 |
| Delftia | 0.78 | 62 | 102 | Delftia tsuruhatensis | 8 | 13 | 2497/7195716 | 0.0347 | 1 |
| Delftia | 0.78 | 62 | 102 | Delftia acidovorans | 4 | 7 | 2311/6767514 | 0.0341 | 1 |
| Delftia | 0.78 | 62 | 102 | Delftia Cs1 | 2 | 3 | 1110/6685842 | 0.0166 | 1 |
| Mycoplasma | 6 | 53 | 87 | Mycoplasma wenyonii | 53 | 87 | 4271/650228 | 0.6568 | 1 |
| Mycoplasma | 6 | 53 | 87 | Mycoplasma ovis | 0 | 0 | 64/702511 | 0.0091 | 1 |
| Cupriavidus | 1.13 | 48 | 79 | Cupriavidus metallidurans | 40 | 66 | 3114/3928089 | 0.0793 | 1 |
| Cupriavidus | 1.13 | 48 | 79 | Cupriavidus necator | 3 | 5 | 410/6557542 | 0.0063 | 1.02 |
| Cupriavidus | 1.13 | 48 | 79 | Cupriavidus pauculus | 2 | 3 | 406/6829059 | 0.0059 | 1 |
| Cupriavidus | 1.13 | 48 | 79 | Cupriavidus nantongensis | 1 | 2 | 331/7075719 | 0.0047 | 1 |
| Cupriavidus | 1.13 | 48 | 79 | Cupriavidus gilardii | 0 | 0 | 523/5578753 | 0.0094 | 1 |
| Cupriavidus | 1.13 | 48 | 79 | Cupriavidus taiwanensis | 0 | 0 | 57/5919322 | 0.001 | 1 |
| Corynebacterium | 1.58 | 42 | 69 | Corynebacterium callunae | 3 | 5 | 213/2839551 | 0.0075 | 1 |
| Corynebacterium | 1.58 | 42 | 69 | Corynebacterium accolens | 2 | 3 | 302/2465976 | 0.0122 | 1 |
| Corynebacterium | 1.58 | 42 | 69 | Corynebacterium pseudogenitalium | 2 | 3 | 627/2601506 | 0.0241 | 1 |
| Corynebacterium | 1.58 | 42 | 69 | Corynebacterium afermentans | 1 | 2 | 739/2345845 | 0.0315 | 1 |
| Corynebacterium | 1.58 | 42 | 69 | Corynebacterium durum | 1 | 2 | 75/2809766 | 0.0027 | 1 |
| Corynebacterium | 1.58 | 42 | 69 | Corynebacterium epidermidicanis | 1 | 2 | 116/2692072 | 0.0043 | 1 |
| Corynebacterium | 1.58 | 42 | 69 | Corynebacterium genitalium | 1 | 2 | 146/2349953 | 0.0062 | 1 |
| Corynebacterium | 1.58 | 42 | 69 | Corynebacterium glutamicum | 1 | 2 | 60/3342897 | 0.0018 | 1 |
| Corynebacterium | 1.58 | 42 | 69 | Corynebacterium lipophiloflavum | 1 | 2 | 75/2386544 | 0.0031 | 1 |
| Corynebacterium | 1.58 | 42 | 69 | Corynebacterium propinquum | 1 | 2 | 75/2553780 | 0.0029 | 1 |
| Corynebacterium | 1.58 | 42 | 69 | Corynebacterium tuberculostearicum | 1 | 2 | 576/2372621 | 0.0243 | 1 |
| Corynebacterium | 1.58 | 42 | 69 | Corynebacterium ureicelerivorans | 1 | 2 | 75/2279990 | 0.0033 | 1 |
| Corynebacterium | 1.58 | 42 | 69 | Corynebacterium xerosis | 1 | 2 | 75/2751422 | 0.0027 | 1 |
| Corynebacterium | 1.58 | 42 | 69 | Corynebacterium aurimucosum | 0 | 0 | 188/2790189 | 0.0067 | 1 |
| Corynebacterium | 1.58 | 42 | 69 | Corynebacterium efficiens | 0 | 0 | 68/3147090 | 0.0022 | 1 |
| Corynebacterium | 1.58 | 42 | 69 | Corynebacterium freneyi | 0 | 0 | 71/3043207 | 0.0023 | 1 |
| Corynebacterium | 1.58 | 42 | 69 | Corynebacterium halotolerans | 0 | 0 | 59/3135752 | 0.0019 | 1 |
| Corynebacterium | 1.58 | 42 | 69 | Corynebacterium ihumii | 0 | 0 | 512/2251322 | 0.0227 | 1 |
| Corynebacterium | 1.58 | 42 | 69 | Corynebacterium jeddahense | 0 | 0 | 75/2474555 | 0.003 | 1 |
| Corynebacterium | 1.58 | 42 | 69 | Corynebacterium minutissimum | 0 | 0 | 75/2780469 | 0.0027 | 1 |
| Corynebacterium | 1.58 | 42 | 69 | Corynebacterium oculi | 0 | 0 | 72/2413903 | 0.003 | 1 |
| Corynebacterium | 1.58 | 42 | 69 | Corynebacterium pilosum | 0 | 0 | 75/2531972 | 0.003 | 1 |
| Corynebacterium | 1.58 | 42 | 69 | Corynebacterium striatum | 0 | 0 | 75/2915083 | 0.0026 | 1 |
| Corynebacterium | 1.58 | 42 | 69 | Corynebacterium vitaeruminis | 0 | 0 | 76/2931780 | 0.0026 | 1 |
| Campylobacter | 1.42 | 36 | 59 | Campylobacter mucosalis | 36 | 59 | 1421/1752144 | 0.0811 | 1.86 |
| Methylotenera | 2.23 | 35 | 57 | Methylotenera 301 | 26 | 43 | 4192/3059871 | 0.137 | 1 |
| Methylotenera | 2.23 | 35 | 57 | Methylotenera mobilis | 7 | 11 | 2101/2547570 | 0.0825 | 1 |
| Brevundimonas | 1.38 | 33 | 54 | Brevundimonas vesicularis | 30 | 49 | 4198/3358839 | 0.125 | 1 |
| Brevundimonas | 1.38 | 33 | 54 | Brevundimonas diminuta | 0 | 0 | 98/3369386 | 0.0029 | 1 |
| Brevundimonas | 1.38 | 33 | 54 | Brevundimonas subvibrioides | 0 | 0 | 702/3445263 | 0.0204 | 1 |
| Methylobacterium | 0.73 | 32 | 52 | Methylobacterium radiotolerans | 6 | 10 | 817/6077833 | 0.0134 | 1 |
| Methylobacterium | 0.73 | 32 | 52 | Methylobacterium aquaticum | 3 | 5 | 320/5348274 | 0.006 | 1 |
| Methylobacterium | 0.73 | 32 | 52 | Methylobacterium extorquens | 3 | 5 | 877/5943768 | 0.0148 | 1 |
| Methylobacterium | 0.73 | 32 | 52 | Methylobacterium brachiatum | 2 | 3 | 433/5807713 | 0.0075 | 1 |
| Methylobacterium | 0.73 | 32 | 52 | Methylobacterium populi | 2 | 3 | 447/5800441 | 0.0077 | 1 |
| Methylobacterium | 0.73 | 32 | 52 | Methylobacterium chloromethanicum | 1 | 2 | 763/5777908 | 0.0132 | 1 |
| Methylobacterium | 0.73 | 32 | 52 | Methylobacterium 4 | 0 | 0 | 262/7659055 | 0.0034 | 1 |
| Methylobacterium | 0.73 | 32 | 52 | Methylobacterium mesophilicum | 0 | 0 | 211/6214729 | 0.0034 | 1 |
| Methylobacterium | 0.73 | 32 | 52 | Methylobacterium nodulans | 0 | 0 | 73/7772460 | 0.0009 | 1 |
| Microbacterium | 0.61 | 19 | 31 | Microbacterium laevaniformans | 11 | 18 | 1465/3000545 | 0.0488 | 1 |
| Microbacterium | 0.61 | 19 | 31 | Microbacterium hominis | 1 | 2 | 97/3506963 | 0.0028 | 1 |
| Microbacterium | 0.61 | 19 | 31 | Microbacterium oxydans | 1 | 2 | 75/4014563 | 0.0019 | 1 |
| Microbacterium | 0.61 | 19 | 31 | Microbacterium aurum | 0 | 0 | 56/3424892 | 0.0016 | 1 |
| Microbacterium | 0.61 | 19 | 31 | Microbacterium oleivorans | 0 | 0 | 68/2916866 | 0.0023 | 1 |
| Microbacterium | 0.61 | 19 | 31 | Microbacterium profundi | 0 | 0 | 55/3369467 | 0.0016 | 1 |
| Microbacterium | 0.61 | 19 | 31 | Microbacterium sediminis | 0 | 0 | 75/2760241 | 0.0027 | 1 |
| Microbacterium | 0.61 | 19 | 31 | Microbacterium testaceum | 0 | 0 | 63/3982034 | 0.0016 | 1 |
| Microbacterium | 0.61 | 19 | 31 | Microbacterium trichothecenolyticum | 0 | 0 | 54/4524680 | 0.0012 | 1 |
| Mycobacterium | 0.18 | 13 | 21 | Mycobacterium mucogenicum | 10 | 16 | 704/6475901 | 0.0109 | 1 |
| Mycobacterium | 0.18 | 13 | 21 | Mycobacterium europaeum | 1 | 2 | 75/6152643 | 0.0012 | 1 |
| Mycobacterium | 0.18 | 13 | 21 | Mycobacterium iranicum | 1 | 2 | 119/6138463 | 0.0019 | 1 |
| Mycobacterium | 0.18 | 13 | 21 | Mycobacterium celatum | 0 | 0 | 43/4674213 | 0.0009 | 1 |
| Mycobacterium | 0.18 | 13 | 21 | Mycobacterium paraffinicum | 0 | 0 | 75/6476152 | 0.0012 | 1 |
| Mycobacterium | 0.18 | 13 | 21 | Mycobacterium parascrofulaceum | 0 | 0 | 56/6565401 | 0.0009 | 1 |
| Mycobacterium | 0.18 | 13 | 21 | Mycobacterium smegmatis | 0 | 0 | 61/6988302 | 0.0009 | 1 |
| Polynucleobacter | 0.89 | 11 | 18 | Polynucleobacter necessarius | 11 | 18 | 2010/2159490 | 0.0931 | 1 |
| Flavobacterium | 0.7 | 10 | 16 | Flavobacterium succinicans | 3 | 5 | 639/3665414 | 0.0174 | 1 |
| Flavobacterium | 0.7 | 10 | 16 | Flavobacterium aquatile | 1 | 2 | 457/3488993 | 0.0131 | 1 |
| Flavobacterium | 0.7 | 10 | 16 | Flavobacterium branchiophilum | 1 | 2 | 226/3559884 | 0.0063 | 1 |
| Flavobacterium | 0.7 | 10 | 16 | Flavobacterium degerlachei | 1 | 2 | 427/3856829 | 0.0111 | 1 |
| Flavobacterium | 0.7 | 10 | 16 | Flavobacterium psychrophilum | 1 | 2 | 208/2900735 | 0.0072 | 1 |
| Flavobacterium | 0.7 | 10 | 16 | Flavobacterium columnare | 0 | 0 | 62/3329120 | 0.0019 | 1 |
| Flavobacterium | 0.7 | 10 | 16 | Flavobacterium indicum | 0 | 0 | 150/2993089 | 0.005 | 1 |
| Flavobacterium | 0.7 | 10 | 16 | Flavobacterium johnsoniae | 0 | 0 | 470/6096872 | 0.0077 | 1 |
| Gordonia | 0.15 | 10 | 16 | Gordonia polyisoprenivorans | 6 | 10 | 394/5669805 | 0.0069 | 1 |
| Gordonia | 0.15 | 10 | 16 | Gordonia alkanivorans | 1 | 2 | 41/5181810 | 0.0008 | 1 |
| Gordonia | 0.15 | 10 | 16 | Gordonia bronchialis | 1 | 2 | 107/5208602 | 0.0021 | 1 |
| Gordonia | 0.15 | 10 | 16 | Gordonia paraffinivorans | 1 | 2 | 75/4633432 | 0.0016 | 1 |
| Gordonia | 0.15 | 10 | 16 | Gordonia KTR9 | 0 | 0 | 135/5441391 | 0.0025 | 1 |
| Bradyrhizobium | 0.79 | 9 | 15 | Bradyrhizobium S23321 | 3 | 5 | 1620/7231841 | 0.0224 | 1 |
| Bradyrhizobium | 0.79 | 9 | 15 | Bradyrhizobium japonicum | 2 | 3 | 2144/9207384 | 0.0233 | 1 |
| Bradyrhizobium | 0.79 | 9 | 15 | Bradyrhizobium BTAi1 | 1 | 2 | 1018/8264687 | 0.0123 | 1 |
| Bradyrhizobium | 0.79 | 9 | 15 | Bradyrhizobium oligotrophicum | 0 | 0 | 1242/8264165 | 0.015 | 1 |
| Bradyrhizobium | 0.79 | 9 | 15 | Bradyrhizobium ORS | 0 | 0 | 1009/7456587 | 0.0135 | 1 |
| Deinococcus | 0.38 | 8 | 13 | Deinococcus geothermalis | 4 | 7 | 400/2467205 | 0.0162 | 1 |
| Deinococcus | 0.38 | 8 | 13 | Deinococcus radiodurans | 2 | 3 | 159/3060986 | 0.0052 | 1 |
| Deinococcus | 0.38 | 8 | 13 | Deinococcus ficus | 1 | 2 | 219/4144928 | 0.0053 | 1 |
| Deinococcus | 0.38 | 8 | 13 | Deinococcus apachensis | 0 | 0 | 75/4453152 | 0.0017 | 1 |
| Deinococcus | 0.38 | 8 | 13 | Deinococcus gobiensis | 0 | 0 | 253/3137147 | 0.0081 | 1 |
| Deinococcus | 0.38 | 8 | 13 | Deinococcus proteolyticus | 0 | 0 | 68/2147060 | 0.0032 | 1 |
| Janibacter | 0.19 | 8 | 13 | Janibacter melonis | 8 | 13 | 716/3196938 | 0.0224 | 1 |
| Sphingobium | 0.56 | 8 | 13 | Sphingobium xenophagum | 6 | 10 | 1034/4487790 | 0.023 | 1 |
| Sphingobium | 0.56 | 8 | 13 | Sphingobium SYK | 1 | 2 | 179/4199332 | 0.0043 | 1 |
| Sphingobium | 0.56 | 8 | 13 | Sphingobium chlorophenolicum | 0 | 0 | 291/4449488 | 0.0065 | 1 |
| Sphingobium | 0.56 | 8 | 13 | Sphingobium japonicum | 0 | 0 | 507/4196714 | 0.0121 | 1 |
| Sphingobium | 0.56 | 8 | 13 | Sphingobium yanoikuyae | 0 | 0 | 820/5532659 | 0.0148 | 1 |
| Mesorhizobium | 0.26 | 7 | 11 | Mesorhizobium australicum | 1 | 2 | 335/6200534 | 0.0054 | 1 |
| Mesorhizobium | 0.26 | 7 | 11 | Mesorhizobium ciceri | 1 | 2 | 332/6264489 | 0.0053 | 1 |
| Mesorhizobium | 0.26 | 7 | 11 | Mesorhizobium loti | 1 | 2 | 332/7036071 | 0.0047 | 1 |
| Mesorhizobium | 0.26 | 7 | 11 | Mesorhizobium opportunistum | 0 | 0 | 594/6884444 | 0.0086 | 1 |
| Dyella | 0.32 | 6 | 10 | Dyella japonica | 6 | 10 | 1622/4831185 | 0.0336 | 1 |
| Bacteroides | 0.11 | 5 | 8 | Bacteroides ovatus | 1 | 2 | 75/6475296 | 0.0012 | 1 |
| Bacteroides | 0.11 | 5 | 8 | Bacteroides vulgatus | 1 | 2 | 120/5163189 | 0.0023 | 1 |
| Bacteroides | 0.11 | 5 | 8 | Bacteroides dorei | 0 | 0 | 53/5444912 | 0.001 | 1 |
| Bacteroides | 0.11 | 5 | 8 | Bacteroides fluxus | 0 | 0 | 50/4331676 | 0.0012 | 1 |
| Bacteroides | 0.11 | 5 | 8 | Bacteroides neonati | 0 | 0 | 43/5025504 | 0.0009 | 1 |
| Bacteroides | 0.11 | 5 | 8 | Bacteroides pyogenes | 0 | 0 | 42/3569364 | 0.0012 | 1 |
| Bacteroides | 0.11 | 5 | 8 | Bacteroides uniformis | 0 | 0 | 71/4959011 | 0.0014 | 1 |
| Bacteroides | 0.11 | 5 | 8 | Bacteroides xylanisolvens | 0 | 0 | 59/5976145 | 0.001 | 1 |
| Micrococcus | 0.26 | 5 | 8 | Micrococcus luteus | 3 | 5 | 463/2501097 | 0.0185 | 1 |
| Micrococcus | 0.26 | 5 | 8 | Micrococcus lylae | 1 | 2 | 191/2686748 | 0.0071 | 1 |
| Micrococcus | 0.26 | 5 | 8 | Micrococcus terreus | 1 | 2 | 44/3087820 | 0.0014 | 1 |
| Ochrobactrum | 0.1 | 5 | 8 | Ochrobactrum anthropi | 5 | 8 | 491/4783208 | 0.0103 | 1 |
| Ochrobactrum | 0.1 | 5 | 8 | Ochrobactrum intermedium | 0 | 0 | 53/4665240 | 0.0011 | 1 |
| Andreesenia | 0.19 | 4 | 7 | Andreesenia angusta | 4 | 7 | 473/2343873 | 0.0202 | 1 |
| Aquitalea | 0.18 | 4 | 7 | Aquitalea pelogenes | 4 | 7 | 837/4392639 | 0.0191 | 1 |
| Stenotrophomonas | 0.11 | 4 | 7 | Stenotrophomonas maltophilia | 4 | 7 | 555/4509724 | 0.0123 | 1 |
| Yersinia | 0.08 | 4 | 7 | Yersinia intermedia | 2 | 3 | 196/4859749 | 0.004 | 1 |
| Yersinia | 0.08 | 4 | 7 | Yersinia enterocolitica | 1 | 2 | 75/4807490 | 0.0016 | 1 |
| Yersinia | 0.08 | 4 | 7 | Yersinia aleksiciae | 0 | 0 | 33/4000307 | 0.0008 | 1 |
| Yersinia | 0.08 | 4 | 7 | Yersinia massiliensis | 0 | 0 | 42/5050486 | 0.0008 | 1 |
| Alistipes | 0.07 | 3 | 5 | Alistipes finegoldii | 1 | 2 | 40/3734239 | 0.0011 | 1 |
| Alistipes | 0.07 | 3 | 5 | Alistipes inops | 1 | 2 | 42/2305471 | 0.0018 | 1 |
| Alistipes | 0.07 | 3 | 5 | Alistipes putredinis | 1 | 2 | 64/2550788 | 0.0025 | 1 |
| Aliterella | 0.07 | 3 | 5 | Aliterella atlantica | 3 | 5 | 421/5266293 | 0.008 | 1 |
| Aquabacterium | 0.11 | 3 | 5 | Aquabacterium parvum | 3 | 5 | 504/4599179 | 0.011 | 1 |
| Blautia | 0.06 | 3 | 5 | Blautia wexlerae | 2 | 3 | 210/4510558 | 0.0047 | 1 |
| Blautia | 0.06 | 3 | 5 | Ruminococcus torques | 0 | 0 | 75/3341681 | 0.0022 | 1 |
| Caulobacter | 0.19 | 3 | 5 | Caulobacter crescentus | 1 | 2 | 358/4042929 | 0.0089 | 1 |
| Caulobacter | 0.19 | 3 | 5 | Caulobacter K31 | 1 | 2 | 208/5477872 | 0.0038 | 1 |
| Caulobacter | 0.19 | 3 | 5 | Caulobacter segnis | 1 | 2 | 255/4655622 | 0.0055 | 1 |
| Cutibacterium | 0.19 | 3 | 5 | Cutibacterium granulosum | 2 | 3 | 150/2141325 | 0.007 | 1 |
| Cutibacterium | 0.19 | 3 | 5 | Propionibacterium namnetense | 1 | 2 | 269/2369944 | 0.0114 | 1 |
| Cutibacterium | 0.19 | 3 | 5 | Cutibacterium avidum | 0 | 0 | 68/2729848 | 0.0025 | 1 |
| Enhydrobacter | 0.13 | 3 | 5 | Enhydrobacter aerosaccus | 3 | 5 | 860/6770053 | 0.0127 | 1 |
| Enterobacter | 0.06 | 3 | 5 | Enterobacter kobei | 2 | 3 | 190/4880257 | 0.0039 | 1 |
| Enterobacter | 0.06 | 3 | 5 | Enterobacter hormaechei | 0 | 0 | 70/4897485 | 0.0014 | 1 |
| Enterobacter | 0.06 | 3 | 5 | Enterobacter massiliensis | 0 | 0 | 61/4924597 | 0.0012 | 1 |
| Eubacterium | 0.06 | 3 | 5 | Eubacterium rectale | 2 | 3 | 88/3698419 | 0.0024 | 1 |
| Eubacterium | 0.06 | 3 | 5 | Eubacterium eligens | 1 | 2 | 64/2144190 | 0.003 | 1 |
| Halomonas | 0.07 | 3 | 5 | Halomonas stevensii | 3 | 5 | 213/3694265 | 0.0058 | 1 |
| Halomonas | 0.07 | 3 | 5 | Halomonas elongata | 0 | 0 | 63/4061296 | 0.0016 | 1 |
| Hydrotalea | 0.18 | 3 | 5 | Hydrotalea flava | 3 | 5 | 641/3414946 | 0.0188 | 1 |
| Limnohabitans | 0.18 | 3 | 5 | Limnohabitans planktonicus | 3 | 5 | 930/4742314 | 0.0196 | 1 |
| Methylibium | 0.14 | 3 | 5 | Methylibium petroleiphilum | 3 | 5 | 587/4044195 | 0.0145 | 1 |
| Methyloversatilis | 0.1 | 3 | 5 | Methyloversatilis discipulorum | 3 | 5 | 482/4304808 | 0.0112 | 1 |
| Psychrobacter | 0.13 | 3 | 5 | Psychrobacter PRwf | 3 | 5 | 209/2978976 | 0.007 | 1 |
| Psychrobacter | 0.13 | 3 | 5 | Psychrobacter arcticus | 0 | 0 | 119/2650701 | 0.0045 | 1 |
| Psychrobacter | 0.13 | 3 | 5 | Psychrobacter G | 0 | 0 | 75/3079438 | 0.0024 | 1 |
| Rhodococcus | 0.05 | 3 | 5 | Rhodococcus fascians | 2 | 3 | 150/5139988 | 0.0029 | 1 |
| Rhodococcus | 0.05 | 3 | 5 | Rhodococcus erythropolis | 1 | 2 | 56/6516310 | 0.0009 | 1 |
| Rhodococcus | 0.05 | 3 | 5 | Rhodococcus globerulus | 0 | 0 | 69/6735527 | 0.001 | 1 |
| Rhodococcus | 0.05 | 3 | 5 | Rhodococcus opacus | 0 | 0 | 42/7913450 | 0.0005 | 1 |
| Roseomonas | 0.06 | 3 | 5 | Roseomonas mucosa | 2 | 3 | 194/4865340 | 0.004 | 1 |
| Roseomonas | 0.06 | 3 | 5 | Roseomonas cervicalis | 0 | 0 | 111/5105947 | 0.0022 | 1 |
| Acetanaerobacterium | 0.04 | 2 | 3 | Acetanaerobacterium elongatum | 2 | 3 | 150/2916935 | 0.0051 | 1 |
| Aeromicrobium | 0.07 | 2 | 3 | Aeromicrobium choanae | 2 | 3 | 300/3422026 | 0.0088 | 1 |
| Aeromonas | 0.04 | 2 | 3 | Aeromonas media | 1 | 2 | 39/4777154 | 0.0008 | 1 |
| Aeromonas | 0.04 | 2 | 3 | Aeromonas fluvialis | 0 | 0 | 71/3904978 | 0.0018 | 1 |
| Aeromonas | 0.04 | 2 | 3 | Aeromonas hydrophila | 0 | 0 | 44/5127362 | 0.0009 | 1 |
| Agrobacterium | 0.14 | 2 | 3 | Agrobacterium fabrum | 0 | 0 | 358/4917167 | 0.0073 | 1 |
| Agrobacterium | 0.14 | 2 | 3 | Agrobacterium H13 | 0 | 0 | 276/4972229 | 0.0056 | 1 |
| Agrobacterium | 0.14 | 2 | 3 | Agrobacterium tumefaciens | 0 | 0 | 127/5481605 | 0.0023 | 1 |
| Algoriella | 0.04 | 2 | 3 | Algoriella xinjiangensis | 2 | 3 | 136/3266341 | 0.0042 | 1 |
| Alishewanella | 0.04 | 2 | 3 | Alishewanella agri | 2 | 3 | 150/3491999 | 0.0043 | 1 |
| Arcobacter | 0.06 | 2 | 3 | Arcobacter cryaerophilus | 2 | 3 | 117/2210034 | 0.0053 | 1 |
| Bordetella | 0.13 | 2 | 3 | Bordetella hinzii | 2 | 3 | 178/4912977 | 0.0036 | 1 |
| Bordetella | 0.13 | 2 | 3 | Bordetella avium | 0 | 0 | 55/3732255 | 0.0015 | 1 |
| Bordetella | 0.13 | 2 | 3 | Bordetella bronchialis | 0 | 0 | 190/5966919 | 0.0032 | 1 |
| Bordetella | 0.13 | 2 | 3 | Bordetella bronchiseptica | 0 | 0 | 76/5339179 | 0.0014 | 1 |
| Bordetella | 0.13 | 2 | 3 | Bordetella holmesii | 0 | 0 | 52/3701221 | 0.0014 | 1 |
| Bordetella | 0.13 | 2 | 3 | Bordetella petrii | 0 | 0 | 42/5287950 | 0.0008 | 1 |
| Bosea | 0.1 | 2 | 3 | Bosea lupini | 2 | 3 | 667/6081108 | 0.011 | 1 |
| Capnocytophaga | 0.06 | 2 | 3 | Capnocytophaga gingivalis | 1 | 2 | 75/2667498 | 0.0028 | 1 |
| Capnocytophaga | 0.06 | 2 | 3 | Capnocytophaga sputigena | 1 | 2 | 132/2998485 | 0.0044 | 1 |
| Carnobacterium | 0.04 | 2 | 3 | Carnobacterium viridans | 1 | 2 | 150/2762407 | 0.0054 | 1 |
| Comamonas | 0.28 | 2 | 3 | Comamonas kerstersii | 1 | 2 | 262/3734555 | 0.007 | 1 |
| Comamonas | 0.28 | 2 | 3 | Comamonas testosteroni | 1 | 2 | 184/5373644 | 0.0034 | 1 |
| Comamonas | 0.28 | 2 | 3 | Comamonas aquatica | 0 | 0 | 400/3764434 | 0.0106 | 1 |
| Comamonas | 0.28 | 2 | 3 | Comamonas terrae | 0 | 0 | 316/4717103 | 0.0067 | 1 |
| Giesbergeria | 0.11 | 2 | 3 | Giesbergeria anulus | 2 | 3 | 320/3420882 | 0.0094 | 1 |
| Janthinobacterium | 0.06 | 2 | 3 | Janthinobacterium Marseille | 2 | 3 | 231/4110251 | 0.0056 | 1 |
| Kytococcus | 0.04 | 2 | 3 | Kytococcus sedentarius | 2 | 3 | 121/2785024 | 0.0043 | 1 |
| Lactobacillus | 0.1 | 2 | 3 | Lactobacillus crispatus | 1 | 2 | 128/2043161 | 0.0063 | 1 |
| Lactobacillus | 0.1 | 2 | 3 | Lactobacillus rhamnosus | 0 | 0 | 54/3005051 | 0.0018 | 1 |
| Lactobacillus | 0.1 | 2 | 3 | Lactobacillus zymae | 0 | 0 | 46/2734129 | 0.0017 | 1 |
| Leptospira | 0.03 | 2 | 3 | Leptospira alstonii | 1 | 2 | 53/4591898 | 0.0012 | 1 |
| Leptospira | 0.03 | 2 | 3 | Leptospira noguchii | 1 | 2 | 75/4763760 | 0.0016 | 1 |
| Massilia | 0.12 | 2 | 3 | Massilia alkalitolerans | 0 | 0 | 258/6112424 | 0.0042 | 1 |
| Massilia | 0.12 | 2 | 3 | Massilia putida | 0 | 0 | 328/6987670 | 0.0047 | 1 |
| Massilia | 0.12 | 2 | 3 | Massilia timonae | 0 | 0 | 84/6136630 | 0.0014 | 1 |
| Melaminivora | 0.08 | 2 | 3 | Melaminivora alkalimesophila | 2 | 3 | 250/3016125 | 0.0083 | 1 |
| Methylophilus | 0.15 | 2 | 3 | Methylophilus methylotrophus | 2 | 3 | 502/2860349 | 0.0176 | 1 |
| Novosphingobium | 0.2 | 2 | 3 | Novosphingobium aromaticivorans | 1 | 2 | 576/3561584 | 0.0162 | 1 |
| Novosphingobium | 0.2 | 2 | 3 | Novosphingobium PP1Y | 1 | 2 | 136/3911486 | 0.0035 | 1 |
| Obesumbacterium | 0.09 | 2 | 3 | Obesumbacterium proteus | 2 | 3 | 460/5011796 | 0.0092 | 1 |
| Pandoraea | 0.09 | 2 | 3 | Pandoraea norimbergensis | 1 | 2 | 75/6167370 | 0.0012 | 1 |
| Pandoraea | 0.09 | 2 | 3 | Pandoraea apista | 0 | 0 | 48/5609637 | 0.0009 | 1 |
| Pandoraea | 0.09 | 2 | 3 | Pandoraea pnomenusa | 0 | 0 | 146/5584076 | 0.0026 | 1 |
| Pandoraea | 0.09 | 2 | 3 | Pandoraea pulmonicola | 0 | 0 | 63/5867621 | 0.0011 | 1 |
| Pandoraea | 0.09 | 2 | 3 | Pandoraea sputorum | 0 | 0 | 125/5742997 | 0.0022 | 1 |
| Prevotella | 0.06 | 2 | 3 | Prevotella bivia | 1 | 2 | 68/2580302 | 0.0026 | 1 |
| Prevotella | 0.06 | 2 | 3 | Prevotella loescheii | 1 | 2 | 60/3508889 | 0.0017 | 1 |
| Prevotella | 0.06 | 2 | 3 | Prevotella nigrescens | 0 | 0 | 54/2987072 | 0.0018 | 1 |
| Shigella | 0.07 | 2 | 3 | Shigella dysenteriae | 1 | 2 | 140/4369232 | 0.0032 | 1 |
| Shigella | 0.07 | 2 | 3 | Shigella flexneri | 0 | 0 | 111/4698633 | 0.0024 | 1 |
| Shigella | 0.07 | 2 | 3 | Shigella sonnei | 0 | 0 | 69/4878853 | 0.0014 | 1 |
| Streptococcus | 0.15 | 2 | 3 | Streptococcus gordonii | 1 | 2 | 75/2196662 | 0.0034 | 1 |
| Streptococcus | 0.15 | 2 | 3 | Streptococcus oralis | 0 | 0 | 55/1958690 | 0.0028 | 1 |
| Streptococcus | 0.15 | 2 | 3 | Streptococcus pneumoniae | 0 | 0 | 132/2150813 | 0.0061 | 1 |
| Streptococcus | 0.15 | 2 | 3 | Streptococcus salivarius | 0 | 0 | 75/2191044 | 0.0034 | 1 |
| Xanthomonas | 0.21 | 2 | 3 | Xanthomonas campestris | 1 | 2 | 355/4908383 | 0.0072 | 1 |
| Xanthomonas | 0.21 | 2 | 3 | Xanthomonas translucens | 1 | 2 | 405/4715357 | 0.0086 | 1 |
| Xanthomonas | 0.21 | 2 | 3 | Xanthomonas albilineans | 0 | 0 | 49/3768695 | 0.0013 | 1 |
| Xanthomonas | 0.21 | 2 | 3 | Xanthomonas fuscans | 0 | 0 | 75/4981995 | 0.0015 | 1 |
| Xanthomonas | 0.21 | 2 | 3 | Xanthomonas vasicola | 0 | 0 | 46/5334668 | 0.0009 | 1 |
| Achromobacter | 0.05 | 1 | 2 | Achromobacter xylosoxidans | 1 | 2 | 176/6813182 | 0.0026 | 1 |
| Achromobacter | 0.05 | 1 | 2 | Achromobacter ruhlandii | 0 | 0 | 106/6687427 | 0.0016 | 1 |
| Actinomyces | 0.06 | 1 | 2 | Actinomyces massiliensis | 1 | 2 | 75/3371154 | 0.0022 | 1 |
| Actinomyces | 0.06 | 1 | 2 | Actinomyces georgiae | 0 | 0 | 75/2498243 | 0.003 | 1 |
| Actinomyces | 0.06 | 1 | 2 | Actinomyces polynesiensis | 0 | 0 | 75/2881529 | 0.0026 | 1 |
| Afipia | 0.24 | 1 | 2 | Afipia birgiae | 1 | 2 | 996/5334186 | 0.0187 | 1 |
| Afipia | 0.24 | 1 | 2 | Afipia felis | 0 | 0 | 272/4203390 | 0.0065 | 1 |
| Agrococcus | 0.02 | 1 | 2 | Agrococcus baldri | 1 | 2 | 75/2904536 | 0.0026 | 1 |
| Akkermansia | 0.02 | 1 | 2 | Akkermansia muciniphila | 1 | 2 | 61/2664102 | 0.0023 | 1 |
| Alcaligenes | 0.03 | 1 | 2 | Alcaligenes faecalis | 1 | 2 | 129/4233756 | 0.003 | 1 |
| Alicycliphilus | 0.09 | 1 | 2 | Alicycliphilus denitrificans | 1 | 2 | 502/4995263 | 0.01 | 1 |
| Anaerosalibacter | 0.02 | 1 | 2 | Anaerosalibacter massiliensis | 1 | 2 | 58/3197412 | 0.0018 | 1 |
| Auricoccucs | 0.07 | 1 | 2 | Auricoccus indicus | 1 | 2 | 120/1745789 | 0.0069 | 1 |
| Azospirillum | 0.09 | 1 | 2 | Azospirillum halopraeferens | 1 | 2 | 123/6512867 | 0.0019 | 1 |
| Azospirillum | 0.09 | 1 | 2 | Azospirillum B510 | 0 | 0 | 41/3311395 | 0.0012 | 1 |
| Azospirillum | 0.09 | 1 | 2 | Azospirillum lipoferum | 0 | 0 | 88/2988332 | 0.0029 | 1 |
| Bifidobacterium | 0.06 | 1 | 2 | Bifidobacterium animalis | 1 | 2 | 54/1958651 | 0.0028 | 1 |
| Bifidobacterium | 0.06 | 1 | 2 | Bifidobacterium pseudolongum | 0 | 0 | 46/2032698 | 0.0023 | 1 |
| Brevibacterium | 0.09 | 1 | 2 | Brevibacterium mcbrellneri | 1 | 2 | 122/2562754 | 0.0048 | 1 |
| Brevibacterium | 0.09 | 1 | 2 | Brevibacterium epidermidis | 0 | 0 | 59/3703501 | 0.0016 | 1 |
| Brevibacterium | 0.09 | 1 | 2 | Brevibacterium luteolum | 0 | 0 | 46/3100698 | 0.0015 | 1 |
| Caballeronia | 0.09 | 1 | 2 | Caballeronia sordidicola | 1 | 2 | 409/10264041 | 0.004 | 1 |
| Caballeronia | 0.09 | 1 | 2 | Caballeronia concitans | 0 | 0 | 320/6166631 | 0.0052 | 1 |
| Caldimonas | 0.02 | 1 | 2 | Caldimonas manganoxidans | 1 | 2 | 41/3532996 | 0.0012 | 1 |
| Cellulomonas | 0.03 | 1 | 2 | Cellulomonas flavigena | 1 | 2 | 39/4123179 | 0.0009 | 1 |
| Cellulomonas | 0.03 | 1 | 2 | Cellulomonas gilvus | 0 | 0 | 42/3526441 | 0.0012 | 1 |
| Chelatococcus | 0.07 | 1 | 2 | Chelatococcus sambhunathii | 1 | 2 | 258/4374160 | 0.0059 | 1 |
| Chryseobacterium | 0.02 | 1 | 2 | Chryseobacterium koreense | 1 | 2 | 58/3154833 | 0.0018 | 1 |
| Cloacibacterium | 0.09 | 1 | 2 | Cloacibacterium normanense | 1 | 2 | 269/2736686 | 0.0098 | 1 |
| Collimonas | 0.12 | 1 | 2 | Collimonas fungivorans | 1 | 2 | 437/5186898 | 0.0084 | 1 |
| Collimonas | 0.12 | 1 | 2 | Collimonas pratensis | 0 | 0 | 211/5730025 | 0.0037 | 1 |
| Cytophaga | 0.03 | 1 | 2 | Cytophaga hutchinsonii | 1 | 2 | 133/4433218 | 0.003 | 1 |
| Dichelobacter | 0.04 | 1 | 2 | Dichelobacter nodosus | 1 | 2 | 50/1389350 | 0.0036 | 1 |
| Dietzia | 0.05 | 1 | 2 | Dietzia natronolimnaea | 1 | 2 | 44/3792731 | 0.0012 | 1 |
| Dietzia | 0.05 | 1 | 2 | Dietzia cinnamea | 0 | 0 | 137/4144030 | 0.0033 | 1 |
| Dolosigranulum | 0.03 | 1 | 2 | Dolosigranulum pigrum | 1 | 2 | 47/1862145 | 0.0025 | 1 |
| Duganella | 0.04 | 1 | 2 | Duganella sacchari | 1 | 2 | 220/6676036 | 0.0033 | 1 |
| Ensifer | 0.03 | 1 | 2 | Ensifer adhaerens | 1 | 2 | 131/4071185 | 0.0032 | 1 |
| Enterococcus | 0.01 | 1 | 2 | Enterococcus malodoratus | 1 | 2 | 52/4654327 | 0.0011 | 1 |
| Escherichia | 0.01 | 1 | 2 | Escherichia fergusonii | 1 | 2 | 714588711 | 0.0015 | 1 |
| Frankia | 0.01 | 1 | 2 | Frankia symbiont | 1 | 2 | 35/5323186 | 0.0007 | 1 |
| Friedmanniella | 0.03 | 1 | 2 | Friedmanniella flava | 1 | 2 | 1414788805 | 0.0029 | 1 |
| Fusobacterium | 0.02 | 1 | 2 | Fusobacterium periodonticum | 1 | 2 | 40/2615523 | 0.0015 | 1 |
| Gemella | 0.09 | 1 | 2 | Gemella haemolysans | 1 | 2 | 178/2053699 | 0.0087 | 1 |
| Granulibacter | 0.02 | 1 | 2 | Granulibacter bethesdensis | 1 | 2 | 40/2742670 | 0.0015 | 1 |
| Hafnia | 0.04 | 1 | 2 | Hafnia paralvei | 1 | 2 | 75/4998684 | 0.0015 | 1 |
| Hafnia | 0.04 | 1 | 2 | Hafnia alvei | 0 | 0 | 136/4712721 | 0.0029 | 1 |
| Herbaspirillum | 0.03 | 1 | 2 | Herbaspirillum seropedicae | 1 | 2 | 202/5513887 | 0.0037 | 1 |
| Holdemanella | 0.02 | 1 | 2 | Holdemanella biformis | 1 | 2 | 75/2518193 | 0.003 | 1 |
| Hydrogenophaga | 0.25 | 1 | 2 | Hydrogenophaga taeniospiralis | 1 | 2 | 1305/5275851 | 0.0247 | 1 |
| Hyphomicrobium | 0.08 | 1 | 2 | Hyphomicrobium MC1 | 1 | 2 | 323/4757528 | 0.0068 | 1 |
| Hyphomicrobium | 0.08 | 1 | 2 | Hyphomicrobium denitrificans | 0 | 0 | 53/3808687 | 0.0014 | 1 |
| Kingella | 0.05 | 1 | 2 | Kingella denitrificans | 1 | 2 | 49/2220565 | 0.0022 | 1 |
| Kingella | 0.05 | 1 | 2 | Kingella oralis | 0 | 0 | 36/2406715 | 0.0015 | 1 |
| Kocuria | 0.02 | 1 | 2 | Kocuria palustris | 0 | 0 | 40/2854447 | 0.0014 | 1 |
| Lactococcus | 0.02 | 1 | 2 | Lactococcus lactis | 1 | 2 | 75/2598144 | 0.0029 | 1 |
| Lautropia | 0.04 | 1 | 2 | Lautropia mirabilis | 1 | 2 | 110/3151995 | 0.0035 | 1 |
| Leadbetterella | 0.02 | 1 | 2 | Leadbetterella byssophila | 1 | 2 | 64/4059653 | 0.0016 | 1 |
| Mesonia | 0.02 | 1 | 2 | Mesonia mobilis | 1 | 2 | 38/3206138 | 0.0012 | 1 |
| Micromonospora | 0.02 | 1 | 2 | Micromonospora aurantiaca | 0 | 0 | 75/7025559 | 0.0011 | 1 |
| Micromonospora | 0.02 | 1 | 2 | Micromonospora L5 | 0 | 0 | 75/6962533 | 0.0011 | 1 |
| Natrinema | 0.02 | 1 | 2 | Natrinema J7 | 1 | 2 | 61/3697626 | 0.0016 | 1 |
| Neisseria | 0.23 | 1 | 2 | Neisseria canis | 0 | 0 | 70/2530349 | 0.0028 | 1 |
| Neisseria | 0.23 | 1 | 2 | Neisseria perflava | 0 | 0 | 721/3786149 | 0.019 | 1 |
| Neisseria | 0.23 | 1 | 2 | Neisseria sicca | 0 | 0 | 66/2831372 | 0.0023 | 1 |
| Parvimonas | 0.04 | 1 | 2 | Parvimonas micra | 1 | 2 | 75/1627009 | 0.0046 | 1 |
| Pseudoxanthomonas | 0.22 | 1 | 2 | Pseudoxanthomonas Mexicana | 1 | 2 | 436/3943279 | 0.0111 | 1 |
| Pseudoxanthomonas | 0.22 | 1 | 2 | Pseudoxanthomonas spadix | 0 | 0 | 116/3452554 | 0.0034 | 1 |
| Pseudoxanthomonas | 0.22 | 1 | 2 | Pseudoxanthomonas suwonensis | 0 | 0 | 245/3419049 | 0.0072 | 1 |
| Pusillimonas | 0.02 | 1 | 2 | Pusillimonas T7 | 1 | 2 | 75/3883605 | 0.0019 | 1 |
| Rhizobium | 0.13 | 1 | 2 | Rhizobium etli | 0 | 0 | 169/4598466 | 0.0037 | 1 |
| Rhizobium | 0.13 | 1 | 2 | Rhizobium IRBG74 | 0 | 0 | 183/2844565 | 0.0064 | 1 |
| Rhizobium | 0.13 | 1 | 2 | Rhizobium leguminosarum | 0 | 0 | 136/5119898 | 0.0027 | 1 |
| Rhodopseudomonas | 0.13 | 1 | 2 | Rhodopseudomonas palustris | 1 | 2 | 816/5744041 | 0.0142 | 1 |
| Rothia | 0.03 | 1 | 2 | Rothia mucilaginosa | 1 | 2 | 71/2264603 | 0.0031 | 1 |
| Shewanella | 0.03 | 1 | 2 | Shewanella ANA | 0 | 0 | 38/4972204 | 0.0008 | 1 |
| Shewanella | 0.03 | 1 | 2 | Shewanella W3 | 0 | 0 | 46/4708380 | 0.001 | 1 |
| Sphingopyxis | 0.13 | 1 | 2 | Sphingopyxis alaskensis | 1 | 2 | 389/3345170 | 0.0116 | 1 |
| Tannerella | 0.02 | 1 | 2 | Tannerella forsythia | 1 | 2 | 75/3405521 | 0.0022 | 1 |
| Verminephrobacter | 0.07 | 1 | 2 | Verminephrobacter eiseniae | 1 | 2 | 376/5566749 | 0.0068 | 1 |
| Xanthobacter | 0.02 | 1 | 2 | Xanthobacter autotrophicus | 1 | 2 | 115/5308934 | 0.0022 | 1 |
| Acidisphaera | 0.03 | 0 | 0 | Acidisphaera rubrifaciens | 0 | 0 | 122/3874653 | 0.0031 | 1 |
| Acidithrix | 0.02 | 0 | 0 | Acidithrix ferrooxidans | 0 | 0 | 30/4021687 | 0.0007 | 1 |
| Acidocella | 0.02 | 0 | 0 | Acidocella aminolytica | 0 | 0 | 71/3989672 | 0.0018 | 1 |
| Actinomadura | 0.01 | 0 | 0 | Actinomadura mexicana | 0 | 0 | 69/8786881 | 0.0008 | 1 |
| Actinopolyspora | 0.01 | 0 | 0 | Actinopolyspora halophila | 0 | 0 | 58/5353301 | 0.0011 | 1 |
| Actinotalea | 0.02 | 0 | 0 | Actinotalea ferrariae | 0 | 0 | 57/3993327 | 0.0014 | 1 |
| Acuticoccus | 0.01 | 0 | 0 | Acuticoccus yangtzensis | 0 | 0 | 39/5099489 | 0.0008 | 1 |
| Advenella | 0.04 | 0 | 0 | Advenella kashmirensis | 0 | 0 | 61/4365995 | 0.0014 | 1 |
| Advenella | 0.04 | 0 | 0 | Advenella mimigardefordensis | 0 | 0 | 150/4740516 | 0.0032 | 1 |
| Aequorivita | 0.02 | 0 | 0 | Aequorivita sublithincola | 0 | 0 | 57/3520671 | 0.0016 | 1 |
| Afifella | 0.02 | 0 | 0 | Afifella marina | 0 | 0 | 72/3963651 | 0.0018 | 1 |
| Agreia | 0.02 | 0 | 0 | Agreia bicolorata | 0 | 0 | 57/3920909 | 0.0015 | 1 |
| Agromyces | 0.01 | 0 | 0 | Agromyces aureus | 0 | 0 | 64/4373124 | 0.0015 | 1 |
| Albidovulum | 0.02 | 0 | 0 | Albidovulum xiamenense | 0 | 0 | 56/3127871 | 0.0018 | 1 |
| Albimonas | 0.02 | 0 | 0 | Albimonas donghaensis | 0 | 0 | 128/5014684 | 0.0026 | 1 |
| Algibacter | 0.02 | 0 | 0 | Algibacter alginicilyticus | 0 | 0 | 50/3994770 | 0.0013 | 1 |
| Alkanindiges | 0.04 | 0 | 0 | Alkanindiges illinoisensis | 0 | 0 | 113/3299508 | 0.0034 | 1 |
| Allochromatium | 0.02 | 0 | 0 | Allochromatium vinosum | 0 | 0 | 50/3526903 | 0.0014 | 1 |
| Altererythrobacter | 0.08 | 0 | 0 | Altererythrobacter atlanticus | 0 | 0 | 72/3386291 | 0.0021 | 1 |
| Altererythrobacter | 0.08 | 0 | 0 | Altererythrobacter dongtanensis | 0 | 0 | 156/3009495 | 0.0052 | 1 |
| Alteromonas | 0.01 | 0 | 0 | Alteromonas stellipolaris | 0 | 0 | 61/4686200 | 0.0013 | 1 |
| Amantichitinum | 0.04 | 0 | 0 | Amantichitinum ursilacus | 0 | 0 | 154/4929321 | 0.0031 | 1 |
| Aminobacter | 0.03 | 0 | 0 | Aminobacter aminovorans | 0 | 0 | 178/5623946 | 0.0032 | 1 |
| Amorphus | 0.01 | 0 | 0 | Amorphus coralli | 0 | 0 | 75/4317655 | 0.0017 | 1 |
| Amycolatopsis | 0.01 | 0 | 0 | Amycolatopsis japonica | 0 | 0 | 75/8961318 | 0.0008 | 1 |
| Amycolatopsis | 0.01 | 0 | 0 | Amycolatopsis mediterranei | 0 | 0 | 75/10246920 | 0.0007 | 1 |
| Anaerorhabdus | 0.03 | 0 | 0 | Anaerorhabdus furcosa | 0 | 0 | 50/2407051 | 0.0021 | 1 |
| Anaerostipes | 0.02 | 0 | 0 | Anaerostipes caccae | 0 | 0 | 52/3607186 | 0.0014 | 1 |
| Ancylobacter | 0.03 | 0 | 0 | Ancylobacter rudongensis | 0 | 0 | 132/4505254 | 0.0029 | 1 |
| Aquamicrobium | 0.05 | 0 | 0 | Aquamicrobium defluvii | 0 | 0 | 240/4775173 | 0.005 | 1 |
| Aquiflexum | 0.01 | 0 | 0 | Aquiflexum balticum | 0 | 0 | 52/5987195 | 0.0009 | 1 |
| Aquimonas | 0.01 | 0 | 0 | Aquimonas voraii | 0 | 0 | 75/4428557 | 0.0017 | 1 |
| Aquincola | 0.1 | 0 | 0 | Aquincola tertiaricarbonis | 0 | 0 | 547/6320516 | 0.0087 | 1 |
| Arachidicoccus | 0.01 | 0 | 0 | Arachidicoccus rhizosphaerae | 0 | 0 | 35/4869254 | 0.0007 | 1 |
| Arenimonas | 0.06 | 0 | 0 | Arenimonas malthae | 0 | 0 | 150/3116278 | 0.0048 | 1 |
| Arenitalea | 0.02 | 0 | 0 | Arenitalea lutea | 0 | 0 | 59/3378101 | 0.0017 | 1 |
| Asticcacaulis | 0.02 | 0 | 0 | Asticcacaulis excentricus | 0 | 0 | 70/3904170 | 0.0018 | 1 |
| Aureimonas | 0.02 | 0 | 0 | Aureimonas frigidaquae | 0 | 0 | 60/4098523 | 0.0015 | 1 |
| Azoarcus | 0.02 | 0 | 0 | Azoarcus KH32C | 0 | 0 | 133/5081166 | 0.0026 | 1 |
| Azohydromonas | 0.02 | 0 | 0 | Azohydromonas lata | 0 | 0 | 143/7186661 | 0.002 | 1 |
| Azorhizobium | 0.02 | 0 | 0 | Azorhizobium caulinodans | 0 | 0 | 46/5369772 | 0.0009 | 1 |
| Azorhizobium | 0.02 | 0 | 0 | Azorhizobium doebereinerae | 0 | 0 | 44/5818544 | 0.0008 | 1 |
| Azospira | 0.02 | 0 | 0 | Azospira oryzae | 0 | 0 | 36/3806980 | 0.0009 | 1 |
| Beijerinckia | 0.01 | 0 | 0 | Beijerinckia indica | 0 | 0 | 46/4170153 | 0.0011 | 1 |
| Belliella | 0.01 | 0 | 0 | Belliella baltica | 0 | 0 | 49/4196595 | 0.0012 | 1 |
| Belnapia | 0.03 | 0 | 0 | Belnapia moabensis | 0 | 0 | 183/6729903 | 0.0027 | 1 |
| Blastochloris | 0.07 | 0 | 0 | Blastochloris viridis | 0 | 0 | 264/3726627 | 0.0071 | 1 |
| Blastococcus | 0.05 | 0 | 0 | Blastococcus endophyticus | 0 | 0 | 56/4875561 | 0.0011 | 1 |
| Blastococcus | 0.05 | 0 | 0 | Blastococcus saxobsidens | 0 | 0 | 198/4875340 | 0.0041 | 1 |
| Brachybacterium | 0.08 | 0 | 0 | Brachybacterium alimentarium | 0 | 0 | 178/4263776 | 0.0042 | 1 |
| Brachybacterium | 0.08 | 0 | 0 | Brachybacterium faecium | 0 | 0 | 69/3614992 | 0.0019 | 1 |
| Brachybacterium | 0.08 | 0 | 0 | Brachybacterium muris | 0 | 0 | 54/3257876 | 0.0017 | 1 |
| Brachymonas | 0.05 | 0 | 0 | Brachymonas denitrificans | 0 | 0 | 132/2713070 | 0.0049 | 1 |
| Buttiauxella | 0.01 | 0 | 0 | Buttiauxella ferragutiae | 0 | 0 | 36/5087974 | 0.0007 | 1 |
| Caenispirillum | 0.03 | 0 | 0 | Caenispirillum salinarum | 0 | 0 | 99/4953065 | 0.002 | 1 |
| Candidatus_Solibacter | 0.01 | 0 | 0 | Candidatus Solibacter usitatus | 0 | 0 | 66/9965640 | 0.0007 | 1 |
| Catenibacterium | 0.01 | 0 | 0 | Catenibacterium mitsuokai | 0 | 0 | 68/6922420 | 0.001 | 1 |
| Chishuiella | 0.02 | 0 | 0 | Chishuiella changwenlii | 0 | 0 | 65/3444151 | 0.0019 | 1 |
| Chitinibacter | 0.05 | 0 | 0 | Chitinibacter tainanensis | 0 | 0 | 159/3426149 | 0.0046 | 1 |
| Chitinilyticum | 0.02 | 0 | 0 | Chitinilyticum aquatile | 0 | 0 | 37/3685817 | 0.001 | 1 |
| Chondromyces | 0.01 | 0 | 0 | Chondromyces apiculatus | 0 | 0 | 136/11579531 | 0.0012 | 1 |
| Chromobacterium | 0.02 | 0 | 0 | Chromobacterium haemolyticum | 0 | 0 | 44/5030819 | 0.0009 | 1.91 |
| Chroococcidiopsis | 0.01 | 0 | 0 | Chroococcidiopsis thermalis | 0 | 0 | 33/6315792 | 0.0005 | 1 |
| Citreimonas | 0.01 | 0 | 0 | Citreimonas salinaria | 0 | 0 | 61/4260601 | 0.0014 | 1 |
| Citrobacter | 0.01 | 0 | 0 | Citrobacter sedlakii | 0 | 0 | 63/4631756 | 0.0014 | 1 |
| Citromicrobium | 0.02 | 0 | 0 | Citromicrobium bathyomarinum | 0 | 0 | 48/3274004 | 0.0015 | 1 |
| Clavibacter | 0.02 | 0 | 0 | Clavibacter michiganensis | 0 | 0 | 75/3207520 | 0.0023 | 1 |
| Clostridium | 0.01 | 0 | 0 | Clostridium intestinale | 0 | 0 | 43/4677688 | 0.0009 | 1 |
| Cnuella | 0.01 | 0 | 0 | Cnuella takakiae | 0 | 0 | 39/5696137 | 0.0007 | 1 |
| Cnuibacter | 0.03 | 0 | 0 | Cnuibacter physcomitrellae | 0 | 0 | 103/4061502 | 0.0025 | 1 |
| Collinsella | 0.03 | 0 | 0 | Collinsella aerofaciens | 0 | 0 | 58/2440109 | 0.0024 | 1 |
| Coxiella | 0.03 | 0 | 0 | Coxiella burnetii | 0 | 0 | 62/2093477 | 0.003 | 1 |
| Cribrihabitans | 0.01 | 0 | 0 | Cribrihabitans marinus | 0 | 0 | 75/4176242 | 0.0018 | 1 |
| Croceicoccus | 0.06 | 0 | 0 | Croceicoccus mobilis | 0 | 0 | 262/4210389 | 0.0062 | 1 |
| Cruoricaptor | 0.03 | 0 | 0 | Cruoricaptor ignavus | 0 | 0 | 46/2229854 | 0.0021 | 1 |
| Curvibacter | 0.13 | 0 | 0 | Curvibacter delicatus | 0 | 0 | 444/3756831 | 0.0118 | 1 |
| Dechloromonas | 0.05 | 0 | 0 | Dechloromonas agitata | 0 | 0 | 213/3627226 | 0.0059 | 1 |
| Derxia | 0.02 | 0 | 0 | Derxia gummosa | 0 | 0 | 88/5185308 | 0.0017 | 1 |
| Desulfomonile | 0.01 | 0 | 0 | Desulfomonile tiedjei | 0 | 0 | 62/6500104 | 0.001 | 1 |
| Diaphorobacter | 0.03 | 0 | 0 | Diaphorobacter polyhydroxybutyrativorans | 0 | 0 | 114/4061276 | 0.0028 | 1 |
| Dinoroseobacter | 0.02 | 0 | 0 | Dinoroseobacter shibae | 0 | 0 | 75/3789584 | 0.002 | 1 |
| Dokdonella | 0.01 | 0 | 0 | Dokdonella immobilis | 0 | 0 | 58/4636605 | 0.0013 | 1 |
| Edaphobacter | 0.02 | 0 | 0 | Edaphobacter aggregans | 0 | 0 | 190/8180575 | 0.0023 | 1 |
| Elioraea | 0.01 | 0 | 0 | Elioraea tepidiphila | 0 | 0 | 59/4304787 | 0.0014 | 1 |
| Empedobacter | 0.02 | 0 | 0 | Empedobacter brevis | 0 | 0 | 53/3792748 | 0.0014 | 1 |
| Erwinia | 0.01 | 0 | 0 | Erwinia tracheiphila | 0 | 0 | 75/4717534 | 0.0016 | 1 |
| Erythrobacter | 0.08 | 0 | 0 | Erythrobacter flavus | 0 | 0 | 129/3184307 | 0.0041 | 1 |
| Erythrobacter | 0.08 | 0 | 0 | Erythrobacter litoralis | 0 | 0 | 51/3052398 | 0.0017 | 1 |
| Erythrobacter | 0.08 | 0 | 0 | Erythrobacter vulgaris | 0 | 0 | 71/2858686 | 0.0025 | 1 |
| Ewingella | 0.01 | 0 | 0 | Ewingella americana | 0 | 0 | 46/4868720 | 0.0009 | 1 |
| Ferriphaselus | 0.02 | 0 | 0 | Ferriphaselus amnicola | 0 | 0 | 48/2685224 | 0.0018 | 1 |
| Filimonas | 0.01 | 0 | 0 | Filimonas lacunae | 0 | 0 | 72/7814405 | 0.0009 | 1 |
| Fischerella | 0.01 | 0 | 0 | Fischerella thermalis | 0 | 0 | 75/6729979 | 0.0011 | 1 |
| Flavihumibacter | 0.01 | 0 | 0 | Flavihumibacter petaseus | 0 | 0 | 46/5693738 | 0.0008 | 1 |
| Flaviramulus | 0.02 | 0 | 0 | Flaviramulus ichthyoenteri | 0 | 0 | 36/3953270 | 0.0009 | 1 |
| Flectobacillus | 0.01 | 0 | 0 | Flectobacillus major | 0 | 0 | 58/6221090 | 0.0009 | 1 |
| Fontimonas | 0.02 | 0 | 0 | Fontimonas thermophila | 0 | 0 | 72/2733810 | 0.0026 | 1 |
| Formosa | 0.01 | 0 | 0 | Formosa agariphila | 0 | 0 | 47/4228350 | 0.0011 | 1 |
| Frateuria | 0.02 | 0 | 0 | Frateuria aurantia | 0 | 0 | 64/3603458 | 0.0018 | 1 |
| Frischella | 0.02 | 0 | 0 | Frischella perrara | 0 | 0 | 40/2692351 | 0.0015 | 1 |
| Gallionella | 0.02 | 0 | 0 | Gallionella capsiferriformans | 0 | 0 | 75/3162471 | 0.0024 | 1 |
| Geminicoccus | 0.01 | 0 | 0 | Geminicoccus roseus | 0 | 0 | 34/5698410 | 0.0006 | 1 |
| Gemmata | 0.01 | 0 | 0 | Gemmata obscuriglobus | 0 | 0 | 53/9171051 | 0.0006 | 1 |
| Gemmobacter | 0.03 | 0 | 0 | Gemmobacter aquatilis | 0 | 0 | 77/3964368 | 0.0019 | 1 |
| Geodermatophilus | 0.02 | 0 | 0 | Geodermatophilus obscurus | 0 | 0 | 122/5322497 | 0.0023 | 1 |
| Geothrix | 0.02 | 0 | 0 | Geothrix fermentans | 0 | 0 | 39/3290163 | 0.0012 | 1 |
| Gillisia | 0.02 | 0 | 0 | Gillisia marina | 0 | 0 | 68/2982554 | 0.0023 | 1 |
| Glaciecola | 0.01 | 0 | 0 | Glaciecola psychrophila | 0 | 0 | 62/5413691 | 0.0011 | 1 |
| Gloeobacter | 0.01 | 0 | 0 | Gloeobacter JS | 0 | 0 | 58/4724791 | 0.0012 | 1 |
| Gluconacetobacter | 0.02 | 0 | 0 | Gluconacetobacter diazotrophicus | 0 | 0 | 41/3944163 | 0.001 | 1 |
| Gulbenkiania | 0.02 | 0 | 0 | Gulbenkiania indica | 0 | 0 | 63/2848192 | 0.0022 | 1 |
| Haematobacter | 0.03 | 0 | 0 | Haematobacter massiliensis | 0 | 0 | 63/4129200 | 0.0015 | 1 |
| Haematobacter | 0.03 | 0 | 0 | Haematobacter missouriensis | 0 | 0 | 50/3960158 | 0.0013 | 1 |
| Halofilum | 0.02 | 0 | 0 | Halofilum ochraceum | 0 | 0 | 68/3644642 | 0.0019 | 1 |
| Halolamina | 0.02 | 0 | 0 | Halolamina pelagica | 0 | 0 | 37/3160993 | 0.0012 | 1 |
| Herbiconiux | 0.03 | 0 | 0 | Herbiconiux solani | 0 | 0 | 86/3849939 | 0.0022 | 1 |
| Herminiimonas | 0.07 | 0 | 0 | Herminiimonas arsenicoxydans | 0 | 0 | 197/3424307 | 0.0058 | 1.15 |
| Hydrobacter | 0.06 | 0 | 0 | Hydrobacter penzbergensis | 0 | 0 | 259/3961899 | 0.0065 | 1 |
| Hylemonella | 0.1 | 0 | 0 | Hylemonella gracilis | 0 | 0 | 325/3821625 | 0.0085 | 1 |
| Hymenobacter | 0.01 | 0 | 0 | Hymenobacter aerophilus | 0 | 0 | 53/4253647 | 0.0012 | 1 |
| Hyunsoonleella | 0.02 | 0 | 0 | Hyunsoonleella jejuensis | 0 | 0 | 68/3480411 | 0.002 | 1 |
| Ideonella | 0.06 | 0 | 0 | Ideonella sakaiensis | 0 | 0 | 376/6144323 | 0.0061 | 1 |
| Inquilinus | 0.03 | 0 | 0 | Inquilinus limosus | 0 | 0 | 217/7414344 | 0.0029 | 1 |
| Jannaschia | 0.03 | 0 | 0 | Jannaschia CCS1 | 0 | 0 | 85/4317977 | 0.002 | 1 |
| Jiangella | 0.01 | 0 | 0 | Jiangella alkaliphila | 0 | 0 | 67/7716600 | 0.0009 | 1 |
| Kaistia | 0.04 | 0 | 0 | Kaistia adipata | 0 | 0 | 178/4839313 | 0.0037 | 1 |
| Kineosphaera | 0.01 | 0 | 0 | Kineosphaera limosa | 0 | 0 | 64/4848887 | 0.0013 | 1 |
| Kinetoplastibacterium | 0.08 | 0 | 0 | Candidatus Kinetoplastibacterium blastocrithidii | 0 | 0 | 44/820037 | 0.0054 | 1 |
| Kitasatospora | 0.01 | 0 | 0 | Kitasatospora setae | 0 | 0 | 72/8783278 | 0.0008 | 1 |
| Klebsiella | 0.01 | 0 | 0 | Klebsiella pneumoniae | 0 | 0 | 45/5574202 | 0.0008 | 1 |
| Kluyvera | 0.01 | 0 | 0 | Kluyvera ascorbata | 0 | 0 | 54/4987442 | 0.0011 | 1 |
| Kordiimonas | 0.02 | 0 | 0 | Kordiimonas gwangyangensis | 0 | 0 | 71/4082365 | 0.0017 | 1 |
| Krasilnikoviella | 0.01 | 0 | 0 | Krasilnikoviella flava | 0 | 0 | 69/4846106 | 0.0014 | 1 |
| Kribbia | 0.02 | 0 | 0 | Kribbia dieselivorans | 0 | 0 | 75/3296992 | 0.0023 | 1 |
| Labrenzia | 0.01 | 0 | 0 | Labrenzia aggregata | 0 | 0 | 57/6149397 | 0.0009 | 1 |
| Lacinutrix | 0.02 | 0 | 0 | Lacinutrix 5H | 0 | 0 | 56/3296168 | 0.0017 | 1 |
| Lacunisphaera | 0.01 | 0 | 0 | Lacunisphaera limnophila | 0 | 0 | 47/4199284 | 0.0011 | 1 |
| Lampropedia | 0.02 | 0 | 0 | Lampropedia cohaerens | 0 | 0 | 49/3159322 | 0.0016 | 1 |
| Leeia | 0.02 | 0 | 0 | Leeia oryzae | 0 | 0 | 50/3785675 | 0.0013 | 1 |
| Legionella | 0.03 | 0 | 0 | Legionella birminghamensis | 0 | 0 | 61/3738185 | 0.0016 | 1 |
| Legionella | 0.03 | 0 | 0 | Legionella feeleii | 0 | 0 | 45/3555543 | 0.0013 | 1 |
| Leifsonia | 0.08 | 0 | 0 | Leifsonia aquatica | 0 | 0 | 101/4235539 | 0.0024 | 1 |
| Leifsonia | 0.08 | 0 | 0 | Leifsonia xyli | 0 | 0 | 118/2686418 | 0.0044 | 1 |
| Leisingera | 0.03 | 0 | 0 | Leisingera daeponensis | 0 | 0 | 44/4642636 | 0.0009 | 1 |
| Leisingera | 0.03 | 0 | 0 | Leisingera methylohalidivorans | 0 | 0 | 68/4144900 | 0.0016 | 1 |
| Leptothrix | 0.06 | 0 | 0 | Leptothrix cholodnii | 0 | 0 | 271/4909403 | 0.0055 | 1 |
| Limimonas | 0.02 | 0 | 0 | Limimonas halophila | 0 | 0 | 51/3039611 | 0.0017 | 1 |
| Luteibacter | 0.03 | 0 | 0 | Luteibacter rhizovicinus | 0 | 0 | 118(/4765486 | 0.0025 | 1 |
| Luteipulveratus | 0.01 | 0 | 0 | Luteipulveratus halotolerans | 0 | 0 | 75/4464306 | 0.0017 | 1 |
| Lysinimicrobium | 0.02 | 0 | 0 | Lysinimicrobium flavum | 0 | 0 | 59/2985075 | 0.002 | 1 |
| Lysobacter | 0.05 | 0 | 0 | Lysobacter antibioticus | 0 | 0 | 278/5916388 | 0.0047 | 1 |
| Magnetospirillum | 0.01 | 0 | 0 | Magnetospirillum magneticum | 0 | 0 | 64/4967148 | 0.0013 | 1 |
| Mangrovimonas | 0.02 | 0 | 0 | Mangrovimonas yunxiaonensis | 0 | 0 | 65/2670133 | 0.0024 | 1 |
| Marinilabilia | 0.01 | 0 | 0 | Marinilabilia salmonicolor | 0 | 0 | 45/4791112 | 0.0009 | 1 |
| Marinovum | 0.02 | 0 | 0 | Marinovum algicola | 0 | 0 | 62/3727131 | 0.0017 | 1 |
| Maritimibacter | 0.01 | 0 | 0 | Maritimibacter alkaliphilus | 0 | 0 | 75/4536173 | 0.0017 | 1 |
| Marmoricola | 0.01 | 0 | 0 | Marmoricola aequoreus | 0 | 0 | 75/4163916 | 0.0018 | 1 |
| Martelella | 0.03 | 0 | 0 | Martelella endophytica | 0 | 0 | 108/4817335 | 0.0022 | 1 |
| Mastigocladus | 0.01 | 0 | 0 | Mastigocladus laminosus | 0 | 0 | 52/8561912 | 0.0006 | 1 |
| Metallibacterium | 0.06 | 0 | 0 | Metallibacterium scheffleri | 0 | 0 | 185/3328497 | 0.0056 | 1 |
| Methylobacillus | 0.02 | 0 | 0 | Methylobacillus flagellatus | 0 | 0 | 65/2971517 | 0.0022 | 1 |
| Methylobrevis | 0.03 | 0 | 0 | Methylobrevis pamukkalensis | 0 | 0 | 106/4384163 | 0.0024 | 1 |
| Methyloferula | 0.03 | 0 | 0 | Methyloferula stellata | 0 | 0 | 121/4235448 | 0.0029 | 1 |
| Methyloligella | 0.02 | 0 | 0 | Methyloligella halotolerans | 0 | 0 | 42/3191878 | 0.0013 | 1 |
| Methylosarcina | 0.01 | 0 | 0 | Methylosarcina fibrata | 0 | 0 | 75/4978474 | 0.0015 | 1 |
| Methylovorus | 0.08 | 0 | 0 | Methylovorus glucosetrophus | 0 | 0 | 200/2995511 | 0.0067 | 1 |
| Methylovorus | 0.08 | 0 | 0 | Methylovorus MP688 | 0 | 0 | 42/2862391 | 0.0015 | 1 |
| Microvirga | 0.06 | 0 | 0 | Microvirga flocculans | 0 | 0 | 199/4030071 | 0.0049 | 1 |
| Millisia | 0.02 | 0 | 0 | Millisia brevis | 0 | 0 | 120/5646860 | 0.0021 | 1 |
| Mitsuaria | 0.1 | 0 | 0 | Mitsuaria chitosanitabida | 0 | 0 | 518/5819963 | 0.0089 | 1 |
| Mizugakiibacter | 0.08 | 0 | 0 | Mizugakiibacter sediminis | 0 | 0 | 218/312278 | 0.007 | 1 |
| Modestobacter | 0.06 | 0 | 0 | Modestobacter marinus | 0 | 0 | 340/5575517 | 0.0061 | 1 |
| Monashia | 0.01 | 0 | 0 | Monashia flava | 0 | 0 | 70/4245965 | 0.0016 | 1 |
| Nakamurella | 0.01 | 0 | 0 | Nakamurella multipartita | 0 | 0 | 61/6060298 | 0.001 | 1 |
| Niastella | 0.01 | 0 | 0 | Niastella koreensis | 0 | 0 | 108/9033684 | 0.0012 | 1 |
| Nitrobacter | 0.07 | 0 | 0 | Nitrobacter hamburgensis | 0 | 0 | 344/4406967 | 0.0078 | 1 |
| Nitrosomonas | 0.02 | 0 | 0 | Nitrosomonas Is79A3 | 0 | 0 | 59/3783444 | 0.0016 | 1 |
| Nitrosospira | 0.02 | 0 | 0 | Nitrosospira multiformis | 0 | 0 | 53/3184243 | 0.0017 | 1 |
| Nitrospira | 0.01 | 0 | 0 | Candidatus Nitrospira defluvii | 0 | 0 | 56/4317083 | 0.0013 | 1 |
| Nocardia | 0.01 | 0 | 0 | Nocardia brasiliensis | 0 | 0 | 44/9436348 | 0.0005 | 1 |
| Nocardia | 0.01 | 0 | 0 | Nocardia vulneris | 0 | 0 | 66/9378340 | 0.0007 | 1 |
| Nocardiopsis | 0.01 | 0 | 0 | Nocardiopsis synnemataformans | 0 | 0 | 49/7362964 | 0.0007 | 1 |
| Noviherbaspirillum | 0.09 | 0 | 0 | Herbaspirillum massiliense | 0 | 0 | 383/4188289 | 0.0091 | 1 |
| Oceanimonas | 0.02 | 0 | 0 | Oceanimonas GK1 | 0 | 0 | 38/3514537 | 0.0011 | 1 |
| Odoribacter | 0.01 | 0 | 0 | Odoribacter splanchnicus | 0 | 0 | 66/4392288 | 0.0015 | 1 |
| Oligotropha | 0.1 | 0 | 0 | Oligotropha carboxidovorans | 0 | 0 | 430/3745629 | 0.0115 | 1 |
| Oxalobacter | 0.02 | 0 | 0 | Oxalobacter formigenes | 0 | 0 | 44/2488396 | 0.0018 | 1 |
| Paenibacillus | 0.02 | 0 | 0 | Paenibacillus alvei | 0 | 0 | 36/6553920 | 0.0005 | 1 |
| Paenibacillus | 0.02 | 0 | 0 | Paenibacillus macerans | 0 | 0 | 39/7337750 | 0.0005 | 1 |
| Pantoea | 0.04 | 0 | 0 | Pantoea dispersa | 0 | 0 | 221/4951455 | 0.0045 | 1 |
| Paracoccus | 0.06 | 0 | 0 | Paracoccus aminophilus | 0 | 0 | 46/3613807 | 0.0013 | 1 |
| Paracoccus | 0.06 | 0 | 0 | Paracoccus denitrificans | 0 | 0 | 47/4582379 | 0.001 | 1 |
| Paracoccus | 0.06 | 0 | 0 | Paracoccus versutus | 0 | 0 | 75/5502608 | 0.0014 | 1 |
| Paracoccus | 0.06 | 0 | 0 | Paracoccus yeei | 0 | 0 | 43/4429585 | 0.001 | 1 |
| Pasteurella | 0.02 | 0 | 0 | Pasteurella testudinis | 0 | 0 | 63/2919413 | 0.0022 | 1 |
| Pedobacter | 0.01 | 0 | 0 | Pedobacter saltans | 0 | 0 | 42/4635236 | 0.0009 | 1 |
| Phaeobacter | 0.02 | 0 | 0 | Phaeobacter gallaeciensis | 0 | 0 | 42/3821831 | 0.0011 | 1 |
| Phenylobacterium | 0.05 | 0 | 0 | Phenylobacterium zucineum | 0 | 0 | 164/3996255 | 0.0041 | 1 |
| Polaromonas | 0.1 | 0 | 0 | Polaromonas JS666 | 0 | 0 | 65/5200264 | 0.0012 | 1 |
| Polaromonas | 0.1 | 0 | 0 | Polaromonas naphthalenivorans | 0 | 0 | 385/4410291 | 0.0087 | 1 |
| Polymorphum | 0.01 | 0 | 0 | Polymorphum gilvum | 0 | 0 | 45/4649365 | 0.001 | 1 |
| Proteus | 0.02 | 0 | 0 | Proteus hauseri | 0 | 0 | 40/3784062 | 0.0011 | 1 |
| Pseudoalteromonas | 0.02 | 0 | 0 | Pseudoalteromonas atlantica | 0 | 0 | 115/5187005 | 0.0022 | 1 |
| Pseudogulbenkiania | 0.01 | 0 | 0 | Pseudogulbenkiania NH8B | 0 | 0 | 35/4332995 | 0.0008 | 1 |
| Pseudoramibacter | 0.03 | 0 | 0 | Pseudoramibacter alactolyticus | 0 | 0 | 75/2363292 | 0.0032 | 1 |
| Psychromonas | 0.01 | 0 | 0 | Psychromonas ingrahamii | 0 | 0 | 47/4559598 | 0.001 | 1 |
| Pyramidobacter | 0.02 | 0 | 0 | Pyramidobacter piscolens | 0 | 0 | 54/2563404 | 0.0021 | 1 |
| Ramlibacter | 0.12 | 0 | 0 | Ramlibacter tataouinensis | 0 | 0 | 393/4070193 | 0.0097 | 1 |
| Rhodobacter | 0.3 | 0 | 0 | Rhodanobacter 2APBS1 | 0 | 0 | 1060/4225490 | 0.0251 | 1 |
| Rhodobacter | 0.3 | 0 | 0 | Rhodobacter capsulatus | 0 | 0 | 81/3738958 | 0.0022 | 1 |
| Rhodobacter | 0.3 | 0 | 0 | Rhodobacter sphaeroides | 0 | 0 | 41/4450439 | 0.0009 | 1 |
| Rhodoferax | 0.04 | 0 | 0 | Rhodoferax ferrireducens | 0 | 0 | 192/4712337 | 0.0041 | 1 |
| Rhodomicrobium | 0.02 | 0 | 0 | Rhodomicrobium vannielii | 0 | 0 | 75/4014469 | 0.0019 | 1 |
| Rhodospirillum | 0.02 | 0 | 0 | Rhodospirillum photometricum | 0 | 0 | 47/3876289 | 0.0012 | 1 |
| Rubrivivax | 0.09 | 0 | 0 | Rubrivivax gelatinosus | 0 | 0 | 448/5043253 | 0.0089 | 1 |
| Ruegeria | 0.02 | 0 | 0 | Ruegeria pomeroyi | 0 | 0 | 46/4109437 | 0.0011 | 1 |
| Ruminococcus | 0.02 | 0 | 0 | Ruminococcus sp SR1 5 | 0 | 0 | 75/3545606 | 0.0021 | 1 |
| Sanguibacter | 0.01 | 0 | 0 | Sanguibacter keddieii | 0 | 0 | 39/4253413 | 0.0009 | 1 |
| Serinicoccus | 0.02 | 0 | 0 | Serinicoccus chungangensis | 0 | 0 | 67/3557308 | 0.0019 | 1 |
| Serratia | 0.02 | 0 | 0 | Serratia marcescens | 0 | 0 | 147/5471439 | 0.0027 | 1 |
| Sideroxydans | 0.04 | 0 | 0 | Sideroxydans lithotrophicus | 0 | 0 | 92/3003656 | 0.0031 | 1 |
| Sodalis | 0.01 | 0 | 0 | Sodalis glossinidius | 0 | 0 | 75/4171146 | 0.0018 | 1 |
| Sphingobacterium | 0.01 | 0 | 0 | Sphingobacterium spiritivorum | 0 | 0 | 55/5233338 | 0.0011 | 1 |
| Spiribacter | 0.04 | 0 | 0 | Ectothiorhodospiraceae bacterium | 0 | 0 | 51/1739487 | 0.0029 | 1 |
| Starkeya | 0.01 | 0 | 0 | Starkeya novella | 0 | 0 | 75/4765023 | 0.0016 | 1 |
| Streptomyces | 0.01 | 0 | 0 | Streptomyces venezuelae | 0 | 0 | 49/8226158 | 0.0006 | 1 |
| Sutterella | 0.03 | 0 | 0 | Sutterella parvirubra | 0 | 0 | 64/2378769 | 0.0027 | 1 |
| Taylorella | 0.07 | 0 | 0 | Taylorella equigenitalis | 0 | 0 | 97/1732123 | 0.0056 | 1 |
| Thauera | 0.01 | 0 | 0 | Thauera MZ1T | 0 | 0 | 62/4496212 | 0.0014 | 1 |
| Thermotoga | 0.03 | 0 | 0 | Thermotoga hypogea | 0 | 0 | 75/2165165 | 0.0035 | 1 |
| Thioalkalivibrio | 0.02 | 0 | 0 | Thioalkalivibrio K90mix | 0 | 0 | 70/2744800 | 0.0026 | 1 |
| Thioflavicoccus | 0.02 | 0 | 0 | Thioflavicoccus mobilis | 0 | 0 | 42/4048921 | 0.001 | 1 |
| Treponema | 0.02 | 0 | 0 | Treponema azotonutricium | 0 | 0 | 58/3855671 | 0.0015 | 1 |
| Truepera | 0.02 | 0 | 0 | Truepera radiovictrix | 0 | 0 | 62/3260398 | 0.0019 | 1 |
| Tsukamurella | 0.01 | 0 | 0 | Tsukamurella pulmonis | 0 | 0 | 45/4783074 | 0.0009 | 1 |
| Tyzzerella | 0.02 | 0 | 0 | Tyzzerella nexilis | 0 | 0 | 75/3996608 | 0.0019 | 1 |
| Variovorax | 0.04 | 0 | 0 | Variovorax paradoxus | 0 | 0 | 313/7148516 | 0.0044 | 1 |
| Verrucosispora | 0.01 | 0 | 0 | Verrucosispora maris | 0 | 0 | 36/6673976 | 0.0005 | 1 |
| Vibrio | 0.01 | 0 | 0 | Vibrio Ex25 | 0 | 0 | 50/5089025 | 0.001 | 1 |
| Xylanimonas | 0.02 | 0 | 0 | Xylanimonas cellulosilytica | 0 | 0 | 69/3742776 | 0.0018 | 1 |
| Xylella | 0.02 | 0 | 0 | Xylella fastidiosa | 0 | 0 | 70/2679306 | 0.0026 | 1 |

Note: Genus Re Abu: Relative abundance of Genus; SMRNG: Stringent mapped reads number of genus; SDSMRNG: Standard Stringent mapped reads number of genus; SMRN: Stringent mapped reads number; SDSMRN: Standard Stringent mapped reads number; CovRate: Coverage rate.

　　 Table 2. Microbe reads of fungi, parasite and virus detected in Case No. 3

| Genus | Genus Re Abu | SMRNG | SDSMRNG | Species | SMRN | SDSMRN | Coverage | CovRate | Depth |
| --- | --- | --- | --- | --- | --- | --- | --- | --- | --- |
| Alternaria | 1.67 | 1 | 2 | Alternaria alternata | 1 | 2 | 75/33021769 | 0.0002 | 1 |
| Candida | 3.77 | 1 | 2 | Candida tropicalis | 1 | 2 | 49/14630369 | 0.0003 | 1 |
| Coprinopsis | 1.52 | 1 | 2 | Coprinopsis cinerea | 1 | 2 | 53/36193260 | 0.0001 | 1 |
| Debaryomyces | 4.52 | 1 | 2 | Debaryomyces hansenii | 1 | 2 | 54/12182018 | 0.0004 | 1 |
| Penicillium | 10.22 | 2 | 3 | Penicillium citrinum | 1 | 2 | 43/33998688 | 0.0001 | 1 |
| Phanerochaete | 1.85 | 1 | 2 | Phanerochaete chrysosporium | 1 | 2 | 44/29855776 | 0.0001 | 1 |
| Pythium | 5.38 | 1 | 2 | Pythium irregulare | 1 | 2 | 192/43026944 | 0.0004 | 1 |
| Rhodotorula | 2.76 | 1 | 2 | Rhodotorula mucilaginosa | 1 | 2 | 73/19992149 | 0.0004 | 1 |
| Sordaria | 23.42 | 1 | 2 | Sordaria macrospora | 1 | 2 | 1150/40002837 | 0.0029 | 1 |
| Albugo | 3.36 | 0 | 0 | Albugo laibachii | 0 | 0 | 145/32805071 | 0.0004 | 1 |
| Eremothecium | 6.05 | 0 | 0 | Eremothecium gossypii | 0 | 0 | 67/9119382 | 0.0007 | 1 |
| Hyaloperonospora | 0.71 | 0 | 0 | Hyaloperonospora arabidopsidis | 0 | 0 | 67/78411605 | 0.0001 | 1 |
| Lachancea | 5.05 | 0 | 0 | Lachancea waltii | 0 | 0 | 40/10919232 | 0.0004 | 1 |
| Malassezia | 19.2 | 0 | 0 | Malassezia globosa | 0 | 0 | 124/8872979 | 0.0014 | 1 |
| Malassezia | 19.2 | 0 | 0 | Malassezia pachydermatis | 0 | 0 | 72/8129607 | 0.0009 | 1 |
| Naumovozyma | 4.07 | 0 | 0 | Naumovozyma dairenensis | 0 | 0 | 55/13527680 | 0.0004 | 1 |
| Penicillium | 10.22 | 2 | 3 | Penicillium chrysogenum | 0 | 0 | 71/32525581 | 0.0002 | 1 |
| Penicillium | 10.22 | 2 | 3 | Penicillium expansum | 0 | 0 | 146/32907681 | 0.0004 | 1 |
| Penicillium | 10.22 | 2 | 3 | Penicillium italicum | 0 | 0 | 147/31030153 | 0.0005 | 1 |
| Pythium | 5.38 | 1 | 2 | Pythium aphanidermatum | 0 | 0 | 57/35894579 | 0.0002 | 1 |
| Saccharomyces | 4.54 | 0 | 0 | Saccharomyces cerevisiae | 0 | 0 | 67/12157265 | 0.0006 | 1 |
| Talaromyces | 1.92 | 0 | 0 | Talaromyces marneffei | 0 | 0 | 53/28648375 | 0.0002 | 1 |
| Wuchereria | 9.02 | 7 | 11 | Wuchereria bancrofti | 7 | 11 | 475/77159569 | 0.0006 | 1.19 |
| Gongylonema | 2.69 | 6 | 10 | Gongylonema pulchrum | 6 | 10 | 493/290924066 | 0.0002 | 1.26 |
| Acanthamoeba | 78.73 | 9 | 15 | Acanthamoeba mauritaniensis | 2 | 3 | 949/96417684 | 0.001 | 1 |
| Fasciola | 0.07 | 1 | 2 | Fasciola hepatica | 1 | 2 | 41/1185539267 | 0 | 1 |
| Acanthamoeba | 78.73 | 9 | 15 | Acanthamoeba palestinensis | 0 | 0 | 1982/93383381 | 0.0021 | 1 |
| Acanthamoeba | 78.73 | 9 | 15 | Acanthamoeba polyphaga | 0 | 0 | 118/109518714 | 0.0001 | 1 |
| Acanthamoeba | 78.73 | 9 | 15 | Acanthamoeba triangularis | 0 | 0 | 2413/85627420 | 0.0028 | 1 |
| Eimeria | 2.11 | 0 | 0 | Eimeria acervulina | 0 | 0 | 62/41265254 | 0.0002 | 1 |
| Plasmodium | 4.67 | 0 | 0 | Plasmodium reichenowi | 0 | 0 | 68/18638506 | 0.0004 | 1 |
| Spirometra | 0.16 | 0 | 0 | Spirometra erinaceieuropaei | 0 | 0 | 109/1144239852 | 0 | 1 |
| Trichuris | 2.56 | 0 | 0 | Trichuris trichiura | 0 | 0 | 110/67961556 | 0.0002 | 1 |
| - | - | - | - | Human alphaherpesvirus 3 (Varicella zoster virus) | 811 | 1330 | 43424/124884 | 34.77 | 1.25 |

Note: Genus Re Abu: Relative abundance of Genus; SMRNG: Stringent mapped reads number of genus; SDSMRNG: Standard Stringent mapped reads number of genus; SMRN: Stringent mapped reads number; SDSMRN: Standard Stringent mapped reads number; CovRate: Coverage rate.
