# Supplementary material for: Clinical features of aseptic meningitis with varicella zoster virus infection diagnosed by next-generation sequencing: case reports
Source: BMC Infect Dis. 2020 Jun 22;20:435. doi: 10.1186/s12879-020-05155-8 (PMC7309994; doi:10.1186/s12879-020-05155-8)
Supplement: Supplementary file 5 — Additional file 5. Microbe reads of bacterium, fungi, parasite and virus detected in Case No. 4 [file 12879_2020_5155_MOESM5_ESM.docx]

**Additional file 5:** Microbe reads of bacterium, fungi, parasite and virus detected in Case No. 4

　Table 1. Microbe reads of bacterium detected in Case No. 4

| Genus | Genus Re Abu | SMRNG | SDSMRNG | Species | SMRN | SDSMRN | Coverage | CovRate | Depth |
| --- | --- | --- | --- | --- | --- | --- | --- | --- | --- |
| Propionibacterium | 27.26 | 294 | 356 | Propionibacterium acnes | 273 | 331 | 29096/2560282 | 1.14 | 1.01 |
| Propionibacterium | 27.26 | 294 | 356 | Propionibacterium humerusii | 1 | 1 | 715/2644116 | 0.027 | 1 |
| Propionibacterium | 27.26 | 294 | 356 | Propionibacterium acidifaciens | 0 | 0 | 64/3044741 | 0.0021 | 1 |
| Staphylococcus | 28.95 | 258 | 313 | Staphylococcus epidermidis | 177 | 214 | 16355/2616530 | 0.6251 | 1.01 |
| Staphylococcus | 28.95 | 258 | 313 | Staphylococcus hominis | 49 | 59 | 5633/1140916 | 0.4937 | 1 |
| Staphylococcus | 28.95 | 258 | 313 | Staphylococcus capitis | 10 | 12 | 1295/2466594 | 0.0525 | 1 |
| Staphylococcus | 28.95 | 258 | 313 | Staphylococcus haemolyticus | 3 | 4 | 515/2685015 | 0.0192 | 1 |
| Staphylococcus | 28.95 | 258 | 313 | Staphylococcus warneri | 1 | 1 | 353/2486042 | 0.0142 | 1 |
| Staphylococcus | 28.95 | 258 | 313 | Staphylococcus argenteus | 0 | 0 | 75/2762785 | 0.0027 | 1 |
| Staphylococcus | 28.95 | 258 | 313 | Staphylococcus aureus | 0 | 0 | 71/2898306 | 0.0024 | 1 |
| Staphylococcus | 28.95 | 258 | 313 | Staphylococcus cohnii | 0 | 0 | 145/2677922 | 0.0054 | 1 |
| Staphylococcus | 28.95 | 258 | 313 | Staphylococcus equorum | 0 | 0 | 124/2753539 | 0.0045 | 1 |
| Staphylococcus | 28.95 | 258 | 313 | Staphylococcus gallinarum | 0 | 0 | 65/3174430 | 0.002 | 1 |
| Staphylococcus | 28.95 | 258 | 313 | Staphylococcus pasteuri | 0 | 0 | 217/2559946 | 0.0085 | 1 |
| Sphingomonas | 3.29 | 52 | 63 | Sphingomonas melonis | 29 | 35 | 2513/4156476 | 0.0605 | 1 |
| Sphingomonas | 3.29 | 52 | 63 | Sphingomonas parapaucimobilis | 16 | 19 | 2063/3995782 | 0.0516 | 1 |
| Sphingomonas | 3.29 | 52 | 63 | Sphingomonas echinoides | 4 | 5 | 538/4264986 | 0.0126 | 1 |
| Sphingomonas | 3.29 | 52 | 63 | Sphingomonas paucimobilis | 1 | 1 | 37/4874985 | 0.0008 | 1 |
| Sphingomonas | 3.29 | 52 | 63 | Sphingomonas adhaesiva | 0 | 0 | 310/4026130 | 0.0077 | 1 |
| Sphingomonas | 3.29 | 52 | 63 | Sphingomonas MM | 0 | 0 | 61/4054833 | 0.0015 | 1 |
| Corynebacterium | 5 | 41 | 50 | Corynebacterium tuberculostearicum | 5 | 6 | 2384/2372621 | 0.1005 | 1 |
| Corynebacterium | 5 | 41 | 50 | Corynebacterium pseudogenitalium | 2 | 2 | 2198/2601506 | 0.0845 | 1 |
| Corynebacterium | 5 | 41 | 50 | Corynebacterium jeddahense | 1 | 1 | 75/2474555 | 0.003 | 1 |
| Corynebacterium | 5 | 41 | 50 | Corynebacterium matruchotii | 1 | 1 | 75/2992345 | 0.0025 | 1 |
| Corynebacterium | 5 | 41 | 50 | Corynebacterium striatum | 1 | 1 | 202/2915083 | 0.0069 | 1 |
| Corynebacterium | 5 | 41 | 50 | Corynebacterium afermentans | 0 | 0 | 150/2345845 | 0.0064 | 1 |
| Corynebacterium | 5 | 41 | 50 | Corynebacterium aurimucosum | 0 | 0 | 63/2790189 | 0.0023 | 1 |
| Corynebacterium | 5 | 41 | 50 | Corynebacterium massiliense | 0 | 0 | 61/2183269 | 0.0028 | 1 |
| Corynebacterium | 5 | 41 | 50 | Corynebacterium resistens | 0 | 0 | 144/2601311 | 0.0055 | 1 |
| Corynebacterium | 5 | 41 | 50 | Corynebacterium simulans | 0 | 0 | 66/2737971 | 0.0024 | 1 |
| Burkholderia | 2.16 | 30 | 36 | Burkholderia glumae | 1 | 1 | 326/6733840 | 0.0048 | 1 |
| Burkholderia | 2.16 | 30 | 36 | Burkholderia phytofirmans | 1 | 1 | 135/8093536 | 0.0017 | 1 |
| Burkholderia | 2.16 | 30 | 36 | Burkholderia stabilis | 1 | 1 | 299/8527967 | 0.0035 | 1 |
| Burkholderia | 2.16 | 30 | 36 | Burkholderia ambifaria | 0 | 0 | 178/7484986 | 0.0024 | 1 |
| Burkholderia | 2.16 | 30 | 36 | Burkholderia anthina | 0 | 0 | 244/7273081 | 0.0034 | 1 |
| Burkholderia | 2.16 | 30 | 36 | Burkholderia CCGE1001 | 0 | 0 | 75/6833751 | 0.0011 | 1 |
| Burkholderia | 2.16 | 30 | 36 | Burkholderia CCGE1002 | 0 | 0 | 75/7395722 | 0.001 | 1 |
| Burkholderia | 2.16 | 30 | 36 | Burkholderia CCGE1003 | 0 | 0 | 50/7043595 | 0.0007 | 1 |
| Burkholderia | 2.16 | 30 | 36 | Burkholderia cenocepacia | 0 | 0 | 211/8531679 | 0.0025 | 1 |
| Burkholderia | 2.16 | 30 | 36 | Burkholderia cepacia | 0 | 0 | 174/8396158 | 0.0021 | 1 |
| Burkholderia | 2.16 | 30 | 36 | Burkholderia diffusa | 0 | 0 | 132/6857853 | 0.0019 | 1 |
| Burkholderia | 2.16 | 30 | 36 | Burkholderia dolosa | 0 | 0 | 264/6409115 | 0.0041 | 1 |
| Burkholderia | 2.16 | 30 | 36 | Burkholderia gladioli | 0 | 0 | 55/8114449 | 0.0007 | 1 |
| Burkholderia | 2.16 | 30 | 36 | Burkholderia KJ006 | 0 | 0 | 139/6584551 | 0.0021 | 1 |
| Burkholderia | 2.16 | 30 | 36 | Burkholderia lata | 0 | 0 | 303/8676277 | 0.0035 | 1 |
| Burkholderia | 2.16 | 30 | 36 | Burkholderia latens | 0 | 0 | 215/6569017 | 0.0033 | 1 |
| Burkholderia | 2.16 | 30 | 36 | Burkholderia mallei | 0 | 0 | 76/5913144 | 0.0013 | 1 |
| Burkholderia | 2.16 | 30 | 36 | Burkholderia metallica | 0 | 0 | 60/7424260 | 0.0008 | 1 |
| Burkholderia | 2.16 | 30 | 36 | Burkholderia multivorans | 0 | 0 | 272/7281887 | 0.0037 | 1 |
| Burkholderia | 2.16 | 30 | 36 | Burkholderia oklahomensis | 0 | 0 | 113/7313683 | 0.0015 | 1 |
| Burkholderia | 2.16 | 30 | 36 | Burkholderia phenoliruptrix | 0 | 0 | 169/6865712 | 0.0025 | 1 |
| Burkholderia | 2.16 | 30 | 36 | Burkholderia phymatum | 0 | 0 | 75/6176561 | 0.0012 | 1 |
| Burkholderia | 2.16 | 30 | 36 | Burkholderia pseudomallei | 0 | 0 | 51/7446579 | 0.0007 | 1 |
| Burkholderia | 2.16 | 30 | 36 | Burkholderia pyrrocinia | 0 | 0 | 241/7847882 | 0.0031 | 1 |
| Burkholderia | 2.16 | 30 | 36 | Burkholderia RPE64 | 0 | 0 | 75/5379596 | 0.0014 | 1 |
| Burkholderia | 2.16 | 30 | 36 | Burkholderia thailandensis | 0 | 0 | 240/6739510 | 0.0036 | 1 |
| Burkholderia | 2.16 | 30 | 36 | Burkholderia ubonensis | 0 | 0 | 1046/8028779 | 0.013 | 1 |
| Burkholderia | 2.16 | 30 | 36 | Burkholderia vietnamiensis | 0 | 0 | 176/6827896 | 0.0026 | 1 |
| Burkholderia | 2.16 | 30 | 36 | Burkholderia xenovorans | 0 | 0 | 227/9731138 | 0.0023 | 1 |
| Mycoplasma | 8.19 | 26 | 31 | Mycoplasma wenyonii | 26 | 31 | 2130/650228 | 0.3276 | 1 |
| Mycoplasma | 8.19 | 26 | 31 | Mycoplasma ovis | 0 | 0 | 65/702511 | 0.0093 | 1 |
| Delftia | 0.83 | 25 | 30 | Delftia tsuruhatensis | 4 | 5 | 697/7195716 | 0.0097 | 1 |
| Delftia | 0.83 | 25 | 30 | Delftia acidovorans | 2 | 2 | 985/6767514 | 0.0146 | 1 |
| Delftia | 0.83 | 25 | 30 | Delftia Cs1 | 1 | 1 | 746/6685842 | 0.0112 | 1 |
| Pseudomonas | 0.86 | 23 | 28 | Pseudomonas putida | 19 | 23 | 1331/6377271 | 0.0209 | 1 |
| Pseudomonas | 0.86 | 23 | 28 | Pseudomonas alcaligenes | 2 | 2 | 115/4406305 | 0.0026 | 1 |
| Pseudomonas | 0.86 | 23 | 28 | Pseudomonas resinovorans | 1 | 1 | 75/6285863 | 0.0012 | 1 |
| Pseudomonas | 0.86 | 23 | 28 | Pseudomonas fluorescens | 0 | 0 | 40/6136735 | 0.0007 | 1 |
| Pseudomonas | 0.86 | 23 | 28 | Pseudomonas monteilii | 0 | 0 | 75/6000087 | 0.0012 | 1 |
| Pseudomonas | 0.86 | 23 | 28 | Pseudomonas poae | 0 | 0 | 95/5512241 | 0.0017 | 1 |
| Pseudomonas | 0.86 | 23 | 28 | Pseudomonas stutzeri | 0 | 0 | 113/4650155 | 0.0024 | 1 |
| Pseudomonas | 0.86 | 23 | 28 | Pseudomonas syringae | 0 | 0 | 75/6665031 | 0.0011 | 1 |
| Pseudomonas | 0.86 | 23 | 28 | Pseudomonas veronii | 0 | 0 | 144/6649830 | 0.0022 | 1 |
| Moraxella | 2.05 | 21 | 25 | Moraxella osloensis | 21 | 25 | 2021/2434688 | 0.083 | 1.01 |
| Acinetobacter | 1.12 | 18 | 22 | Acinetobacter johnsonii | 16 | 19 | 1415/3509795 | 0.0403 | 1 |
| Acinetobacter | 1.12 | 18 | 22 | Acinetobacter bereziniae | 1 | 1 | 75/5026552 | 0.0015 | 1 |
| Acinetobacter | 1.12 | 18 | 22 | Acinetobacter junii | 1 | 1 | 143/3782588 | 0.0038 | 1 |
| Acinetobacter | 1.12 | 18 | 22 | Acinetobacter haemolyticus | 0 | 0 | 75/3715798 | 0.002 | 1 |
| Campylobacter | 1.71 | 18 | 22 | Campylobacter mucosalis | 18 | 22 | 778/1752144 | 0.0444 | 1.61 |
| Acidovorax | 1.56 | 15 | 18 | Acidovorax KKS102 | 15 | 18 | 2664/5196935 | 0.0513 | 1 |
| Acidovorax | 1.56 | 15 | 18 | Acidovorax avenae | 0 | 0 | 39/5482170 | 0.0007 | 1 |
| Acidovorax | 1.56 | 15 | 18 | Acidovorax citrulli | 0 | 0 | 100/5352772 | 0.0019 | 1 |
| Acidovorax | 1.56 | 15 | 18 | Acidovorax ebreus | 0 | 0 | 56/3796573 | 0.0015 | 1 |
| Acidovorax | 1.56 | 15 | 18 | Acidovorax JS42 | 0 | 0 | 116/4448856 | 0.0026 | 1 |
| Cupriavidus | 0.79 | 14 | 17 | Cupriavidus metallidurans | 11 | 13 | 956/3928089 | 0.0243 | 1 |
| Cupriavidus | 0.79 | 14 | 17 | Cupriavidus necator | 1 | 1 | 287/6557542 | 0.0044 | 1 |
| Cupriavidus | 0.79 | 14 | 17 | Cupriavidus taiwanensis | 0 | 0 | 53/5919322 | 0.0009 | 1 |
| Methylobacterium | 0.54 | 13 | 16 | Methylobacterium aquaticum | 2 | 2 | 147(5348274 | 0.0027 | 1 |
| Methylobacterium | 0.54 | 13 | 16 | Methylobacterium radiotolerans | 2 | 2 | 345/6077833 | 0.0057 | 1 |
| Methylobacterium | 0.54 | 13 | 16 | Methylobacterium brachiatum | 1 | 1 | 128/5807713 | 0.0022 | 1 |
| Methylobacterium | 0.54 | 13 | 16 | Methylobacterium populi | 1 | 1 | 295/5800441 | 0.0051 | 1 |
| Methylobacterium | 0.54 | 13 | 16 | Methylobacterium chloromethanicum | 0 | 0 | 199/5777908 | 0.0034 | 1 |
| Methylobacterium | 0.54 | 13 | 16 | Methylobacterium extorquens | 0 | 0 | 148/5943768 | 0.0025 | 1 |
| Methylobacterium | 0.54 | 13 | 16 | Methylobacterium mesophilicum | 0 | 0 | 65/6214729 | 0.001 | 1 |
| Methylotenera | 1.21 | 8 | 10 | Methylotenera 301 | 6 | 7 | 893/3059871 | 0.0292 | 1 |
| Methylotenera | 1.21 | 8 | 10 | Methylotenera mobilis | 0 | 0 | 374/2547570 | 0.0147 | 1 |
| Sphingobium | 0.48 | 6 | 7 | Sphingobium xenophagum | 5 | 6 | 383/4487790 | 0.0085 | 1 |
| Sphingobium | 0.48 | 6 | 7 | Sphingobium SYK | 1 | 1 | 35/4199332 | 0.0008 | 1 |
| Sphingobium | 0.48 | 6 | 7 | Sphingobium chlorophenolicum | 0 | 0 | 60/4449488 | 0.0013 | 1 |
| Sphingobium | 0.48 | 6 | 7 | Sphingobium japonicum | 0 | 0 | 191/4196714 | 0.0046 | 1 |
| Sphingobium | 0.48 | 6 | 7 | Sphingobium yanoikuyae | 0 | 0 | 141/5532659 | 0.0025 | 1 |
| Andreesenia | 0.64 | 5 | 6 | Andreesenia angusta | 5 | 6 | 608/2343873 | 0.0259 | 1 |
| Brevundimonas | 0.35 | 5 | 6 | Brevundimonas vesicularis | 4 | 5 | 331/3358839 | 0.0099 | 1 |
| Brevundimonas | 0.35 | 5 | 6 | Brevundimonas diminuta | 0 | 0 | 53/3369386 | 0.0016 | 1 |
| Brevundimonas | 0.35 | 5 | 6 | Brevundimonas subvibrioides | 0 | 0 | 70/3445263 | 0.002 | 1 |
| Mycobacterium | 0.22 | 5 | 6 | Mycobacterium mucogenicum | 2 | 2 | 189/6475901 | 0.0029 | 1 |
| Mycobacterium | 0.22 | 5 | 6 | Mycobacterium llatzerense | 1 | 1 | 125/6700160 | 0.0019 | 1 |
| Mycobacterium | 0.22 | 5 | 6 | Mycobacterium acapulcensis | 0 | 0 | 75/5287170 | 0.0014 | 1 |
| Mycobacterium | 0.22 | 5 | 6 | Mycobacterium diernhoferi | 0 | 0 | 68/5982889 | 0.0011 | 1 |
| Mycobacterium | 0.22 | 5 | 6 | Mycobacterium heckeshornense | 0 | 0 | 49/5012073 | 0.001 | 1 |
| Microbacterium | 0.24 | 4 | 5 | Microbacterium aurum | 4 | 5 | 257/3424892 | 0.0075 | 1 |
| Microbacterium | 0.24 | 4 | 5 | Microbacterium testaceum | 0 | 0 | 36/3982034 | 0.0009 | 1 |
| Acetanaerobacterium | 0.17 | 3 | 4 | Acetanaerobacterium elongatum | 3 | 4 | 225/2916935 | 0.0077 | 1 |
| Cutibacterium | 1.13 | 3 | 4 | Cutibacterium granulosum | 1 | 1 | 147/2141325 | 0.0069 | 1 |
| Cutibacterium | 1.13 | 3 | 4 | Propionibacterium namnetense | 1 | 1 | 933/2369944 | 0.0394 | 1 |
| Cutibacterium | 1.13 | 3 | 4 | Cutibacterium avidum | 0 | 0 | 75/2729848 | 0.0027 | 1 |
| Deinococcus | 0.23 | 3 | 4 | Deinococcus geothermalis | 2 | 2 | 150/2467205 | 0.0061 | 1 |
| Deinococcus | 0.23 | 3 | 4 | Deinococcus gobiensis | 1 | 1 | 51/3137147 | 0.0016 | 1 |
| Deinococcus | 0.23 | 3 | 4 | Deinococcus apachensis | 0 | 0 | 75/4453152 | 0.0017 | 1 |
| Aerococcus | 0.3 | 2 | 2 | Aerococcus viridans | 2 | 2 | 275/2199877 | 0.0125 | 1 |
| Aquamicrobium | 0.14 | 2 | 2 | Aquamicrobium defluvii | 2 | 2 | 260/4775173 | 0.0054 | 1 |
| Bosea | 0.11 | 2 | 2 | Bosea lupini | 2 | 2 | 258/6081108 | 0.0042 | 1 |
| Cytophaga | 0.08 | 2 | 2 | Cytophaga hutchinsonii | 2 | 2 | 124/4433218 | 0.0028 | 1 |
| Ensifer | 0.08 | 2 | 2 | Ensifer adhaerens | 2 | 2 | 150/4071185 | 0.0037 | 1 |
| Enterobacter | 0.07 | 2 | 2 | Enterobacter cloacae complex Hoffmann cluster IV | 1 | 1 | 55/4748414 | 0.0012 | 1 |
| Enterobacter | 0.07 | 2 | 2 | Enterobacter kobei | 1 | 1 | 60/4880257 | 0.0012 | 1 |
| Flavobacterium | 0.41 | 2 | 2 | Flavobacterium aquatile | 1 | 1 | 150/3488993 | 0.0043 | 1 |
| Flavobacterium | 0.41 | 2 | 2 | Flavobacterium degerlachei | 1 | 1 | 75/3856829 | 0.0019 | 1 |
| Flavobacterium | 0.41 | 2 | 2 | Flavobacterium indicum | 0 | 0 | 286/2993089 | 0.0096 | 1 |
| Flavobacterium | 0.41 | 2 | 2 | Flavobacterium succinicans | 0 | 0 | 48/3665414 | 0.0013 | 1 |
| Gordonia | 0.09 | 2 | 2 | Gordonia KTR9 | 1 | 1 | 75/5441391 | 0.0014 | 1 |
| Gordonia | 0.09 | 2 | 2 | Gordonia polyisoprenivorans | 1 | 1 | 75/5669805 | 0.0013 | 1 |
| Gordonia | 0.09 | 2 | 2 | Gordonia bronchialis | 0 | 0 | 75/5208602 | 0.0014 | 1 |
| Klebsiella | 0.06 | 2 | 2 | Klebsiella pneumoniae | 0 | 0 | 75/5574202 | 0.0013 | 1 |
| Klebsiella | 0.06 | 2 | 2 | Klebsiella variicola | 0 | 0 | 59/5521203 | 0.0011 | 1 |
| Kocuria | 0.12 | 2 | 2 | Kocuria palustris | 2 | 2 | 147/2854447 | 0.0051 | 1 |
| Limnohabitans | 0.14 | 2 | 2 | Limnohabitans planktonicus | 2 | 2 | 229/4742314 | 0.0048 | 1 |
| Micrococcus | 0.27 | 2 | 2 | Micrococcus luteus | 2 | 2 | 236/2501097 | 0.0094 | 1 |
| Mitsuaria | 0.11 | 2 | 2 | Mitsuaria chitosanitabida | 2 | 2 | 286/5819963 | 0.0049 | 1 |
| Obesumbacterium | 0.23 | 2 | 2 | Obesumbacterium proteus | 2 | 2 | 478/5011796 | 0.0095 | 1 |
| Ochrobactrum | 0.07 | 2 | 2 | Ochrobactrum anthropi | 1 | 1 | 150/4783208 | 0.0031 | 1 |
| Pannonibacter | 0.09 | 2 | 2 | Pannonibacter phragmitetus | 2 | 2 | 202/5318696 | 0.0038 | 1 |
| Prevotella | 0.15 | 2 | 2 | Prevotella pallens | 2 | 2 | 133/3127990 | 0.0043 | 1 |
| Prevotella | 0.15 | 2 | 2 | Prevotella copri | 0 | 0 | 46/3512733 | 0.0013 | 1 |
| Ralstonia | 0.23 | 2 | 2 | Ralstonia insidiosa | 2 | 2 | 442/5808308 | 0.0076 | 1 |
| Ralstonia | 0.23 | 2 | 2 | Ralstonia mannitolilytica | 0 | 0 | 62/4881769 | 0.0013 | 1 |
| Ralstonia | 0.23 | 2 | 2 | Ralstonia pickettii | 0 | 0 | 40/8125850 | 0.0005 | 1 |
| Rhodococcus | 0.08 | 2 | 2 | Rhodococcus erythropolis | 1 | 1 | 131/6516310 | 0.002 | 1 |
| Rhodococcus | 0.08 | 2 | 2 | Rhodococcus fascians | 1 | 1 | 75/5139988 | 0.0015 | 1 |
| Streptococcus | 0.23 | 2 | 2 | Streptococcus anginosus | 1 | 1 | 75/2233640 | 0.0034 | 1 |
| Streptococcus | 0.23 | 2 | 2 | Streptococcus thermophilus | 1 | 1 | 53/1869510 | 0.0028 | 1 |
| Streptococcus | 0.23 | 2 | 2 | Streptococcus sanguinis | 0 | 0 | 75/2388435 | 0.0031 | 1 |
| Achromobacter | 0.02 | 1 | 1 | Achromobacter xylosoxidans | 1 | 1 | 75/6813182 | 0.0011 | 1 |
| Aeromonas | 0.07 | 1 | 1 | Aeromonas enteropelogenes | 0 | 0 | 75/4475188 | 0.0017 | 1 |
| Aeromonas | 0.07 | 1 | 1 | Aeromonas veronii | 0 | 0 | 75/4923009 | 0.0015 | 1 |
| Afipia | 0.03 | 1 | 1 | Afipia birgiae | 1 | 1 | 49/5334186 | 0.0009 | 1 |
| Alloprevotella | 0.06 | 1 | 1 | Alloprevotella tannerae | 1 | 1 | 75/2585769 | 0.0029 | 1 |
| Aquabacterium | 0.15 | 1 | 1 | Aquabacterium parvum | 1 | 1 | 241/4599179 | 0.0052 | 1 |
| Arcobacter | 0.15 | 1 | 1 | Arcobacter cryaerophilus | 1 | 1 | 52/2210034 | 0.0024 | 1 |
| Arcobacter | 0.15 | 1 | 1 | Arcobacter butzleri | 0 | 0 | 75/2341251 | 0.0032 | 1 |
| Asticcacaulis | 0.04 | 1 | 1 | Asticcacaulis excentricus | 1 | 1 | 75/3904170 | 0.0019 | 1 |
| Bacteroides | 0.05 | 1 | 1 | Bacteroides ovatus | 1 | 1 | 51/6475296 | 0.0008 | 1 |
| Bacteroides | 0.05 | 1 | 1 | Bacteroides salyersiae | 0 | 0 | 75/5815818 | 0.0013 | 1 |
| Brevibacterium | 0.13 | 1 | 1 | Brevibacterium casei | 1 | 1 | 225/3769980 | 0.006 | 1 |
| Caulobacter | 0.08 | 1 | 1 | Caulobacter crescentus | 1 | 1 | 121/4042929 | 0.003 | 1 |
| Chroococcidiopsis | 0.05 | 1 | 1 | Chroococcidiopsis thermalis | 1 | 1 | 132/6315792 | 0.0021 | 1 |
| Cloacibacterium | 0.06 | 1 | 1 | Cloacibacterium normanense | 1 | 1 | 71/2736686 | 0.0026 | 1 |
| Clostridium | 0.05 | 1 | 1 | Clostridium leptum | 1 | 1 | 75/3270409 | 0.0023 | 1 |
| Collimonas | 0.13 | 1 | 1 | Collimonas fungivorans | 1 | 1 | 157/5186898 | 0.003 | 1 |
| Collimonas | 0.13 | 1 | 1 | Collimonas pratensis | 0 | 0 | 56/5730025 | 0.001 | 1 |
| Comamonas | 0.23 | 1 | 1 | Comamonas aquatica | 1 | 1 | 47/3764434 | 0.0012 | 1 |
| Comamonas | 0.23 | 1 | 1 | Comamonas kerstersii | 0 | 0 | 41/3734555 | 0.0011 | 1 |
| Comamonas | 0.23 | 1 | 1 | Comamonas terrae | 0 | 0 | 185/4717103 | 0.0039 | 1 |
| Comamonas | 0.23 | 1 | 1 | Comamonas testosteroni | 0 | 0 | 75/5373644 | 0.0014 | 1 |
| Enhydrobacter | 0.1 | 1 | 1 | Enhydrobacter aerosaccus | 1 | 1 | 287/6770053 | 0.0042 | 1 |
| Janibacter | 0.05 | 1 | 1 | Janibacter melonis | 1 | 1 | 75/3196938 | 0.0023 | 1 |
| Mizugakiibacter | 0.05 | 1 | 1 | Mizugakiibacter sediminis | 1 | 1 | 45/3122786 | 0.0014 | 1 |
| Neisseria | 0.22 | 1 | 1 | Neisseria perflava | 1 | 1 | 309/3786149 | 0.0082 | 1 |
| Polynucleobacter | 0.39 | 1 | 1 | Polynucleobacter necessarius | 1 | 1 | 312/2159490 | 0.0144 | 1 |
| Pseudoalteromonas | 0.04 | 1 | 1 | Pseudoalteromonas haloplanktis | 1 | 1 | 68/3850272 | 0.0018 | 1 |
| Rhodobacter | 0.24 | 1 | 1 | Rhodanobacter 2APBS1 | 1 | 1 | 394/4225490 | 0.0093 | 1 |
| Rhodopirellula | 0.02 | 1 | 1 | Rhodopirellula baltica | 1 | 1 | 46/7145576 | 0.0006 | 1 |
| Saccharomonospora | 0.04 | 1 | 1 | Saccharomonospora viridis | 1 | 1 | 45/4308349 | 0.001 | 1 |
| Stanieria | 0.03 | 1 | 1 | Stanieria cyanosphaera | 1 | 1 | 53/5041209 | 0.0011 | 1 |
| Veillonella | 0.24 | 1 | 1 | Veillonella atypica | 1 | 1 | 222/2099783 | 0.0106 | 1 |
| Acetobacter | 0.06 | 0 | 0 | Acetobacter pasteurianus | 0 | 0 | 42/2907287 | 0.0014 | 1 |
| Acidisphaera | 0.04 | 0 | 0 | Acidisphaera rubrifaciens | 0 | 0 | 66/3874653 | 0.0017 | 1 |
| Actinomyces | 0.12 | 0 | 0 | Actinomyces odontolyticus | 0 | 0 | 47/2432045 | 0.0019 | 1 |
| Actinomyces | 0.12 | 0 | 0 | Actinomyces viscosus | 0 | 0 | 67/3134536 | 0.0021 | 1 |
| Acuticoccus | 0.03 | 0 | 0 | Acuticoccus yangtzensis | 0 | 0 | 42/5099489 | 0.0008 | 1 |
| Agrobacterium | 0.13 | 0 | 0 | Agrobacterium fabrum | 0 | 0 | 150/4917167 | 0.0031 | 1 |
| Agrobacterium | 0.13 | 0 | 0 | Agrobacterium H13 | 0 | 0 | 147/4972229 | 0.003 | 1 |
| Algibacter | 0.04 | 0 | 0 | Algibacter alginicilyticus | 0 | 0 | 50/3994770 | 0.0013 | 1 |
| Alicycliphilus | 0.03 | 0 | 0 | Alicycliphilus denitrificans | 0 | 0 | 57/4995263 | 0.0011 | 1 |
| Aliterella | 0.03 | 0 | 0 | Aliterella atlantica | 0 | 0 | 45/5266293 | 0.0009 | 1 |
| Aminobacter | 0.03 | 0 | 0 | Aminobacter aminovorans | 0 | 0 | 50/5623946 | 0.0009 | 1 |
| Anabaena | 0.03 | 0 | 0 | Anabaena variabilis | 0 | 0 | 75/6365727 | 0.0012 | 1 |
| Aquimonas | 0.04 | 0 | 0 | Aquimonas voraii | 0 | 0 | 75/4428557 | 0.0017 | 1 |
| Arenibacter | 0.03 | 0 | 0 | Arenibacter certesii | 0 | 0 | 75/4990571 | 0.0015 | 1 |
| Arenimonas | 0.05 | 0 | 0 | Arenimonas malthae | 0 | 0 | 58/3116278 | 0.0019 | 1 |
| Azohydromonas | 0.02 | 0 | 0 | Azohydromonas lata | 0 | 0 | 55/7186661 | 0.0008 | 1 |
| Azorhizobium | 0.03 | 0 | 0 | Azorhizobium doebereinerae | 0 | 0 | 35/5818544 | 0.0006 | 1 |
| Azospira | 0.04 | 0 | 0 | Azospira oryzae | 0 | 0 | 75/3806980 | 0.002 | 1 |
| Azospirillum | 0.11 | 0 | 0 | Azospirillum brasilense | 0 | 0 | 114/3023440 | 0.0038 | 1 |
| Bartonella | 0.11 | 0 | 0 | Bartonella rochalimae | 0 | 0 | 71/1534163 | 0.0046 | 1 |
| Brachymonas | 0.06 | 0 | 0 | Brachymonas denitrificans | 0 | 0 | 75/2713070 | 0.0028 | 1 |
| Bradyrhizobium | 0.22 | 0 | 0 | Bradyrhizobium oligotrophicum | 0 | 0 | 101/8264165 | 0.0012 | 1 |
| Bradyrhizobium | 0.22 | 0 | 0 | Bradyrhizobium ORS | 0 | 0 | 105/7456587 | 0.0014 | 1 |
| Bradyrhizobium | 0.22 | 0 | 0 | Bradyrhizobium S23321 | 0 | 0 | 408/7231841 | 0.0056 | 1 |
| Brucella | 0.05 | 0 | 0 | Brucella inopinata | 0 | 0 | 49/3367314 | 0.0015 | 1 |
| Caldimonas | 0.09 | 0 | 0 | Caldimonas manganoxidans | 0 | 0 | 100/3532996 | 0.0028 | 1 |
| Calothrix | 0.02 | 0 | 0 | Calothrix PCC | 0 | 0 | 75/7023215 | 0.0011 | 1 |
| Capnocytophaga | 0.06 | 0 | 0 | Capnocytophaga gingivalis | 0 | 0 | 75/2667498 | 0.0028 | 1 |
| Carnobacterium | 0.06 | 0 | 0 | Carnobacterium alterfunditum | 0 | 0 | 52/2616930 | 0.002 | 1 |
| Celeribacter | 0.04 | 0 | 0 | Celeribacter ethanolicus | 0 | 0 | 39/4009276 | 0.001 | 1 |
| Chitinophaga | 0.02 | 0 | 0 | Chitinophaga pinensis | 0 | 0 | 49/9127347 | 0.0005 | 1 |
| Chlorogloeopsis | 0.02 | 0 | 0 | Chlorogloeopsis fritschii | 0 | 0 | 71/7753345 | 0.0009 | 1 |
| Chromobacterium | 0.07 | 0 | 0 | Chromobacterium haemolyticum | 0 | 0 | 72/5030819 | 0.0014 | 1 |
| Chromobacterium | 0.07 | 0 | 0 | Chromobacterium violaceum | 0 | 0 | 68/4751080 | 0.0014 | 1 |
| Chryseobacterium | 0.05 | 0 | 0 | Chryseobacterium molle | 0 | 0 | 65/3695475 | 0.0018 | 1 |
| Cnuibacter | 0.04 | 0 | 0 | Cnuibacter physcomitrellae | 0 | 0 | 55/4061502 | 0.0014 | 1 |
| Cronobacter | 0.04 | 0 | 0 | Cronobacter condimenti | 0 | 0 | 49/4347991 | 0.0011 | 1 |
| Curvibacter | 0.04 | 0 | 0 | Curvibacter delicatus | 0 | 0 | 51/3756831 | 0.0014 | 1 |
| Cylindrospermum | 0.02 | 0 | 0 | Cylindrospermum stagnale | 0 | 0 | 48/7003560 | 0.0007 | 1 |
| Defluviimonas | 0.04 | 0 | 0 | Defluviimonas alba | 0 | 0 | 75/4738298 | 0.0016 | 1 |
| Duganella | 0.02 | 0 | 0 | Duganella sacchari | 0 | 0 | 51/6676036 | 0.0008 | 1 |
| Dyella | 0.31 | 0 | 0 | Dyella japonica | 0 | 0 | 561/4831185 | 0.0116 | 1 |
| Edaphobacter | 0.02 | 0 | 0 | Edaphobacter aggregans | 0 | 0 | 63/8180575 | 0.0008 | 1 |
| Escherichia | 0.16 | 0 | 0 | Escherichia coli | 0 | 0 | 297/5399183 | 0.0055 | 1 |
| Escherichia | 0.16 | 0 | 0 | Escherichia fergusonii | 0 | 0 | 75/4588711 | 0.0016 | 1 |
| Flectobacillus | 0.03 | 0 | 0 | Flectobacillus major | 0 | 0 | 61/6221090 | 0.001 | 1 |
| Fuerstia | 0.02 | 0 | 0 | Fuerstia marisgermanicae | 0 | 0 | 75/8920478 | 0.0008 | 1 |
| Fulvivirga | 0.02 | 0 | 0 | Fulvivirga imtechensis | 0 | 0 | 65/6740114 | 0.001 | 1 |
| Hafnia | 0.03 | 0 | 0 | Hafnia paralvei | 0 | 0 | 71/4998684 | 0.0014 | 1 |
| Halapricum | 0.05 | 0 | 0 | Halapricum salinum | 0 | 0 | 31/3451512 | 0.0009 | 1 |
| Halioglobus | 0.04 | 0 | 0 | Halioglobus japonicus | 0 | 0 | 47/4085301 | 0.0012 | 1 |
| Immundisolibacter | 0.05 | 0 | 0 | Immundisolibacter cernigliae | 0 | 0 | 38/3243537 | 0.0012 | 1 |
| Janthinobacterium | 0.04 | 0 | 0 | Janthinobacterium Marseille | 0 | 0 | 40/4110251 | 0.001 | 1 |
| Kineosphaera | 0.03 | 0 | 0 | Kineosphaera limosa | 0 | 0 | 70/4848887 | 0.0014 | 1 |
| Knoellia | 0.04 | 0 | 0 | Knoellia aerolata | 0 | 0 | 75/4090260 | 0.0018 | 1 |
| Kribbella | 0.02 | 0 | 0 | Kribbella flavida | 0 | 0 | 71/7579488 | 0.0009 | 1 |
| Leeia | 0.04 | 0 | 0 | Leeia oryzae | 0 | 0 | 48/3785675 | 0.0013 | 1 |
| Leifsonia | 0.06 | 0 | 0 | Leifsonia xyli | 0 | 0 | 72/2686418 | 0.0027 | 1 |
| Leptospira | 0.04 | 0 | 0 | Leptospira meyeri | 0 | 0 | 59/4188121 | 0.0014 | 1 |
| Leucobacter | 0.1 | 0 | 0 | Leucobacter celer | 0 | 0 | 75/4143510 | 0.0018 | 1 |
| Leucobacter | 0.1 | 0 | 0 | Leucobacter chironomi | 0 | 0 | 75/2964972 | 0.0025 | 1 |
| Limimonas | 0.05 | 0 | 0 | Limimonas halophila | 0 | 0 | 45/3039611 | 0.0015 | 1 |
| Luteipulveratus | 0.04 | 0 | 0 | Luteipulveratus halotolerans | 0 | 0 | 72/4464306 | 0.0016 | 1 |
| Lysinimicrobium | 0.06 | 0 | 0 | Lysinimicrobium flavum | 0 | 0 | 65/2985075 | 0.0022 | 1 |
| Lysobacter | 0.03 | 0 | 0 | Lysobacter antibioticus | 0 | 0 | 75/5916388 | 0.0013 | 1 |
| Maricaulis | 0.05 | 0 | 0 | Maricaulis maris | 0 | 0 | 75/3368780 | 0.0022 | 1 |
| Marivirga | 0.04 | 0 | 0 | Marivirga tractuosa | 0 | 0 | 67/4511574 | 0.0015 | 1 |
| Marmoricola | 0.04 | 0 | 0 | Marmoricola aequoreus | 0 | 0 | 72/4163916 | 0.0017 | 1 |
| Massilia | 0.03 | 0 | 0 | Massilia timonae | 0 | 0 | 41/6136630 | 0.0007 | 1 |
| Mastigocladopsis | 0.05 | 0 | 0 | Mastigocladopsis repens | 0 | 0 | 141/6465675 | 0.0022 | 1 |
| Mesorhizobium | 0.1 | 0 | 0 | Mesorhizobium loti | 0 | 0 | 182/7036071 | 0.0026 | 1 |
| Mesorhizobium | 0.1 | 0 | 0 | Mesorhizobium opportunistum | 0 | 0 | 57/6884444 | 0.0008 | 1 |
| Methyloversatilis | 0.08 | 0 | 0 | Methyloversatilis discipulorum | 0 | 0 | 109/4304808 | 0.0025 | 1 |
| Methylovorus | 0.11 | 0 | 0 | Methylovorus glucosetrophus | 0 | 0 | 65/2995511 | 0.0022 | 1 |
| Methylovorus | 0.11 | 0 | 0 | Methylovorus MP688 | 0 | 0 | 64/2862391 | 0.0022 | 1 |
| Microlunatus | 0.02 | 0 | 0 | Microlunatus soli | 0 | 0 | 54/6733014 | 0.0008 | 1 |
| Modestobacter | 0.03 | 0 | 0 | Modestobacter marinus | 0 | 0 | 49/5575517 | 0.0009 | 1 |
| Nocardia | 0.02 | 0 | 0 | Nocardia pseudobrasiliensis | 0 | 0 | 56/8875408 | 0.0006 | 1 |
| Nocardioides | 0.03 | 0 | 0 | Nocardioides JS614 | 0 | 0 | 75/4985871 | 0.0015 | 1 |
| Nocardiopsis | 0.02 | 0 | 0 | Nocardiopsis synnemataformans | 0 | 0 | 69/7362964 | 0.0009 | 1 |
| Nostoc | 0.04 | 0 | 0 | Nostoc punctiforme | 0 | 0 | 115/8234322 | 0.0014 | 1 |
| Novosphingobium | 0.04 | 0 | 0 | Novosphingobium PP1Y | 0 | 0 | 72/3911486 | 0.0018 | 1 |
| Pandoraea | 0.09 | 0 | 0 | Pandoraea apista | 0 | 0 | 58/5609637 | 0.001 | 1 |
| Pandoraea | 0.09 | 0 | 0 | Pandoraea pnomenusa | 0 | 0 | 63/5584076 | 0.0011 | 1 |
| Pandoraea | 0.09 | 0 | 0 | Pandoraea sputorum | 0 | 0 | 47/5742997 | 0.0008 | 1 |
| Parvibaculum | 0.04 | 0 | 0 | Parvibaculum lavamentivorans | 0 | 0 | 46/3914745 | 0.0012 | 1 |
| Phenylobacterium | 0.04 | 0 | 0 | Phenylobacterium zucineum | 0 | 0 | 75/3996255 | 0.0019 | 1 |
| Pseudonocardia | 0.02 | 0 | 0 | Pseudonocardia dioxanivorans | 0 | 0 | 54/7096571 | 0.0008 | 1 |
| Pseudoramibacter | 0.07 | 0 | 0 | Pseudoramibacter alactolyticus | 0 | 0 | 63/2363292 | 0.0027 | 1 |
| Pseudovibrio | 0.03 | 0 | 0 | Pseudovibrio FO | 0 | 0 | 62/5475670 | 0.0011 | 1 |
| Pseudoxanthomonas | 0.09 | 0 | 0 | Pseudoxanthomonas Mexicana | 0 | 0 | 63/3943279 | 0.0016 | 1 |
| Pseudoxanthomonas | 0.09 | 0 | 0 | Pseudoxanthomonas spadix | 0 | 0 | 58/3452554 | 0.0017 | 1 |
| Psychrobacter | 0.05 | 0 | 0 | Psychrobacter G | 0 | 0 | 75/3079438 | 0.0024 | 1 |
| Rhizobium | 0.15 | 0 | 0 | Rhizobium etli | 0 | 0 | 70/4598466 | 0.0015 | 1 |
| Rhizobium | 0.15 | 0 | 0 | Rhizobium IRBG74 | 0 | 0 | 120/2844565 | 0.0042 | 1 |
| Rothia | 0.07 | 0 | 0 | Rothia mucilaginosa | 0 | 0 | 46/2264603 | 0.002 | 1 |
| Rubrivivax | 0.07 | 0 | 0 | Rubrivivax gelatinosus | 0 | 0 | 124/5043253 | 0.0025 | 1 |
| Serratia | 0.09 | 0 | 0 | Serratia marcescens | 0 | 0 | 120/5471439 | 0.0022 | 1 |
| Serratia | 0.09 | 0 | 0 | Serratia proteamaculans | 0 | 0 | 52/5448853 | 0.001 | 1 |
| Shigella | 0.07 | 0 | 0 | Shigella dysenteriae | 0 | 0 | 75/4369232 | 0.0017 | 1 |
| Shigella | 0.07 | 0 | 0 | Shigella flexneri | 0 | 0 | 75/4698633 | 0.0016 | 1 |
| Sinorhizobium | 0.02 | 0 | 0 | Sinorhizobium meliloti | 0 | 0 | 34/6813723 | 0.0005 | 1 |
| Starkeya | 0.07 | 0 | 0 | Starkeya novella | 0 | 0 | 82/4765023 | 0.0017 | 1 |
| Sulfuricella | 0.05 | 0 | 0 | Sulfuricella denitrificans | 0 | 0 | 68/3130594 | 0.0022 | 1 |
| Sulfuricurvum | 0.06 | 0 | 0 | Sulfuricurvum kujiense | 0 | 0 | 64/2574824 | 0.0025 | 1 |
| Variovorax | 0.02 | 0 | 0 | Variovorax paradoxus | 0 | 0 | 75/7148516 | 0.001 | 1 |
| Verminephrobacter | 0.03 | 0 | 0 | Verminephrobacter eiseniae | 0 | 0 | 62/5566749 | 0.0011 | 1 |

Note: Genus Re Abu: Relative abundance of Genus; SMRNG: Stringent mapped reads number of genus; SDSMRNG: Standard Stringent mapped reads number of genus; SMRN: Stringent mapped reads number; SDSMRN: Standard Stringent mapped reads number; CovRate: Coverage rate.

Table 2. Microbe reads of fungi, parasite and virus detected in Case No. 4

| Genus | Genus Re Abu | SMRNG | SDSMRNG | Species | SMRN | SDSMRN | Coverage | CovRate | Depth |
| --- | --- | --- | --- | --- | --- | --- | --- | --- | --- |
| Malassezia | 88.36 | 47 | 57 | Malassezia globosa | 47 | 57 | 3556/8872979 | 0.0401 | 1 |
| Botrytis | 0.69 | 2 | 2 | Botryotinia fuckeliana | 2 | 2 | 141/42749700 | 0.0003 | 1 |
| Debaryomyces | 3.64 | 2 | 2 | Debaryomyces hansenii | 2 | 2 | 201/12182018 | 0.0016 | 1 |
| Rhodotorula | 1.48 | 2 | 2 | Rhodotorula mucilaginosa | 2 | 2 | 150/19992149 | 0.0008 | 1 |
| Chaetomium | 0.42 | 1 | 1 | Chaetomium globosum | 1 | 1 | 75/34887299 | 0.0002 | 1 |
| Enterocytozoon | 3.81 | 0 | 0 | Enterocytozoon bieneusi | 0 | 0 | 54/3878058 | 0.0014 | 1 |
| Malassezia | 88.36 | 47 | 57 | Malassezia pachydermatis | 0 | 0 | 72/8129607 | 0.0009 | 1 |
| Phanerochaete | 0.49 | 0 | 0 | Phanerochaete chrysosporium | 0 | 0 | 44/29855776 | 0.0001 | 1 |
| Sordaria | 1.11 | 0 | 0 | Sordaria macrospora | 0 | 0 | 225/40002837 | 0.0006 | 1 |
| Gongylonema | 3.27 | 2 | 2 | Gongylonema pulchrum | 2 | 2 | 118/290924066 | 0 | 1 |
| Trypanosoma | 27.99 | 1 | 1 | Trypanosoma congolense | 1 | 1 | 75/16947329 | 0.0004 | 1 |
| Wuchereria | 6.17 | 1 | 1 | Wuchereria bancrofti | 1 | 1 | 75/77159569 | 0.0001 | 1 |
| Acanthamoeba | 34.72 | 2 | 2 | Acanthamoeba lenticulata | 0 | 0 | 61/59662351 | 0.0001 | 1 |
| Acanthamoeba | 34.72 | 2 | 2 | Acanthamoeba palestinensis | 0 | 0 | 90/93383381 | 0.0001 | 1 |
| Acanthamoeba | 34.72 | 2 | 2 | Acanthamoeba triangularis | 0 | 0 | 200/85627420 | 0.0002 | 1 |
| Plasmodium | 16.46 | 0 | 0 | Plasmodium malariae | 0 | 0 | 49/28786109 | 0.0002 | 1 |
| Schistosoma | 1.28 | 0 | 0 | Schistosoma mansoni | 0 | 0 | 41/364541682 | 0 | 1 |
| Taenia | 3.13 | 0 | 0 | Taenia asiatica | 0 | 0 | 61/151825899 | 0 | 1 |
| Trichuris | 6.97 | 0 | 0 | Trichuris trichiura | 0 | 0 | 75/67961556 | 0.0001 | 1 |
| - | - | - | - | Human alphaherpesvirus 3  (Varicella zoster virus) | 84 | 102 | 5392/124884 | 4.32 | 1.03 |
| - | - | - | - | Human parvovirus B19 | 3 | 4 | 414/5596 | 7.4 | 1 |

Note: Genus Re Abu: Relative abundance of Genus; SMRNG: Stringent mapped reads number of genus; SDSMRNG: Standard Stringent mapped reads number of genus; SMRN: Stringent mapped reads number; SDSMRN: Standard Stringent mapped reads number; CovRate: Coverage rate.
