# Supplementary material for: Clinical features of aseptic meningitis with varicella zoster virus infection diagnosed by next-generation sequencing: case reports
Source: BMC Infect Dis. 2020 Jun 22;20:435. doi: 10.1186/s12879-020-05155-8 (PMC7309994; doi:10.1186/s12879-020-05155-8)
Supplement: Supplementary file 6 — Additional file 6. Original images for results of Sanger sequencing detection of VZV from CSF specimen [file 12879_2020_5155_MOESM6_ESM.docx]

**Additional file 6:** Original images for results of Sanger sequencing detection of VZV from CSF specimen.


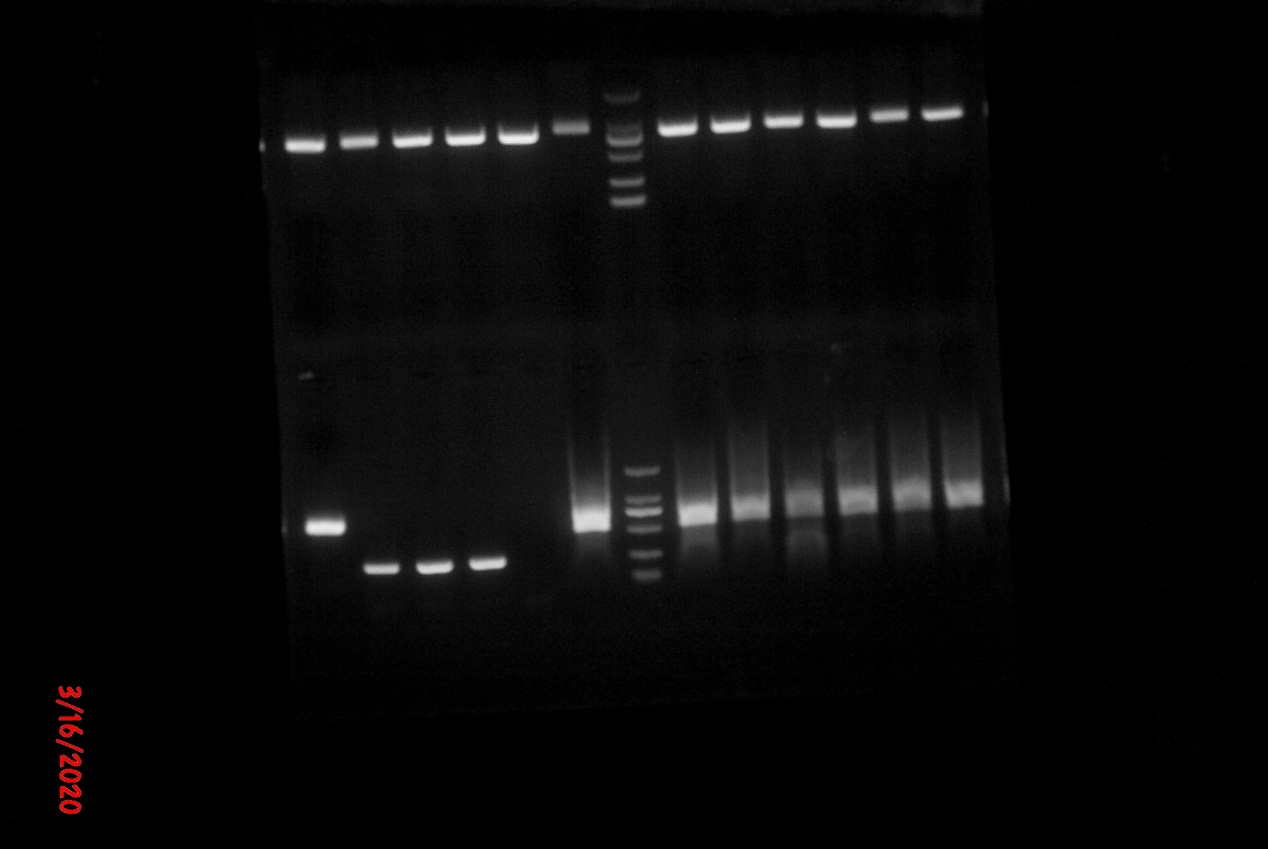


277

277

M

N

Case 2

Case 1

-500bp

-250bp

-100bp

a


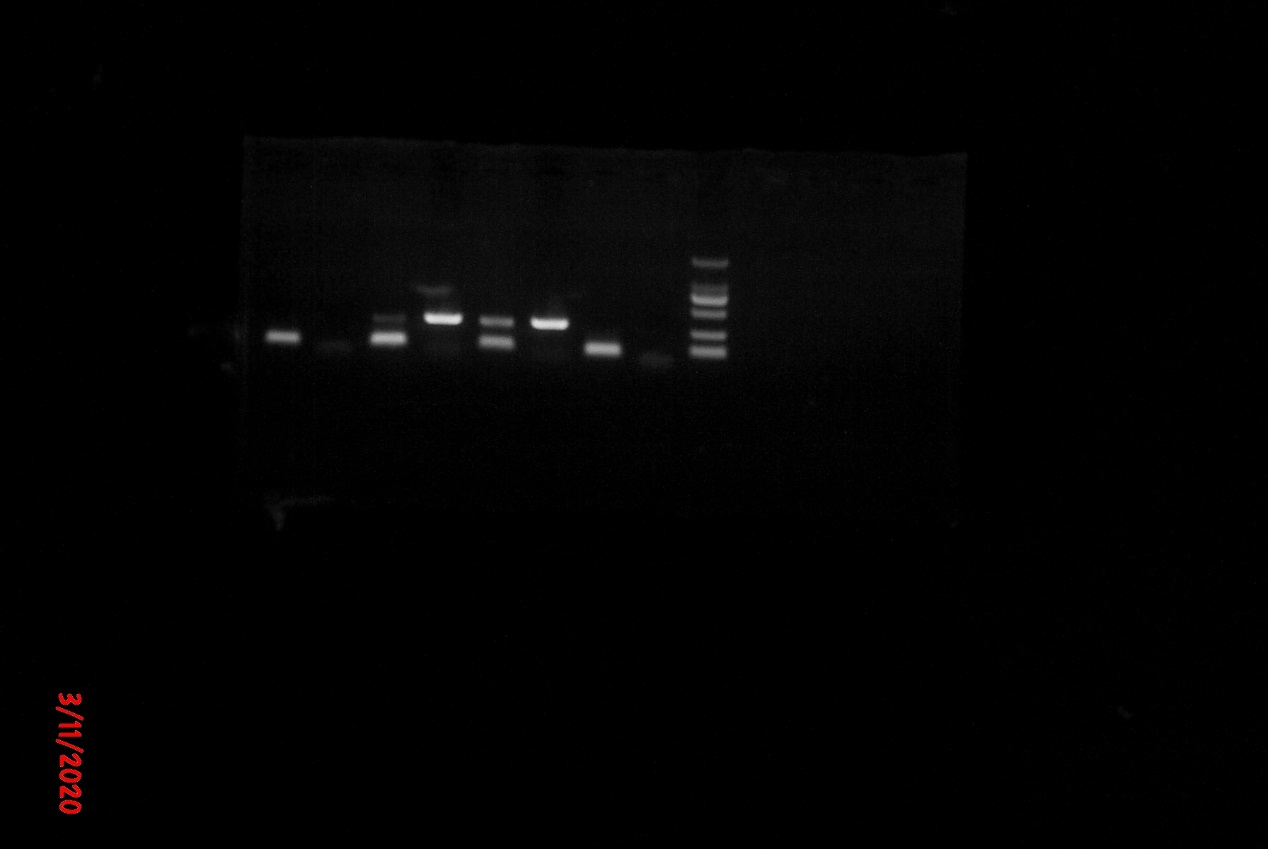


Case 3

Case 4

277

277

N

M

-500bp

-250bp

-100bp

b

**Fig. 2 Sanger sequencing detection of VZV from CSF specimen.**

M: DNA markers of DL2000 or Trans 2K Plus. N: negative control. The number 277bp represent the sample code.
